# Immunoglobulin sub-class levels define inter-donor plasma variability: a longitudinal dual-lab study

**DOI:** 10.1038/s44320-026-00218-5

**Published:** 2026-05-26

**Authors:** Stephan Michalik, Sofia Kalaidopoulou Nteak, Nicolas Drouin, Manuela Gesell Salazar, Elke Hammer, Vishnu Mukund Dhople, Silva Holtfreter, Stefan Weiss, Henk W P van den Toorn, Barbara M Bröker, Grażyna Domańska, Uwe Völker, Albert J R Heck

**Affiliations:** 1 Interfaculty Institute of Genetics and Functional Genomics, Department Functional Genomics, University Medicine Greifswald, Greifswald, Germany; 2 German Center for Cardiovascular Research (DZHK), partner site North (Greifswald), Greifswald, Germany; 3 Biomolecular Mass Spectrometry and Proteomics, Bijvoet Centre for Biomolecular Research and Utrecht Institute for Pharmaceutical Sciences, Utrecht University, Utrecht, the Netherlands; 4 Netherlands Proteomics Center, Utrecht, the Netherlands; 5 Institute of Immunology, University Medicine Greifswald, Greifswald, Germany

**Keywords:** Proteomics

## Abstract

Advances in mass spectrometry have transformed plasma proteomics, allowing high-throughput analysis of large cohorts. This study utilized the TIMES (Tracking Individuals Monthly for Evaluating Stability) cohort, consisting of 51 healthy participants monitored monthly over 12 months, to evaluate intra- and inter-individual variability in the plasma proteome. Approximately 600 samples were analyzed in two laboratories independently, revealing strong correlations despite methodological differences. The study revealed high stability of the plasma proteome within donors over a year, with larger differences observed between donors. A support vector machine model achieved a 98% median classification accuracy, confirming stable, donor-specific proteomic profiles. Immunoglobulins, often overlooked in plasma proteomics, were found to contribute significantly to donor-specific signatures, showing pronounced inter-individual variability but remarkable intra-donor stability. In contrast, C-reactive protein and other inflammation markers exhibited significant temporal fluctuations from donor-specific baseline levels. This inter-laboratory study highlights the importance of longitudinal sampling for biomarker discovery, the robustness of MS-based proteomic workflows, and provides new insights into immunoglobulin dynamics and variability in human plasma.

## Synopsis

Longitudinal analysis over a one-year period defines a stable yet highly donor-specific human plasma proteome. Immunoglobulin subclasses drive the inter-individual variability, while cross-laboratory consistency confirms analytical robustness.

- Inter-site laboratory comparisons confirmed strong concordance in analytical performance and measurement consistency.
- Conversion of relative protein intensities to absolute concentrations revealed immunoglobulin levels on par with established clinical reference ranges.
- Donors were distinguishable based on their unique plasma proteome.
- The immunoglobulin subclasses are key discriminators defining inter- and intra-donor variability.

## Abstract (web summary)

Longitudinal analysis over a one-year period defines a stable yet highly donor-specific human plasma proteome. Immunoglobulin subclasses drive the inter-individual variability, while cross-laboratory consistency confirms analytical robustness.

## Graphical abstract

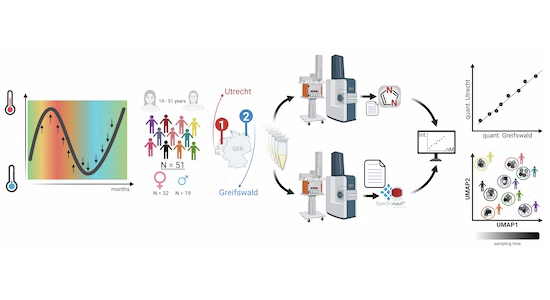

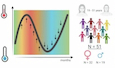

## Introduction

Due to the relatively non-invasive nature of sampling, blood plasma plays a central role in clinical diagnostics and therapeutic monitoring (Geyer et al, 2017; Álvez et al, 2025). However, the plasma proteome represents one of the analytically most demanding proteomes, characterized by its vast protein diversity and extended dynamic range (Anderson, 2002). The inherent complexity of the plasma proteome reflects its multifaceted biological origins. Circulating proteins originate from multiple sources: hepatocytes secrete the majority of plasma proteins, lymphocytes produce immunoglobulins and cytokines, or proteins entering the circulation through tissue-damage-induced leakage (Zhang et al, 2007). This complexity is further amplified by the polymorphic nature of several plasma proteins, most notably the immunoglobulins, (Nimmerjahn et al, 2023). Furthermore, concentrations of plasma proteins span a range of over ten orders of magnitude, e.g., from highly abundant proteins such as albumin, accounting for 55% of the total protein content (Anderson, 2002), and immunoglobulins (about 20% (Patel et al, 2023)), down to trace-level cytokines (Geyer et al, 2017; Ameling et al, 2025).

Traditionally, blood protein analysis relies on targeted techniques such as ELISA, which enable robust and sensitive quantification of predefined protein targets within complex mixtures. In contrast, mass spectrometry (MS) offers a powerful alternative for both diagnostic applications and biomarker discovery, as protein identification, quantification, and characterization are performed (Aebersold and Mann, 2016). Recent advancements in MS have improved the analytical capabilities of plasma proteomics. Enhanced sensitivity, expanded dynamic range of mass spectrometry devices (Vitko et al, 2024; Hendricks et al, 2024), and refined sample preparation protocols now enable the differentiation of physiological or pathological states, which is essential for disease screening, diagnosis, therapeutic stratification, and monitoring treatment efficacy. Despite substantial investment in biomarker research, the clinical translation of novel protein biomarkers remains limited. While FDA approvals have incorporated an increasing number of distinct cancer biomarkers, growing from 3 in 2011 to 16 in 2022 (Dou et al, 2024), the pace of new biomarker adoption remains modest relative to the scale of research investment. A major barrier to clinical translation is the lack of standardization across the biomarker discovery pipeline: challenges in sample collection, pre-analytical handling, measurement platforms, and data processing hinder cross-study integration and limit the generation of robust, comparable results across laboratories, cohorts, and populations (Cai et al, 2025). These translational bottlenecks highlight the necessity for a robust and integrated screening pipeline and workflows for biomarker discovery research and clinical proteomics (Völlmy et al, 2021; Messner et al, 2020). Recent comparative analyses of 16 distinct proteomic sample preparation methodologies (Varnavides et al, 2022), evaluated in HeLa cell lysates, revealed that most preparation protocols yielded comparable protein extraction efficiencies, demonstrated high inter-method reproducibility, and exhibited minimal levels of preparation-induced artifacts. These findings emphasize that, despite the inherent methodological variability in proteomics sample preparation and analytical workflows, harmonization is achievable and can yield consistent results across laboratories and studies.

Here, we set out to empirically validate this concept of harmonization, making use of the TIMES (Tracking Individuals Monthly for Evaluating Stability) cohort. This cohort comprises 51 (supposedly) healthy participants from whom blood was retrieved monthly over a period of 12 months, resulting in about ~600 samples. This allowed us to assess intra- and inter-individual variability in the plasma proteome in a healthy cohort. We independently processed and analyzed the healthy donor cohort at two separate laboratories in Germany and the Netherlands, employing distinct sample preparation protocols, mass spectrometry platforms and initial raw data analysis workflows.

## Results

Over the past decades, advancements in mass spectrometry have transformed the landscape of plasma proteomics. These technological developments have increased both sample throughput and proteomics depth, paving the way for the analysis of much larger cohorts in plasma proteomics biomarker research. Despite these innovations, many biomarker studies continue to rely on cross-sectional designs, typically measuring only a single time point per subject. This approach overlooks the potentially valuable insights provided by longitudinal sampling, which can reveal donor-specific features and temporal variations critical for understanding dynamic biological processes.

**Figure 1:**
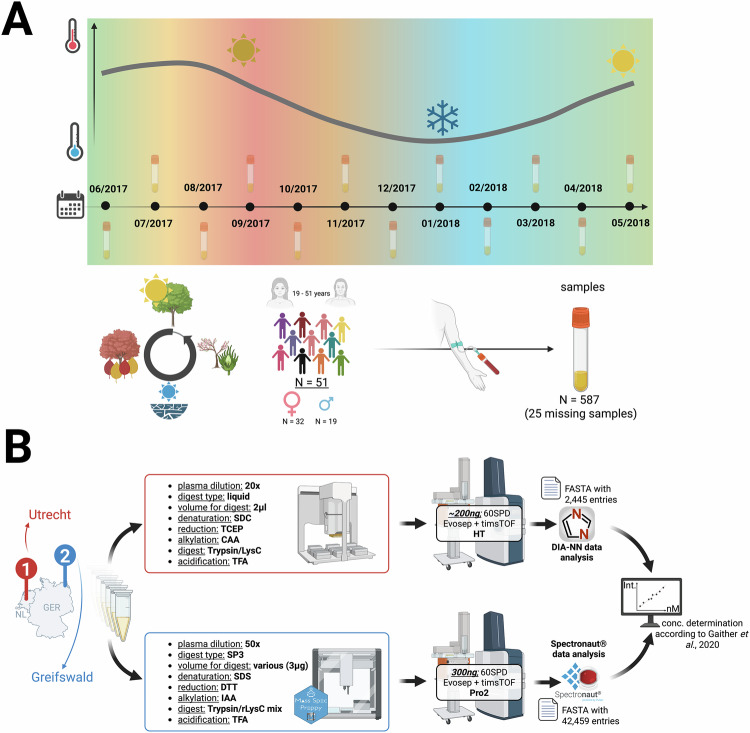
Study workflows. Schematic representation of the TIMES Cohort (**A**) and plasma proteome analysis workflow parameters for each laboratory site (**B**).

**Figure EV1:**
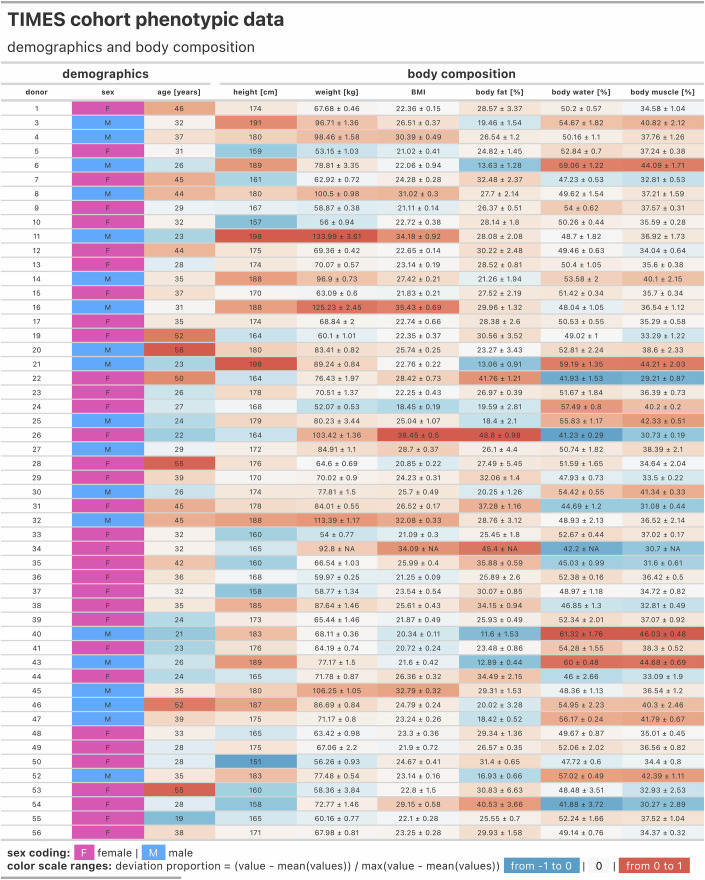
TIMES cohort - donor phenotypic data.

To demonstrate the potential of longitudinal sampling, the TIMES cohort (tracking Individuals monthly for evaluating stability) was established (Fig. 1A; Appendix Table S2; Fig. EV1). This cohort of 51 participants, consisting of 32 females (62.7%) and 19 males (37.3%), was monitored at monthly intervals over a 12-month period. This high-frequency longitudinal sampling of blood enabled assessment of both intra- and inter-individual variability, as well as the temporal stability of candidate plasma biomarkers across the study duration (Richard et al, 2024). The supposedly healthy participants had a mean age of 34.33 years (SD = 9.96; range: 19–56 years) and a mean body mass index (BMI) of 24.58 kg/m^2^ (SD = 4.36; range: 18.4–39.8 kg/m^2^) (Appendix Table S2).

To enable inter-laboratory comparisons, ~600 plasma samples from the TIMES cohort were analyzed using two independent mass spectrometry workflows at two laboratory sites (Fig. 1B). This design reflects the challenges of data comparison between different sites in clinical proteomics, where methodological differences including digestion protocols—liquid phase versus bead-based, protein normalized samples versus fixed volume digestion, the sample injection amount, and mass spectrometry platforms were employed. Beyond these experimental factors, data processing introduced further complexity; choices regarding raw data analysis software (e.g., DIA-NN (Demichev et al, 2020) versus Spectronaut^®^ (Bruderer et al, 2015)), protein database sizes and constructions, and protein quantification methods, such as e.g., intensity-based absolute quantification (iBAQ) (Schwanhäusser et al, 2011) versus MaxLFQ (Cox et al, 2014), were also made independently by each site. Collectively, these layers of methodological variability highlight the inherent challenges in harmonizing proteomic datasets generated across diverse experimental and analytical frameworks.

In this study, the Utrecht site employed a dedicated compact plasma-specific protein database comprised of just 2445 entries in order to avoid false discoveries and decrease search time. This database was built from expected serum proteins labeled as “quantified in plasma” in Human Protein Atlas (Uhlén et al, 2015), to which the top 5% most frequent single amino acid mutations of these proteins, as reported within the NextProt database, were added as a new entry with an identical gene for compatibility and processing with DIA-NN. In parallel, the Greifswald site utilized a substantially larger human protein database containing 42,459 entries (canonical sequences plus isoforms) in combination with Spectronaut^®^. This also illustrates the diversity of laboratory-specific workflows for plasma proteomics worldwide and, in principle, would be expected to produce comparable results when analyzing identical samples.

Understanding the impact of specific methodological decisions on data quality is therefore critical for optimizing proteomics analysis workflows. To convert relative quantitative data into more clinically relevant concentration values, absolute quantification data for 37 housekeeping plasma proteins as reported by Gaither et al (Gaither et al, 2020) were utilized (Appendix Table S1). These proteins were selected based on the following criteria: a coefficient of variation (CV) less than 0.3 in the Gaither et al dataset (Gaither et al, 2020), a CV below 0.3 across the X-omics cohort (*n* = 150) (Bizzarri et al, 2025), and suppression of proteins involved in coagulation pathways to ensure consistency and reliability for both plasma and serum samples. Using these blood plasma housekeeping proteins as a reference of known absolute concentration, a log_10_ linear regression was built based on a pooled serum sample of 400 healthy donors at the Utrecht Site, whereas the Greifswald site independently built the regression individually for every donor. Linear modeling of absolute protein concentrations (either g/L or nM) against relative protein intensities (iBAQ or MaxLFQ) across different acquisition sites demonstrated that iBAQ intensities yielded superior performance regarding the linear model performance in the timsTOF Pro2 dataset, whereas MaxLFQ intensities were more effective in the timsTOF HT dataset (Appendix Fig. S2). These findings highlight the critical impact of protein quantification and aggregation strategies on the accuracy of absolute concentration estimations. Subsequent analyses were conducted using nanomolar concentration values in combination with iBAQ intensities for the Greifswald site (timsTOF Pro2 dataset) and MaxLFQ intensities for the Utrecht site (timsTOF HT dataset). These combinations were selected based on higher *R*^2^ values observed in the respective linear models, indicating superior linearity and model performance. Differences in protein quantification per sample, which might arise from a combination of workflow-specific parameters, reference sample used for the generation of the calibration line, injection amounts and the dynamic range of the mass spectrometer, resulted in variations for each protein depending on its abundance and detectability within the particular workflow employed.

Overall, after filtering out contaminants and hits containing the variable immunoglobulin regions, 274 proteins at the Greifswald site and 188 proteins at the Utrecht site, could be quantified, with an overlap of 163 proteins. The variations in numbers of proteins covered at both sites reflect the combination of differences in injection amount, sensitivity of MS device, FASTA database composition, and raw data analysis tools.

**Figure 2:**
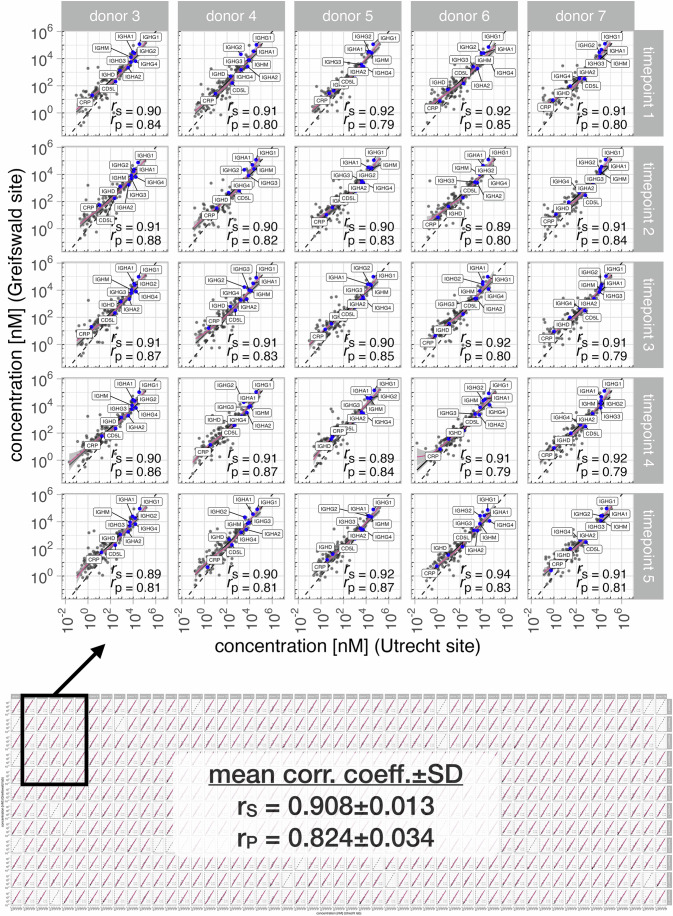
Correlation analysis across lab sites per sample. The overview (lower part) and zoom-in (upper part) panels present a scatterplot depicting the per-sample measurements between the two lab sites, with Utrecht concentration values on the x-axis and Greifswald concentration values on the y-axis. IgG1-4, IgA1-2, IgD, IgM, CD5L, and CRP are highlighted in blue points and labels. Spearman and Pearson correlation coefficients (rounded to two decimal places) are shown as r_s_ and r_p_, respectively.

This stringent filtering led to a rather small subset of genuine plasma proteins, but it still spanned five orders in dynamic range and gave us a large enough dataset to make a sound comparison between the labs for quantitative robustness and reliability. Comparison of protein quantifications between the two laboratory sites on a sample-by-sample basis revealed a strong correlation. Direct pairwise analysis showed that the mean Spearman correlation coefficient across samples was 0.908, while the mean Pearson correlation coefficient was 0.824, highlighting both the consistency of relative protein ranking and the major linear agreement in absolute protein abundances between sites (Fig. 2). The timsTOF Pro2 data from the Greifswald site exhibited a lower dynamic range in the lower protein concentration range when compared directly with the timsTOF HT data from the Utrecht site.

**Figure 3:**
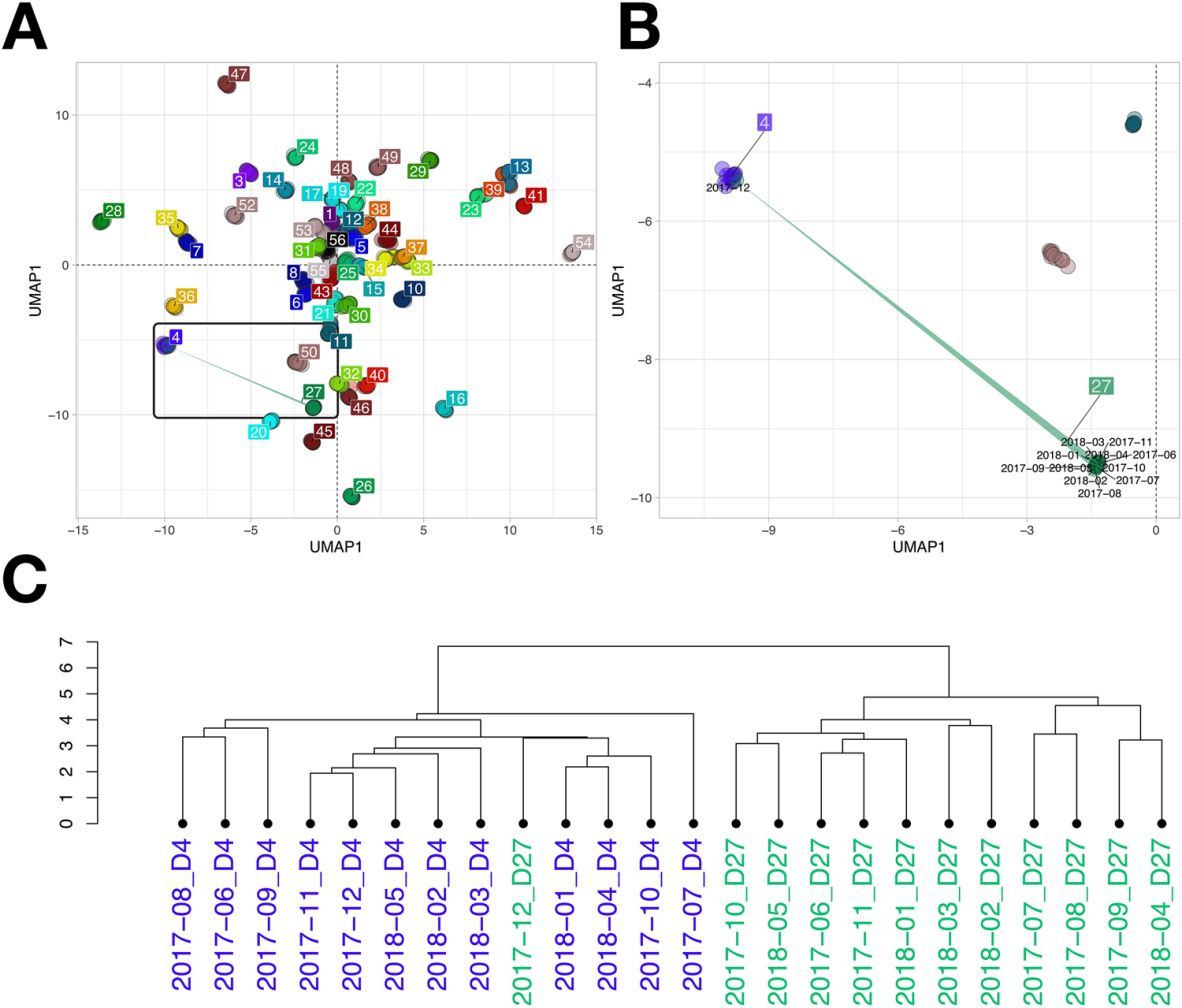
Donor-specific protein profiles. Panel (**A**) presents a global UMAP projection of plasma protein concentrations, encompassing all donors and their respective monthly samples within the TIMES cohort. Each dot symbolizes a monthly sample, uniquely colored to represent the individual donor. Panel (**B**) provides a magnified view of the region highlighted in Panel (**A**), revealing that a sample labeled as donor 27 is embedded within the cluster of donor 4, raising the possibility of sample mislabeling or duplication. Panel (**C**) presents hierarchical clustering of protein concentration profiles for all time points of both donors, computed using Euclidean distance and complete linkage, further supporting the observed proximity in the reduced-dimensional space.

Given the strong correlation observed between the two datasets, we proceeded with some analyses conducted on the Utrecht site dataset for illustrative purposes, as it benefits from superior quantification performance due to the timsTOF HT mass spectrometry device employed. However, similar key findings here described were observed across both sites, supporting the representativeness of the Utrecht data. As a reminder, for each donor, we recorded 12 plasma proteomes originating from the monthly longitudinal sampling. Uniform Manifold Approximation and Projection (UMAP) analysis of the dataset (Fig. 3A) revealed a clear separation between all donors, alongside a consistent protein abundance pattern across monthly sampling for each donor. UMAP is an unsupervised dimensional reduction method that preserves the neighborhood structure of the original high-dimensional data, enabling the visualization of complex relationships in two dimensions. In this context, points that cluster closely in the projection represent samples with highly similar protein abundance profiles across all measured variables prior to dimensional reduction. Accordingly, samples positioned in close proximity within the UMAP plot can be interpreted as sharing comparable protein abundance patterns. In other words, this analysis hints at substantially more inter-donor variation than intra-donor variation. In this context, the longitudinal sampling design of the cohort, combined with the clear donor-specific clustering observed in the UMAP projection, enabled the detection of a potential sample duplication event possibly arising during the aliquoting process.

Notably, the December sample of donor 27 was positioned within the point cloud corresponding to donor 4, indicating a protein abundance profile that was indistinguishable from that donor’s longitudinal samples (Fig. 3B,C). Given the otherwise robust separation of donors across all time points, this overlap was deemed highly unlikely to occur by chance and was interpreted as evidence of a sample mislabeling or duplication. To avoid introducing artefacts into downstream analyses, this outlier sample was excluded from all subsequent analyses. To further validate the observation that each donor exhibits a unique and reproducible intra-donor protein profile, enabling donor identification based on the quantitative data, we employed a multi-class SVM classification model (Appendix Fig. S3), which is a robust methodology for evaluating the ability to distinguish individuals and assessing the consistency of the underlying protein profiles. Across 1000 iterations, the model achieved a median classification accuracy of 0.98 (Q_1; 25%_ = 0.961 & Q_3; 75%_ = 1). This indicates that, when one sample per donor is withheld and blinded from the training set, the model correctly assigns the sample to the corresponding donor in median 98% of cases.

**Figure EV2:**
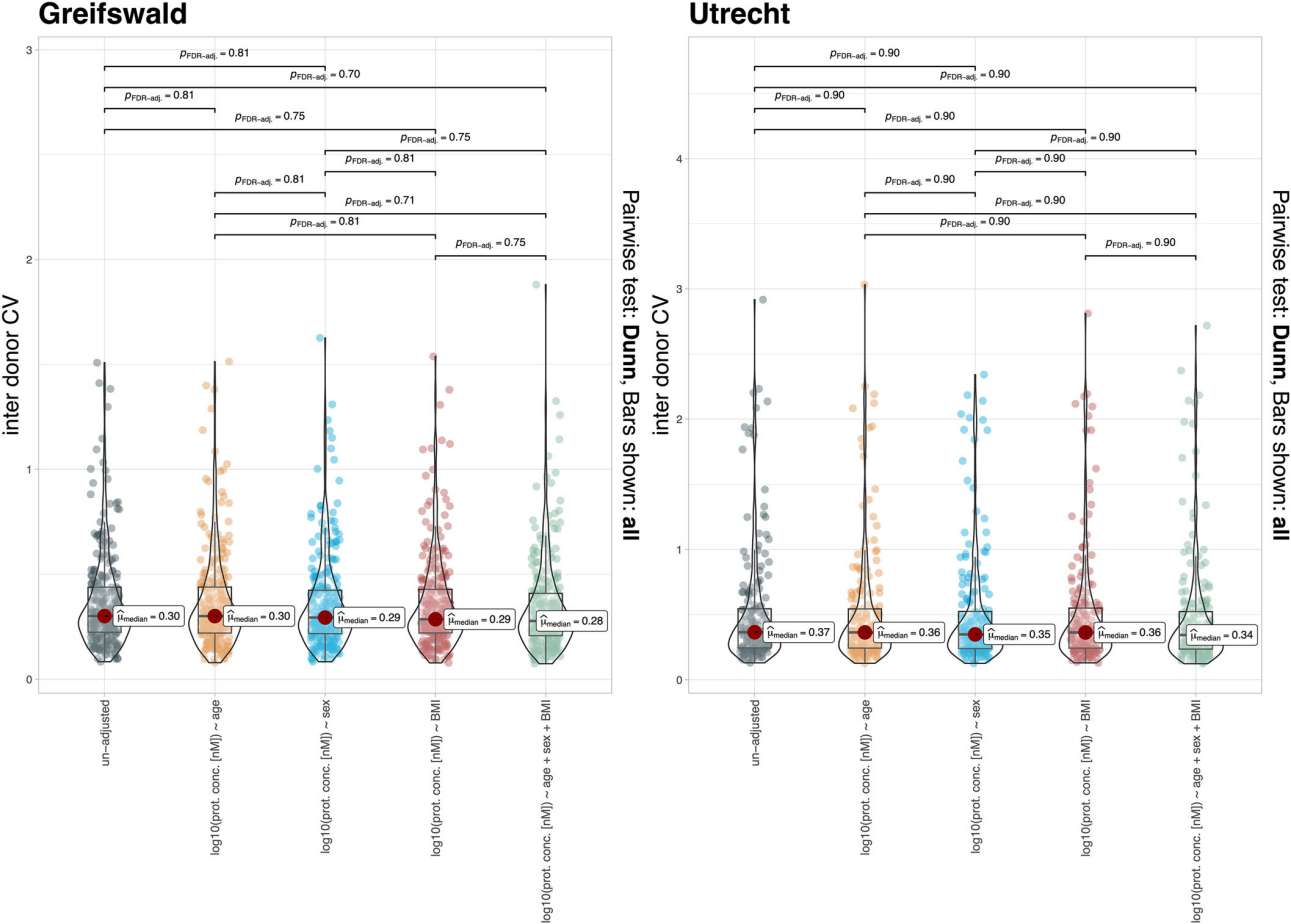
Violin-Boxplot comparing the inter-donor coefficient of variation (CV) between confounder-adjusted concentration data of Greifswald site (left panel, *n* = 274 proteins per model) and Utrecht site (right panel, *n* = 188 proteins per model). The x-axis shows the separate linear regression models for data adjustment, while the y-axis represents the inter-donor CV. The median CV for each model and lab site is highlighted, and its value is displayed. Additionally, the Dunn tests adjusted p-values for pairwise comparisons are presented. Box plots display the following statistical descriptors: center line, median; box bounds, 25th and 75th percentiles (interquartile range, IQR); whiskers, 1.5 × IQR below the 25th and above the 75th percentile; points beyond the whiskers represent outliers; minima and maxima indicate the smallest and largest observed values within the whisker range, respectively.

**Figure EV3:**
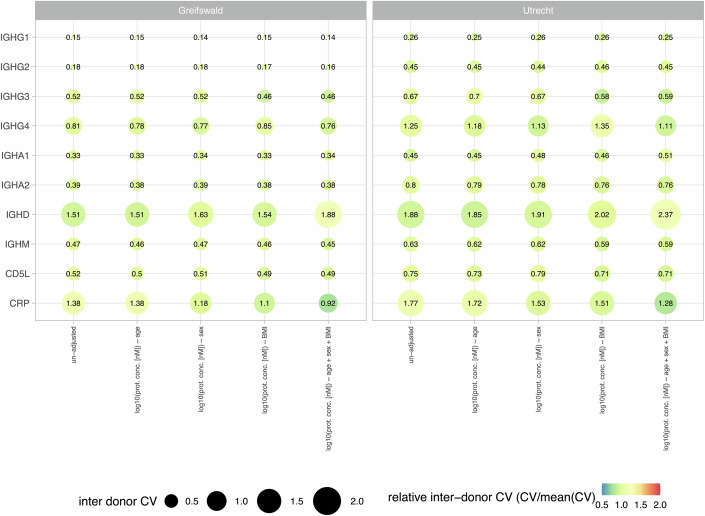
Inter donor coefficient of variation (CV) plots for selected proteins, along with separate linear regression models. The left panel represents the data from the Greifswald site, whereas the right panel represents the data from the Utrecht site. The size of the points represents the inter-donor CV, while the color of the points indicates the scaled relative per protein and site inter-donor CV. Additionally, the inter-donor CV is also displayed as a rounded number with two digits.

To evaluate the impact of confounders like age, sex, and BMI on inter-donor variability, linear regression modeling for each protein group at each study site was carried out to adjust the protein concentration data. Inter-donor CVs were calculated for the unadjusted data and each confounder-adjusted concentration dataset. The results revealed (Fig. EV2) no significant differences in inter-donor variability for any adjustment model, suggesting that age, sex, and BMI do not substantially drive the observed inter-donor differences in protein concentration in this small cohort. Furthermore, the inter-donor CV per protein remains consistent across different sites (Fig. EV3). In general, slightly lower inter-donor CVs were observed with the timsTOF Pro2 data at the Greifswald site. The timsTOF HT features a fourth-generation TIMS-XR analyzer with a fivefold increase in ion charge capacity and a fourfold improvement in vertical dynamic range compared to the timsTOF Pro2. These hardware differences result in distinct quantitative behaviors: the Pro2 exhibits TIMS cartridge saturation, while the HT maintains linear quantitative response (Vitko et al, 2024). This leads to peptide abundance-specific quenching effects in the Pro2, causing identification drops and different MS2 peak area distributions for the same samples. This effect is evident in the inter-donor variability when comparing highly abundant plasma proteins such as albumin or transferrin (Appendix Figs. S4 and S5). The timsTOF Pro2 data (Greifswald site) showed an inter-donor CV of 0.12 for albumin, while the timsTOF HT (Utrecht site) data revealed an inter-donor CV of 0.26. The same trend is observed for transferrin, with an inter-donor CV of 0.08 for the timsTOF Pro2 data and 0.24 for the timsTOF HT data. Immunoglobulins constitute a dominant class of plasma proteins, accounting for ~20% of the total protein mass in human blood. Among these, IgG, IgA, and IgM are the most prevalent, with IgG typically representing the most abundant subclass. Human IgG is further subdivided into four structurally related subclasses—IgG1, IgG2, IgG3, and IgG4—each with distinct sequence motifs, structural properties, and immunological functions. The subclass nomenclature of IgG has been traditionally based on their relative mean concentrations in circulation. Within each subclass exists a heterogeneous repertoire of immunoglobulin clones, ranging from thousands to millions of unique variants, differing subtly in their variable region sequences (Bondt et al, 2021). This molecular diversity poses significant analytical challenges for plasma proteomic studies, particularly in database curation and peptide identification workflows. Consequently, the immunoglobulin data are frequently excluded or actively depleted in most reported plasma proteome studies to date, likely to reduce complexity and enhance detection of lower-abundance proteins.

**Figure 4:**
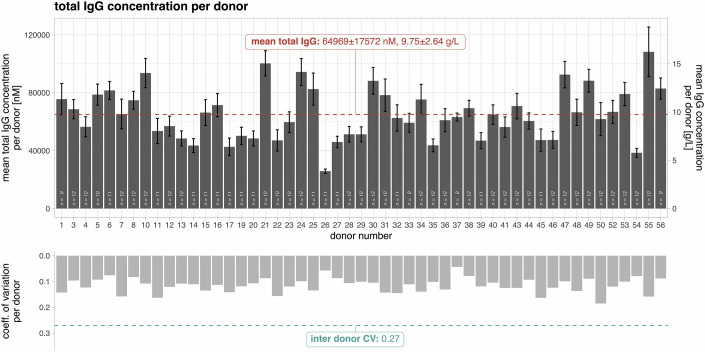
Total IgG profiles reveal pronounced inter-donor variability. Total IgG concentrations were derived by summing all measured IgG subclasses (IgG1–IgG4) for each donor at every sampling time point, yielding cumulative IgG levels in nanomolar (nM) units. The resulting bar plot depicts the mean and standard deviation of these total IgG concentrations across time points for each individual, with the number of contributing time point measurements per donor shown for each individual. Corresponding values in grams per liter (g/L) were computed from nM concentrations using molecular weight–based conversion. The inter-donor coefficient of variation (CV) is depicted as a dashed turquoise line, and its exact value is provided in the label. Similar data presentations are shown in Appendix Figs. S7, S8, S14, S15, but then categorized by immunoglobulin subclasses.

**Figure EV4:**
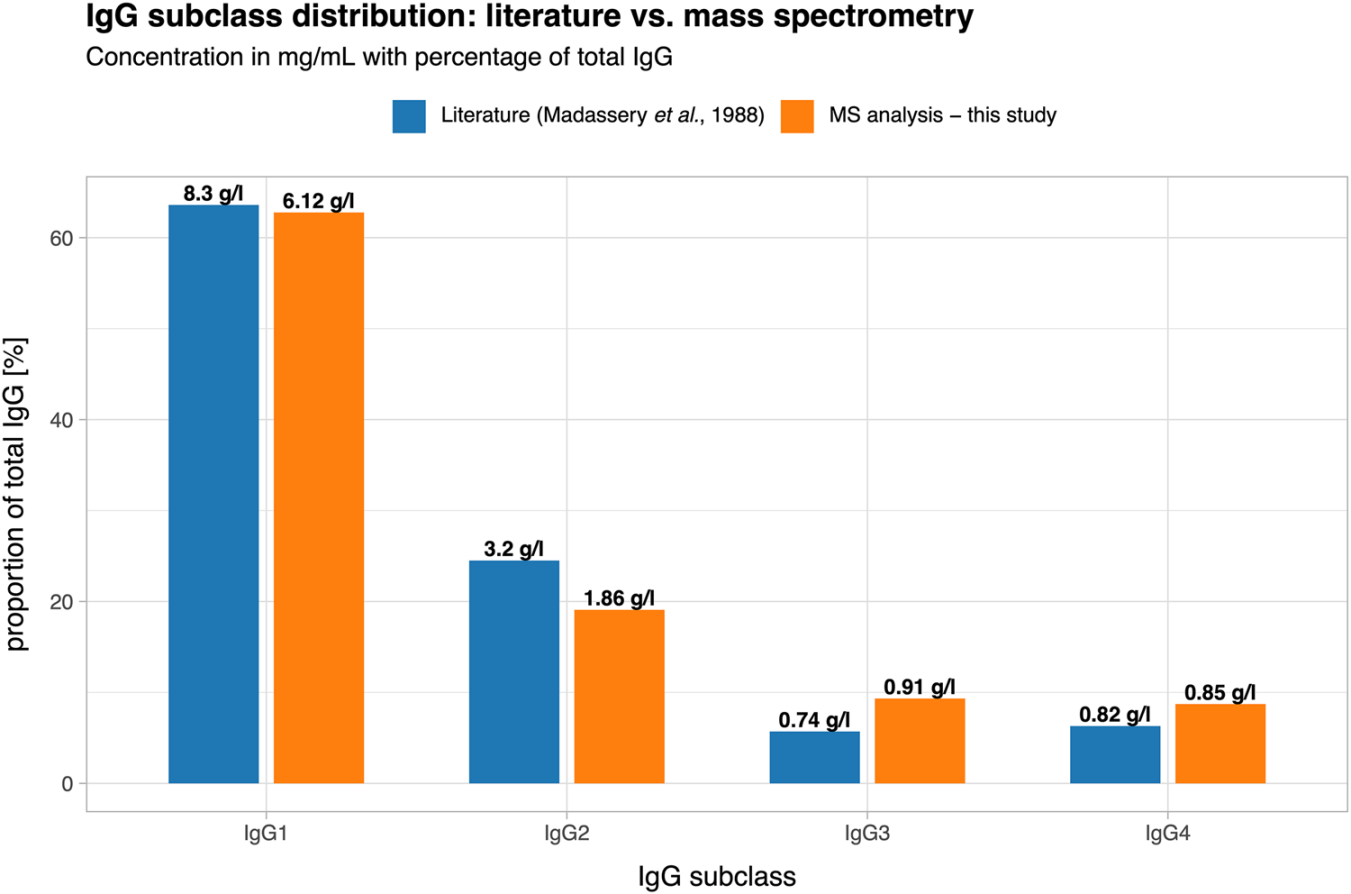
Bar plot depicts the proportion of IgG subclasses known from literature (blue bars) and measured in the TIMES cohort via mass spectrometry analysis (orange bars).

In contrast, our analysis retained the immunoglobulin pool, allowing for quantitation and comparative assessment of these proteins across donors. This approach provides novel insights into the inter-individual variance and underscores the biological relevance of immunoglobulin dynamics in longitudinal plasma proteomics. Despite notable sequence homology among immunoglobulin classes, each exhibits unique peptide sequences, particularly within their Fc domains. These unique regions allow confident identification and semi-quantitative analysis using tryptic peptide profiling. Utilizing peptides unique to each subclass that correspond to the constant heavy chains of IgG1 through IgG4, we were able to estimate the total IgG concentration by assessing the concentration of each subclass and to monitor the temporal consistency of these concentrations. As illustrated in Figs. 4 and EV4, the mass spectrometry-derived concentration estimates showed a strong concordance with established plasma reference ranges for IgG (6.20–15.10 g/L). Across the cohort, the average total IgG concentration across donors was 9.75 g/L, with a standard deviation of 2.64 g/L (27%), and ranged from 3.87 to 16.24 g/L. Strikingly, while inter-donor variability remained substantial (SD over mean total IgG per donor = 2.64 g/L, CV = 0.27, IQR_95%–5%_ = 7.64 g/L), intra-donor fluctuations were minimal (mean SD = 1.11 g/L, mean CV = 0.115, IQR_95%–5%_ = 3.04 g/L), suggesting longitudinal stability within individuals over the 1-year sampling period.

**Figure 5:**
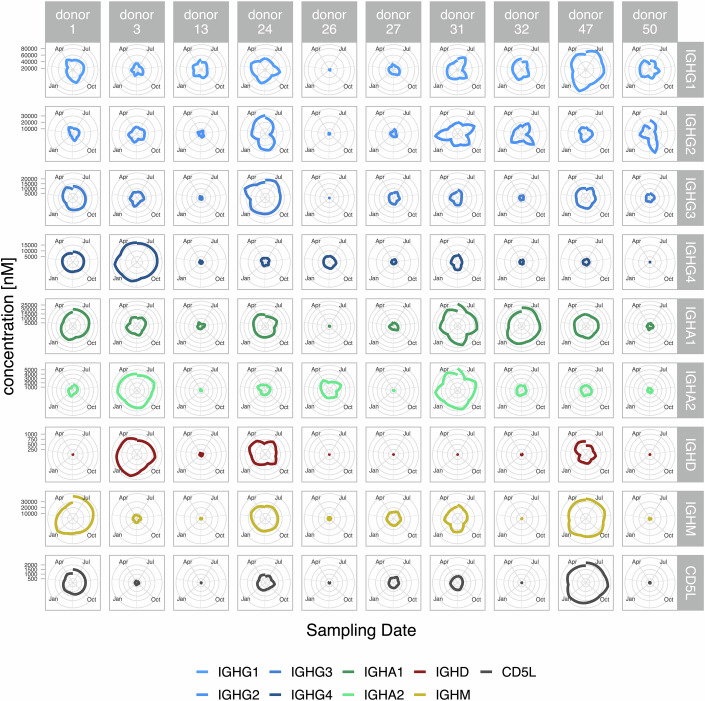
Immunoglobulin subclasses and CD5L intra- and inter-donor variability over time. Nanomolar (nM) concentrations of immunoglobulin subclasses and CD5L are visualized per donor using radar plots. In this representation, a smaller circular diameter indicates a lower relative concentration compared to other donors. The closer the plotted line approximates a circle, the more stable the concentration remains over the 12-month observation period. Of note, as depicted in this figure, donor 26 is characterized by overall low levels of nearly all classes of monitored Ig’s (with IgA2 being the exception). These radar plot representations also illustrate nicely the huge inter-donor variation in specific Ig levels. To illustrate for IgD, donors 3, 24 and 47 have relatively high levels of IgD, whereas in the other donors, this Ig class is barely detectable. The patterns of Ig subclass levels in plasma are highly individual. There is little correlation between them except for IgM and the associated protein CD5L.

Similarly, the four IgG subclasses showed high inter-individual variability, but only minor intra-donor fluctuations (Fig. 5; Appendix Figs. S6–S15), reinforcing the notion that immunoglobulin levels are tightly regulated under homeostatic conditions. This pattern was particularly pronounced in IgG3, IgG4, IgM, IgA2, and IgD. Besides, our data revealed a distributional pattern of CD5L that closely mirrored that of IgM, consistent with prior evidence identifying CD5L as an integral and ubiquitous constituent of plasma IgM complexes (Oskam et al, 2023; Wang et al, 2024). The observed stability in the relatively small study likely confirms that the donors were probably healthy. Also, these findings could be validated fully in the data obtained in parallel at the Greifswald site.

Since immunoglobulins are frequently excluded from contemporary proteomics studies, we benchmarked our data against results from an ELISA-based analysis of 600 healthy donors (Gonzalez-Quintela et al, 2008). That study reported an average total IgG concentration of 11.1 g/L with a standard deviation of 2.5 g/L (25%), ranging from 7.0 to 17.4 g/L, whereas another study reported a range of 6.20–15.10 g/L (Khan et al, 2021). Literature-based adult Ig subclass distributions (Madassery et al, 1988; Schauer et al, 2003) suggest approximate concentrations of IgG1: 8.3 mg/mL (~63.6% of total IgG), IgG2: 3.2 mg/mL (~24.5% of total IgG), IgG3: 0.74 mg/mL (~5.7% of total IgG), and IgG4: 0.82 mg/mL (~6.3% of total IgG). In comparison, our mass spectrometry-based quantification yields an average IgG1 at 6.12 mg/mL (62.77% of total IgG), IgG2 at 1.86 mg/mL (19.08%), IgG3 at 0.91 mg/mL (9.33%), and IgG4 at 0.85 mg/mL (8.72%) (Fig. EV4). Intriguingly, and discussed later, this data also revealed that immunoglobulin levels of donor 26 were consistently 4–10-fold lower than the average, for IgG1, IgG2, IgG3, IgA1, and IgM, i.e, all below the 2.5th percentile.

**Figure 6:**
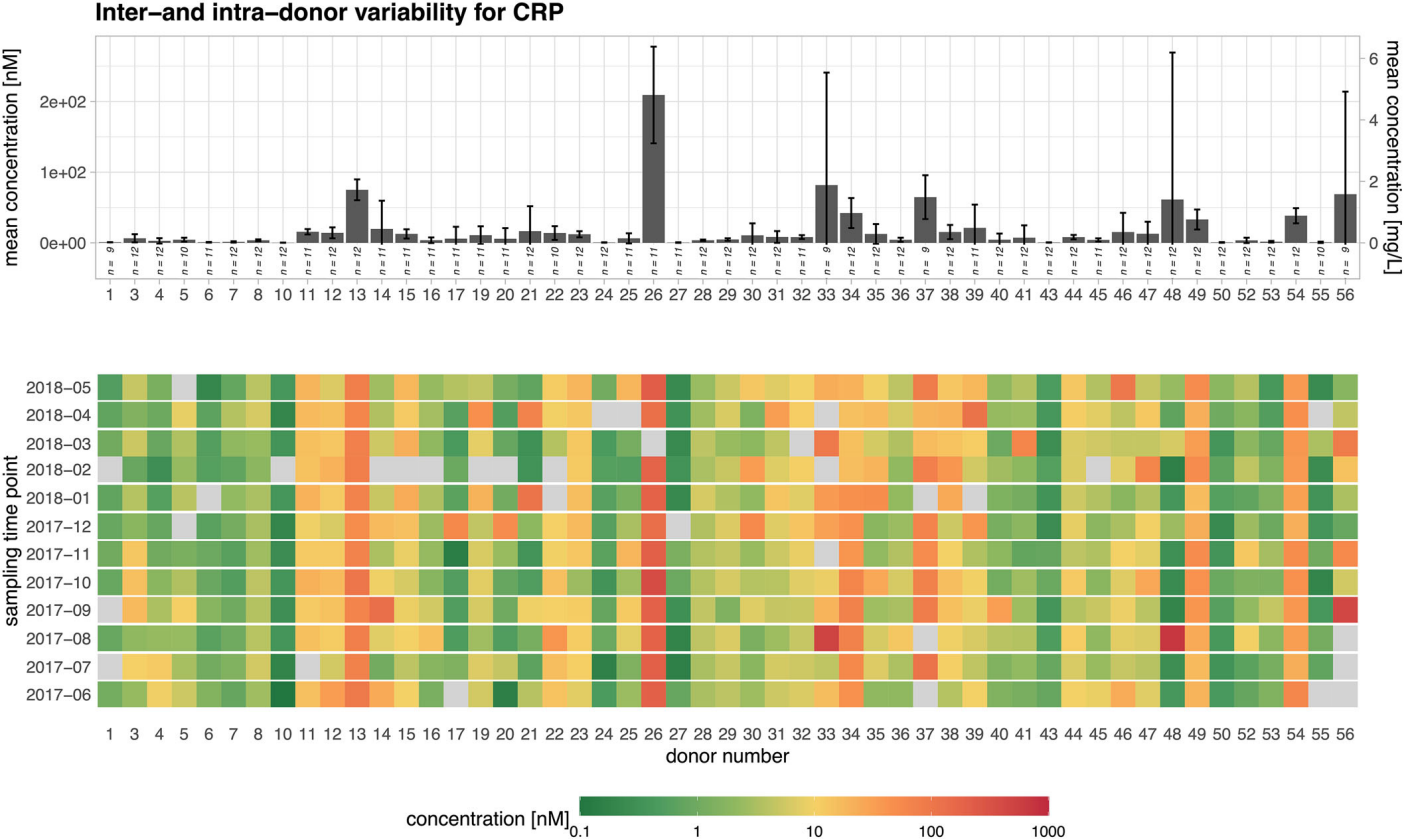
Intra- and inter-donor variability of CRP. CRP mean concentration shown in bar plot (top panel), with the number of contributing time point measurements per donor shown for each individual. The tile plot below (lower panel) depicts the temporal profiles of CRP in donors, whereby donor 13, 26, 34, 37, 49, and 54 display continuous basal elevated levels of CRP (compared to the average) and donors 21, 33, 48, and 56 show across the year relative low levels of CRP, with temporal bursts of concentration increases at single sampling points. The error bars are the mean value ± standard deviation.

A major observation in our study is that most proteins display very little change in plasma concentration when monitored at regular intervals over the time span of a year. There are, however, several notable exceptions, exemplified by well-known inflammation markers, such as C-reactive protein (CRP), SAA1/SAA2, and S100A8 and S100A9. For instance, CRP concentrations were generally quite low but exhibited pronounced temporal increases, albeit only in a few donors (Fig. 6), resulting in substantial average differences between donors. The average total CRP concentration across donors was 19.25 nM, with a standard deviation of 34 nM (176%), and ranged from 0.28 to 209.16 nM (~750x increase). The annual intra-donor fluctuations were minimal from 0.10–0.39 nM (donor 10) to a maximal 0.16–721.43 nM (donor 48) (Fig. 6). Of interest, a few donors (13, 26, 34, 37, 49, and 54) revealed a relatively high basal CRP concentration of ~50 nM across all longitudinal sampling points. In contrast, donors 21, 33, 48, and 56 had across the year rather low levels of CRP ( < 5 nM), but at discrete sampling points the CRP concentrations temporally increased to some of the highest levels measured (i.e., >200 nM). We hypothesize that such spikes in the CRP concentration could be linked to short-term events of acute inflammation. Additionally, the inter- and intra-variation patterns observed for CRP across all donors and time points, were mirrored by the functionally related inflammation markers SAA1, SAA2, and S100A8 and S100A9 (Appendix Figs. S16–S20). The consistency in these episodic peaks observed across all the inflammation markers, strengthens the notion of acute inflammation episodes. Again, also these observations on inflammation markers were very similar in the datasets obtained in Utrecht and Greifswald (Appendix Figs. S16–S20).

## Discussion

### Cross-site reproducibility and platform correlation

A major finding, extracted from our collaborative work is that despite substantial differences in sample digestion protocols, injection amounts, instrument dynamic ranges (timsTOF Pro2 vs. timsTOF HT), and data processing workflows, strong correlation was observed between the datasets acquired at the two sites, even with substantially differing protein identification counts. This correlation has potential for further improvement in the timsTOF Pro2 dataset. Vitko et al (Vitko et al, 2024) directly compared the timsTOF Pro2 and timsTOF HT under varying loading amounts and LC gradient conditions. In neat plasma analyses, the timsTOF HT preserved peptide identification rates across increasing sample loads, whereas the timsTOF Pro2 exhibited marked losses, particularly with short LC gradients at >300 ng relative to 100 ng. Quantitative assessment demonstrated MS2 signal attenuation with increasing load on both platforms, with a more pronounced effect for the timsTOF Pro2. At ≥300 ng, the timsTOF HT yielded narrower signal intensity distributions and lower coefficients of variation (CVs), indicating enhanced reproducibility. These results indicate that a sample loading greater than 200 ng is expected to yield a protein identification count comparable to that obtained with the timsTOF Pro2 at 300 ng using the 60 SPD LC workflow. Despite the different mass spectrometers used in our study in combination with different sample loading amounts and at the same time using the same LC setup and gradient, the workflow also varied regarding sample preparation as one site used SP3 sample digest (Hughes et al, 2019) whereas the other used a liquid digest approach. SP3 uses paramagnetic beads for protein capture, allowing removal of detergents and other contaminants through multiple wash steps before on-bead digestion. This approach generally improves peptide recovery from low-input samples, enhances reproducibility, and minimizes sample losses during transfer (Hughes et al, 2019). In contrast, conventional liquid digestion (in-solution digestion without bead capture) relies on precipitation or dilution to remove detergents, which can lead to variable recovery and reduced reproducibility, particularly at low protein amounts. Liquid digest methods are simpler in workflow but more susceptible to incomplete detergent removal and peptide loss during cleanup. SP3 and SDC in solution digest methods achieved broadly comparable overall proteome coverage; large overlaps have been seen across various bottom-up methods generally (Varnavides et al, 2022). However, SP3-derived samples displayed distinct patterns regardless of the applied buffer systems, suggesting that magnetic bead–mediated protein pulldown constitutes a major determinant of method-specific protein profiles (Varnavides et al, 2022). Altogether, these observations indicate that normalization of the data by protein is required to enable valid comparisons between workflows. Although averaging across proteins and adjusting the mean values could facilitate such comparisons, the inter-laboratory evaluation results strongly support including a universal plasma reference sample or using spike-in isotopically labeled internal standards with each measurement workflow (Cai et al, 2025) since cohorts and disease phenotype plasma proteome can strongly vary. This approach would allow adjustment of protein intensities independent of the sample preparation, mass spectrometry device and analysis workflow, enabling comparability across cohorts, ensuring standardization and reproducibility in multi-center studies in the future.

### Intra- and inter-individual plasma proteome variability

The longitudinal study design of the TIMES cohort revealed that inter-individual differences in plasma protein concentrations largely exceed intra-individual variability over time per protein. Complementary findings from a longitudinal study of healthy Danish adults demonstrated that total IgG concentrations, along with most IgG subclasses, remained remarkably stable within individuals over a period of at least 42 weeks (Rasmussen et al, 2021). These results underscore the robustness of individual immunoglobulin profiles and support their potential utility in longitudinal biomarker studies and personalized health monitoring (Rasmussen et al, 2021). These findings also suggest that a single measurement of IgG subclass levels is generally sufficient to represent an individual’s immunoglobulin profile, even across seasons.

Recently, van Rijswijck et al (Rijswijck et al, 2025) reported that the majority of very highly abundant antibodies in the plasma are generated by long-lived plasma cells. They showed that usually the production and secretion levels (and thus plasma concentration levels) of these highly abundant antibodies were very stable over time. The stability of the dominant immunoglobulin clones could explain why we also observe very little intra-donor variability in immunoglobulin levels in this study.

But, although the individual proteome remains relatively stable over time, substantial variability exists between donors. This longitudinal cohort also highlights the donor-dependent baseline difference in some proteins of clinical relevance, such as CRP. On the one hand, such inter-individual heterogeneity may be clinically meaningful, indicating, for example, low-grade chronic inflammation. Furthermore, pronounced differences in individual baseline values might obscure the biological signals of acute events in clinical studies, if not properly accounted for. To mitigate this, it would be essential for clinical proteomic analyses to incorporate donor- or patient-specific control samples and express results as relative ratios. Such normalization strategies help adjust for baseline differences and enhance the sensitivity, accuracy and interpretability of biomarker discovery and disease profiling.

### Conclusion

Our study underscores the potential of longitudinal sampling in plasma proteomics, providing deep insights into both intra- and inter-individual variations over time. By leveraging independent mass spectrometry workflows and robust data analysis pipelines, we highlighted the challenges and solutions in cross-laboratory data harmonization. The observed stability of immunoglobulin subclasses contrasts starkly with the dynamic fluctuations of proteins like CRP, reflecting the complexity of human plasma proteomics and interpretation of the results. These findings stress the importance of longitudinal designs for accurately capturing the dynamic biological landscape, which is critical for biomarker discovery research and clinical applications.

### Limitations of the study

Despite the robust design and comprehensive data generated in this study, several limitations need to be acknowledged. First, the cohort size, although sufficient for detecting intra- and inter-individual variations, may limit the generalizability of the findings to broader populations with different demographic or health profiles. Further longitudinal studies with larger, more diverse cohorts are needed to validate and extend these findings.

Secondly, although the TIMES cohort was designed to recruit healthy donors, there is no independent clinical evidence for the absence of disease in all participants. For example, donor 26 is an outlier in terms of high BMI and body fat proportion (Fig. EV1), very high basal levels of C-reactive protein (Fig. 6) over the whole year, and very low levels of nearly all immunoglobulin subclasses, namely IgG1, IgG2, IgG3, IgA1, and IgM (see Fig. 5; Appendix Figs. S7, S9, S12, S14, S15). Clinically, this is suggestive of common variable immunodeficiency (CVID), the most prevalent human primary immunodeficiency. The disease is usually diagnosed at around 30 years of age because of frequent and severe infections (Bonilla et al, 2015). However, donor 26 did not report increased susceptibility to infections. We conclude that her abnormally low immunoglobulin plasma levels might reflect immunodeficiency requiring further diagnosis. This chance finding highlights the potential value of diagnostic plasma proteomics.

Another limitation of the cohort may be that it does not include children/young adults and old people, but inter- and intra-donor plasma protein variability may be affected by age. It is well known that immunoglobulin concentrations are gradually built up during childhood and adolescence (Rich et al, 2018). In the elderly, on the other hand, the prevalence of subclinical and clinical health disorders increases. Thus, our conclusions mostly relate to the age group studied, namely, between 19 and 56 years.

Moreover, we here focus solely on the ~200 plasma proteins that are consistently detected and can be robustly quantified over all donors and all time points. It has been shown that with more efforts, the detectable plasma proteome can be increased to over 1000 proteins, where the gain comes mostly from lower-abundant proteins. However, we did not aim for that as it would substantially increase the number of missing values, and thus also CVs and robustness in quantification. However, our study focuses largely on immunoglobulins, which are highly abundant plasma proteins. In the published large-scale in-depth plasma proteomics studies the immunoglobulins are often depleted from the plasma samples in order to reach a deeper depth in the proteome, whereas when not depleted they are often ignored in the subsequent data analysis, because of their extreme sequence variability.

## Methods

**Table:** Reagents and tools table

Reagent/resource	Reference or source	Identifier or catalog number
**Chemicals, enzymes and other reagents**		
2-Chloroacetamide	Sigma-Aldrich	Cat# C0267
4-(2-hydroxyethyl)-1-piperazineethanesulfonic acid)(HEPES)	Roth	Cat# 9105.3
Acetic acid	Roth	Cat# 3738.5
Acetontril	Thermo Scientific	Cat# 047138.M1
Dithiothreitol (DTT)	Thermo Scientific	Cat# A39255
Formic acid 0.1% in Acetonitrile ULC/MS	Biosolve	Cat# 0001934102BS
Formic acid 0.1% in Water ULC/MS	Biosolve	Cat# 0023244102BS
HPLC-grade water	JT Baker	Cat# 4218.2500
Iodoacetamide (IAA)	Thermo Scientific	Cat# A39271
Lysyl Endopeptidase	Wako	Cat# 129-02541
Micro BCA™ Protein-Assay-Kit (500 ml)	Thermo Scientific	Cat# 23235
Sera-Mag SpeedBeads Carboxylate (hydrophobic)	Cytiva	Cat# 65152105050250
Sera-Mag SpeedBeads Carboxylate (hydrophylic)	Cytiva	Cat# 45152105050250
Sodium deoxycholate (SDC)	Sigma-Aldrich	Cat# 30970
Sodium dodecyl sulfate (SDS) ultra pure	Roth	Cat# 2326.3
Trifluoroacetic acid	Sigma-Aldrich	Cat# T6508
Trifluoroacetic acid	Thermo Scientific	Cat# 13464279
Tris(2-carboxyethyl)phosphine hydrochloride	Sigma-Aldrich	Cat# C4706
Trizma® Pre-set crystals (Tris)	Sigma-Aldrich	Cat# T7943
Trypsin from bovine pancreas	Sigma-Aldrich	Cat# T1426
Trypsin/LysC Mix, Mass Spec Grade	Promega	Cat# V5073
**Software**		
Spectronaut version 20.1	https://biognosys.com/software/spectronaut/	
DIA-NN v1.9	https://github.com/vdemichev/DiaNN	
R version 4.4.1	https://cran.r-project.org	
R package: broom (version: 1.0.12)	https://CRAN.R-project.org/package=broom	
R package: dendextend (version: 1.19.1)	10.1093/bioinformatics/btv428	
R package: factoextra (version: 2.0.0)	https://CRAN.R-project.org/package=factoextra	
R package: FactoMineR (version: 2.13)	https://CRAN.R-project.org/package=factoMineR	
R package: furrr (version: 0.3.1)	https://CRAN.R-project.org/package=furrr	
R package: fuzzyjoin (version: 0.1.8)	https://CRAN.R-project.org/package=fuzzyjoin	
R package: ggh4x (version: 0.3.1)	https://CRAN.R-project.org/package=ggh4x	
R package: gghalves (version: 0.1.4)	https://github.com/erocoar/gghalves	
R package: ggrepel (version: 0.9.8)	https://CRAN.R-project.org/package=ggrepel	
R package: ggsignif (version: 0.6.4)	https://psyarxiv.com/7awm6	
R package: ggstatsplot (version: 0.13.3)	10.21105/joss.03167	
R package: ggtext (version: 0.1.2)	https://CRAN.R-project.org/package=ggtext	
R package: glue (version: 1.8.0)	https://CRAN.R-project.org/package=glue	
R package: helfRlein (version: 1.5.0)	https://github.com/STATWORX/helfRlein	
R package: kernlab (version: 0.9.33)	https://CRAN.R-project.org/package=kernlab	
R package: paletteer (version: 1.7.0)	https://github.com/EmilHvitfeldt/paletteer	
R package: patchwork (version: 1.3.2)	https://CRAN.R-project.org/package=patchwork	
R package: psych (version: 2.6.3)	https://CRAN.R-project.org/package=psych	
R package: purrr (version: 1.2.1)	https://CRAN.R-project.org/package=purrr	
R package: RColorBrewer (version: 1.1.3)	https://CRAN.R-project.org/package=RColorBrewer	
R package: readxl (version: 1.4.5)	https://CRAN.R-project.org/package=readxl	
R package: rsample (version: 1.3.2)	https://CRAN.R-project.org/package=rsample	
R package: scales (version: 1.4.0)	https://CRAN.R-project.org/package=scales	
R package: SpectroPipeR (version: 0.4.6.9001)	https://github.com/stemicha/SpectroPipeR	
R package: tidymodels (version: 1.4.1)	https://www.tidymodels.org	
R package: tidyverse (version: 2.0.0)	https://CRAN.R-project.org/package=tidyverse	
R package: umap (version: 0.2.10.0)	https://CRAN.R-project.org/package=umap	
R package: yardstick (version: 1.3.2)	https://CRAN.R-project.org/package=yardstick	
**Other**		
1.7 ml Low Binding MCT	Sorenson BioScience	Cat# 39640 T
250 uL Tips, 96 Tips/Rack, for Bravo LT Head	Agilent	Cat# 19477
Evotip Pure	EVOSEP	Cat# EV2011
Greiner 650201, 96-well plates, polypropylene	Greiner Bio-One	Cat# M7310
Hard-Shell® 96-Well PCR Plate, skirted	Bio-Rad	Cat# HSP9611
MASTERBLOCK® 96w, 1 ml, U-bodem, deep, PP	Greiner Bio-One	Cat# 780201
NEST 0.2 mL 96-Well PCR Plate, Full Skirt	Opentrons	Cat# 999-00050
Reservoir Microplate, 8 row, polypropylene, 32 mL/row	Agilent	Cat# 201260

### Study design and participants

The TIMES study aimed to investigate the circannual fluctuations in the plasma proteome of healthy donors over a period of 12 months. Inclusion criteria included (i) age 18–60 years, (ii) written informed consent, and (iii) no acute illness in the last seven days before sampling. Exclusion criteria included (i) body mass index <18.5 kg/m^2^, and (ii) presence of blood coagulation disorders, anemia or similar conditions. Plasma samples were obtained from a total of 53 participants once per month for 1 year (June 2017–May 2018). Two subjects were excluded from data analyses due to early loss to follow-up. Data from the remaining 51 subjects were included in the analyses (Appendix Table S2; Fig. EV1).

### Ethics and data protection

The TIMES study was approved by the Ethics Committee of the University Medicine Greifswald (internal registration numbers BB 043/17 and BB 001/21 f). All work was conducted in accordance with the tenets of the Declaration of Helsinki (version 2013, Fortaleza). All requirements of data protection and confidentiality according to local regulations, the State Data Protection Act, Mecklenburg-Western Pomerania, the European Data Protection Directive 95/46/EC, and the General Data Protection Regulation (GDPR) were fully met.

### Plasma sample collection

Blood samples were collected using EDTA-Vacutainer (BD, K2E RAF 368861) tubes, with BD Vacutainer Safety Lock needles (REF 367282) facilitating the draw. Post-collection, the samples were maintained at room temperature for a maximum of one hour prior to centrifugation. Centrifugation parameters were meticulously controlled, with plasma samples subjected to 2000 × *g* for 10 min at room temperature. Plasma extraction was performed carefully, leaving ~0.5 cm of plasma above the PBMC layer to maintain sample integrity. All aliquots were promptly stored at −70 °C. The blood donations took place at the University Medicine Greifswald (UMG) between 7:30 and 9:00 a.m., and aliquots were frozen by 11:00 a.m. at the latest. Samples were processed concurrently with ongoing collection, with batches of 24 samples transported to the laboratory for immediate mixing and centrifugation, ensuring a maximum pre-centrifugation delay of one hour. Samples were stored at −70 °C. After thawing and aliquoting, they were frozen and shipped on dry ice to the Utrecht laboratory site. The samples were prepared and measured in Utrecht and Greifswald during the same time period.

### Plasma proteomics–Utrecht site

The plasma proteomics workflow was adapted from previous work (Völlmy et al, 2021; Nteak et al, 2024) and performed in five batches with two 96-well plates in each batch. Each plate included four to six QC samples of pooled serum from 400 healthy donors (HSER-CUSTOM, amsbio) to monitor any batch effects. Samples were processed using nested randomization by first randomizing the order of time points for each individual donor and then randomizing the sequence of the resulting donor blocks. Consequently, donors were measured in a random order, with their respective time points appearing as a randomized cluster. Randomized sample order was the same for the Utrecht and Greifswald sites. Twenty-two µL of plasma samples and QC samples were subjected to a 20-fold dilution using 100 mM Tris buffer at pH 8.5. Subsequently, 40 µL of the diluted samples were transferred into 20 µL of Buffer 1, comprising of 4% sodium deoxycholate, 160 mM chloroacetamide, and 100 mM Tris at pH 8.5, followed by the addition of 20 µL Buffer 2, containing 40 mM tris-(2-carboxyethyl)-phosphine in 100 mM Tris at pH 8.5. The mixture was incubated at 95 °C for 10 min to facilitate denaturation and reduction/alkylation processes. Post-incubation, 100 µL of 100 mM Tris buffer at pH 8.5 was added to dilute the mixture, followed by the addition of 10 µL each of Trypsin (Sigma-Aldrich) and LysC (Wako), both at a stock concentration of 0.2 µg/µL. The samples were then incubated overnight at 37 °C to ensure complete digestion. Enzymatic activity was quenched with the addition of 200 µL of 2% trifluoroacetic acid (TFA) and centrifuged to separate the precipitated TCEP. The resulting digest was further diluted 36-fold in 1% TFA, yielding a final concentration of ~10 ng/µL (based on an average plasma protein concentration of 70 µg/µL). Twenty µL of roughly 200 ng of total protein were loaded onto Evosep tips following the manufacturer’s instructions. The samples were analyzed using the 60 SPD method on an Evosep ONE LC (Evosep, Odense, Denmark) with an EV-1109 analytical column (ReproSil Saphir C18, 1.5-μm beads by Dr. Maisch; 8 cm length × 150 μm i.d.; Evosep). Gradient elution was achieved using mobile phases A (0.1% HCOOH in Milli-Q water) and B (0.1% HCOOH in 100% CH_3_CN). The LC was coupled to a timsTOF HT mass spectrometer (Bruker Daltonics, Bremen, Germany). Peptide ionization was ensured using a captive source equipped with a ZDV sprayer (20 µm ID, 1865691, Bruker Daltonics). The sprayer was operated at a spray voltage of 1600 V, with drying gas flowing at 3 L/min and drying at 180 °C. The dia-PASEF-MS mode was configured for MS and ion mobility windows, as described in Appendix Table S4.

Peptides were isolated within the m/z range of 300–1200, with 12 dia-PASEF scans and 8.3% duty cycle. The IM range was set to 1.6 and 0.6 V cm^2^, and the accumulation and ramp times were set to 100 ms. Collision energy ramping was performed according to the manufacturer’s default settings (20–59 eV within the mobility range of 0.6–1.6 Vs/cm^2^). Acquisition was conducted with a ramp and accumulation time of 100 ms, achieving a 100% duty cycle.

### Plasma proteomics–Greifswald site

The plasma samples were diluted at a ratio of 1:50 in a solution comprising 50 mM HEPES buffer at pH 8, supplemented with 1% sodium dodecyl sulfate (SDS) to ensure optimal protein solubilization. Subsequently, the diluted samples underwent thermal denaturation at 95 °C for a duration of 5 min. Upon returning to ambient temperature, the samples were processed through the OT-2- assisted MassSpecPreppy workflow (Reder et al, 2023), enabling precise protein concentration determination and enzymatic digestion. Three µg of total protein was reduced with dithiothreitol (DTT) and alkylated using iodoacetamide (IAA). The enzymatic digestion was conducted at an enzyme-to-protein ratio of 1:25. To stop the proteolytic digestion, after at least 16 h 2.44 µl of 5% (v/v) TFA was added to the peptide mixture. The final preparation entailed the loading of 300 ng of the digested material onto EvoTips for subsequent mass spectrometric analysis. Each microtiter plate contained three to four batch standard plasma samples (pool of healthy donors) alongside donor samples to monitor batch effects. Samples were processed using nested randomization by first randomizing the order of time points for each individual donor and then randomizing the sequence of the resulting donor blocks. Consequently, donors were measured in a random order, with their respective time points appearing as a randomized cluster. Randomized sample order was the same for the Utrecht and Greifswald sites. The samples were measured with a 60 SPD method using an Evosep performance column (EV-1109, 8 cm × 150 µm × 1.5 µm) on an Evosep ONE LC (Evosep, Odense, Denmark) coupled to a timsTOF Pro2 (Bruker Daltonics, Bremen, Germany). Peptide ionization was ensured using a captive source equipped with a ZDV sprayer (20 µm ID, 1865710, Bruker Daltonics, Bremen, Germany). The sprayer was operated at a spray voltage of 1640–1740 V, with drying gas flowing at 3 L/min and drying at 180 °C. The dia-PASEF-MS mode was configured for MS and ion mobility windows, as described in Appendix Table S3.

Peptides were isolated within the m/z range of 330.17-1638.48, comprising 16 m/z windows with two ion mobility steps and eight dia-PASEF scans each. These scans were performed within the ion mobility range of 0.57–1.47 Vs/cm^2^, resulting in a cycle time of 0.95 s. Collision energy ramping was performed according to the manufacturer’s default settings (20–52 eV within the mobility range of 0.6–1.6 Vs/cm^2^). Acquisition was conducted with a ramp and accumulation time of 100 ms, achieving a 100% duty cycle.

### Raw data analysis–Utrecht site

Raw data analysis was conducted using DIA-NN 1.9 (Demichev et al, 2020). Carbamidomethylation was set as a fixed modification, while N-terminus cleavage and oxidation were set as variable modifications. Up to two miss-cleavages and one variable modification were allowed. MS1 and MS2 accuracies were set at 10 and 20 ppm, respectively. All other parameters were kept default. A detailed parameter table is provided in the Appendix Table S6. The raw data were searched against an in-house blood protein database. This database includes the SwissProt sequences of the 2,445 proteins that are found in blood quantified, by either ELISA or MS, in blood according to the Human Protein Atlas (acquired November 13, 2023), to which have been added the top 5% most frequent single amino acid variations found in the NextProt (Zahn-Zabal et al, 2019) database (acquired May 2024). In addition, immunoglobulin sequences have been manually added for a total of 1178 canonical sequences and 1267 mutation sequences.

Proteotypic precursors and proteins groups were filtered at 1% for Q.Value, Lib.Q.Value and Lib.PG.Q.Value. The remaining precursors were then aggregated in protein intensities, based on the Normalized.Precursor intensities, using the MaxLFQ function of the DIAgui R package (Gerault et al, 2024).

### Raw data analysis—Greifswald site

Raw data analysis was conducted using Spectronaut® version 20 in directDIA+ mode, employing reviewed Uniprot databases (*Homo sapiens*, comprising 42,529 canonical and isoform entries). Acetylation at the N-terminus of a protein, methionine oxidation were set as variable, and carbamidomethylation on cysteine residues as a static modification. The false discovery rate (FDR) for peptide-, protein-, and PSM-level analyses was set to 0.01. All tolerances were set to dynamic for pulsar searches. The XIC extraction for the MS1 and MS2 mass tolerance strategy was set to a relative 7 ppm for the timsTOF Pro2 raw data analysis. The precursor Q-value limit was set to 0.01. Files were normalized using the local normalization strategy on the human proteome. Protein inference was resolved using the IDPicker algorithm. A detailed parameter table is provided in the Appendix Table S5.

### Data analysis–Utrecht site

To identify problematic plasma samples and ensure effective quality control, the sample quality gene set list from the PlasmaProteomeProfiling (Geyer et al, 2016) study was downloaded from GitHub (https://github.com/MannLabs/Quality-Control-of-the-Plasma-Proteome/blob/master/data/Marker%20List.xlsx). Using this marker list, protein proportions for each sample were calculated, and outliers were flagged if they exceeded three standard deviations above the harmonic mean (Appendix Fig. S1). Protein concentrations were extrapolated from the protein intensities through a pseudo-external calibration approach. Briefly, housekeeping proteins with less than 30% RSD observed through two different cohorts (Gaither et al, 2020; Bizzarri et al, 2025) were used as reference proteins with their averaged absolute concentrations measured in health donors and considered as theoretical concentrations. A double log_10_ calibration curve was created reporting the theoretical concentration of these housekeeping proteins versus their intensity measured in the QC samples of pooled serum constituted of 400 healthy donors and prepared 4-6 times per sample plate (58 replicates in total). The MaxLFQ intensities were then converted into molar concentration before being converted into mass concentrations using the molar mass of the canonical protein. A UMAP analysis was conducted on plasma protein concentrations for all donors and their monthly samples within the TIMES cohort. For each donor, the median UMAP coordinates (UMAP1, UMAP2) across all longitudinal samples served as the donor-specific reference centroid. The Euclidean distance of each sample from its donor-specific centroid was calculated. A Euclidean distance greater than the mean plus five times the standard deviation across all samples, was used as a threshold to flag outliers.

### Data analysis—Greifswald site

The Spectronaut report was used as input for SpectroPipeR v0.43 (Michalik et al, 2025) to obtain batch-adjusted peptide intensities using the ComBat approach, based on the empirical Bayes methodology (Johnson et al, 2007) in the sva package (Leek et al, 2012), excluding methionine-oxidized peptides, as well as for the generation of MaxLFQ intensities. The fasta file was used to generate the number of theoretical peptides per protein using the chainsaw tool from the ProteoWizard package (Kessner et al, 2008). The sum of the peptide intensities was divided by the number of theoretical peptides to obtain the iBAQ intensities. To identify problematic plasma samples and enable effective quality control, the sample quality of the PlasmaProteomeProfiling (Geyer et al, 2016) gene set list was downloaded from github (https://github.com/MannLabs/Quality-Control-of-the-Plasma-Proteome/blob/master/data/Marker%20List.xlsx). Using this marker list protein proportions per sample were computed and outliers were flagged that exceeded three standard deviations above the harmonic mean (Appendix Fig. S1). Keratins and protein entries containing variable immunoglobulin regions were removed. The log_10_-scaled protein intensities were utilized to determine the absolute concentrations, employing the log_10_-scaled vegetative plasma protein concentrations proposed by Gaither et al, (Gaither et al, 2020) in a linear model. The model was calculated for various quantification methods (iBAQ ~ g/L; iBAQ ~ nM; MaxLFQ ~ g/L; MaxLFQ ~nM).

### General data analysis

The downstream data analysis and visualization of the data were performed using R (version 4.4.1) (Team RDC 2014) with various packages (Reagent Tools Table).

### Confounder adjustment of plasma protein concentrations

To assess the influence of biological covariates on plasma protein concentrations, four linear regression models were fitted per protein group and per study site using log_10_-transformed protein concentrations (nM) as the dependent variable: (i) age alone, (ii) sex alone, (iii) BMI alone, and (iv) age, sex, and BMI in combination. Sex was treated as a categorical variable. Models were only fitted using R (version 4.4.1). Confounder-adjusted concentrations were derived by extracting model residuals and adding back the per-protein-group mean log_10_ concentration, thereby preserving the original intensity scale while removing the variance attributable to the respective covariates. Adjusted values were subsequently back-transformed to nM. To quantify inter-donor biological variability, the coefficient of variation (CV) was calculated for each protein group and study site as the ratio of the standard deviation to the mean of per-donor average concentrations. CVs were computed separately for unadjusted data and for each of the four confounder-adjusted datasets, yielding five inter-donor CV estimates per protein group and site. Differences in inter-donor CV distributions across the five conditions were assessed with pairwise Dunn-test comparisons, applying Benjamini–Hochberg correction for multiple testing, as implemented in the ggstatsplot package (version 0.13.3).

### Multi-classification model

Plasma proteome concentration data were obtained from the Utrecht subset excluding the outlier sample (2017-12_D7). Missing values were replaced by random draws from the lower 5th percentile of the observed concentration distribution. To assess the ability of protein profiles to distinguish between individual donors, a multi-class classification approach was employed using a support vector machine (SVM) with a radial basis function (RBF) kernel, implemented via the kernlab package (Karatzoglou et al, 2004) in R. For each iteration, a leave-one-sample-per-donor-out strategy was employed: one sample per donor was randomly selected as the test set, while the remaining samples constituted the training set.

Prior to model training, all predictor variables were standardized to have a zero mean and unit variance. The modeling pipeline was constructed using the tidymodels (Max and Hadley 2020) framework, with a fixed cost hyperparameter (C = 1). Probabilistic class predictions were obtained for each test sample, and the predicted class labels were derived from the maximum probability class. Model performance was evaluated across 1000 independent iterations. For each run, accuracy, sensitivity, and specificity were computed. Sensitivity and specificity were macro-averaged across all classes to ensure equal weighting of each donor, regardless of sample size imbalance.

### Graphics

Graphics were created with BioRender.com.

## Supplementary information

Appendix

Peer Review File

Expanded View Figures

## Data Availability

Restrictions apply to the availability of data generated or analyzed during this study to preserve the anonymity of participants or because they were used under license. Access to the data is permitted under controlled access in accordance with the European Union General Data Protection Regulation (GDPR) at https://ship.limequery.org/532462?newtest=Y&lang=en. The analysis R code is available at 10.5281/zenodo.19695248. The source data of this paper are collected in the following database record: biostudies:S-SCDT-10_1038-S44320-026-00218-5.
