# Supplementary material for: Immunoglobulin sub-class levels define inter-donor plasma variability: a longitudinal dual-lab study
Source: Mol Syst Biol. 2026 May 26;22(8):1312–32. doi: 10.1038/s44320-026-00218-5 (PMC13434392; doi:10.1038/s44320-026-00218-5)
Supplement: Supplementary file 1 [file 44320_2026_218_MOESM1_ESM.pdf]

Appendix for Immunoglobulin sub-class levels define inter-donor plasma variability: a longitudinal dual-lab study

Table of contents

Plama Proteome Profiling 3

R-squared of linear modeling 3

multi-class sample prediction 4

high abundant protein examples 5

immunoglobulin sub-types and CD5L 8

single plots IgG, IgA subtypes and IgM, IgD and CD5L 9

selected inflammation markers 18

Supplementary Methods 22
- TIMES cohort - study design . . . 22
- timsTOF - diaPASEF settings . . . 23
  - diaPASEF settings timsTOF Pro 2 - Greifswald site . . . 23
  - diaPASEF settings timsTOF HT - Utrecht site . . . 23
- Spectronaut® settings . . . 24
- DIA-NN settings . . . 26

References 27

List of Figures

Appendix Figure S1 Identification of problematic plasma samples due to sampling and effective quality control of the samples using the PlasmaProteomeProfiling gene set list. The PlasmaProteomeProfiling marker list was used to compute MaxLFQ protein proportions per sample, site, and outliers exceeding three standard deviations above the harmonic mean per site were flagged. . . . 3

Appendix Figure S2 R-squared of linear modeling of absolute protein concentrations (either g/L or nM) against relative protein intensities (iBAQ or MaxLFQ) across different acquisition sites. 4

Appendix Figure S3 Results of the multi-class SVM with RBF kernel modeling. Accuracy is depicted in a boxplot. 5

Appendix Figure S4 Bar plot of Albumin (Alb) concentrations - A: Utrecht site, B: Greifswald site - The bar plot illustrates the mean and standard deviation of IgG3 concentrations across time points for each individual. Corresponding values in grams per liter (g/L) were derived from nanomolar (nM) concentrations using molecular weight-based conversion. The mean (dashed line) and standard deviation across donors are indicated in the label box. The barplot below presents the coefficient of variation (CV) across time points per donor. The inter-donor coefficient of variation is depicted as a dashed turquoise line, and its exact value is provided in the label. . 6

Appendix Figure S5 Bar plot of Transferrin (TF) concentrations - A: Utrecht site, B: Greifswald site - The bar plot illustrates the mean and standard deviation of IgG3 concentrations across time points for each individual. Corresponding values in grams per liter (g/L) were derived from nanomolar (nM) concentrations using molecular weight-based conversion. The mean (dashed line) and standard deviation across donors are indicated in the label box. The barplot below presents the coefficient of variation (CV) across time points per donor. The inter-donor coefficient of variation is depicted as a dashed turquoise line, and its exact value is provided in the label. . 7

Appendix Figure S6 Nanomolar (nM) concentrations of immunoglobulin subclasses (IgG, IgA, IgM, IgD) and CD5L from Utrecht site are visualized per donor using radar plots. In this representation, a smaller circular diameter indicates a lower relative concentration compared to other donors. The closer the plotted line approximates a circle, the more stable the concentration remains over the 12-month observation period. 8

Appendix Figure S7 Bar plot of IgG3 concentrations - A: Utrecht site, B: Greifswald site - The bar plot illustrates the mean and standard deviation of IgG3 concentrations across time points for each individual. Corresponding values in grams per liter (g/L) were derived from nanomolar (nM) concentrations using molecular weight-based conversion. The mean (dashed line) and standard deviation across donors are indicated in the label box. The barplot below presents the coefficient of variation (CV) across time points per donor. The inter-donor coefficient of variation is depicted as a dashed turquoise line, and its exact value is provided in the label. 9

Appendix Figure S8 Bar plot of IgG4 concentrations - A: Utrecht site, B: Greifswald site - The bar plot illustrates the mean and standard deviation of IgG4 concentrations across time points for each individual. Corresponding values in grams per liter (g/L) were derived from nanomolar (nM) concentrations using molecular weight-based conversion. The mean (dashed line) and standard deviation across donors are indicated in the label box. The barplot below presents the coefficient of variation (CV) across time points per donor. The inter-donor coefficient of variation is depicted as a dashed turquoise line, and its exact value is provided in the label. 10

Appendix Figure S9 Bar plot of IgA1 concentrations - A: Utrecht site, B: Greifswald site - The bar plot illustrates the mean and standard deviation of IgA1 concentrations across time points for each individual. Corresponding values in grams per liter (g/L) were derived from nanomolar (nM) concentrations using molecular weight-based conversion. The mean (dashed line) and standard deviation across donors are indicated in the label box. The barplot below presents the coefficient of variation (CV) across time points per donor. The inter-donor coefficient of variation is depicted as a dashed turquoise line, and its exact value is provided in the label. 11

Appendix Figure S10 Bar plot of IgA2 concentrations - A: Utrecht site, B: Greifswald site - The bar plot illustrates the mean and standard deviation of IgA2 concentrations across time points for each individual. Corresponding values in grams per liter (g/L) were derived from nanomolar (nM) concentrations using molecular weight-based conversion. The mean (dashed line) and standard deviation across donors are indicated in the label box. The barplot below presents the coefficient of variation (CV) across time points per donor. The inter-donor coefficient of variation is depicted as a dashed turquoise line, and its exact value is provided in the label. 12

Appendix Figure S11 Bar plot of IgD concentrations - A: Utrecht site, B: Greifswald site - The bar plot illustrates the mean and standard deviation of IgD concentrations across time points for each individual. Corresponding values in grams per liter (g/L) were derived from nanomolar (nM) concentrations using molecular weight-based conversion. The mean (dashed line) and standard deviation across donors are indicated in the label box. The barplot below presents the coefficient of variation (CV) across time points per donor. The inter-donor coefficient of variation is depicted as a dashed turquoise line, and its exact value is provided in the label. 13

Appendix Figure S12 Bar plot of IgM concentrations - A: Utrecht site, B: Greifswald site - The bar plot illustrates the mean and standard deviation of IgM concentrations across time points for each individual. Corresponding values in grams per liter (g/L) were derived from nanomolar (nM) concentrations using molecular weight-based conversion. The mean (dashed line) and standard deviation across donors are indicated in the label box. The barplot below presents the coefficient of variation (CV) across time points per donor. The inter-donor coefficient of variation is depicted as a dashed turquoise line, and its exact value is provided in the label. 14
Appendix Figure S13 Bar plot of CD5L concentrations - A: Utrecht site, B: Greifswald site - The bar plot illustrates the mean and standard deviation of CD5L concentrations across time points for each individual. Corresponding values in grams per liter (g/L) were derived from nanomolar (nM) concentrations using molecular weight-based conversion. The mean (dashed line) and standard deviation across donors are indicated in the label box. The barplot below presents the coefficient of variation (CV) across time points per donor. The inter-donor coefficient of variation is depicted as a dashed turquoise line, and its exact value is provided in the label. 15
Appendix Figure S14 Bar plot of IgG1 concentrations - A: Utrecht site, B: Greifswald site - The bar plot illustrates the mean and standard deviation of IgG1 concentrations across time points for each individual. Corresponding values in grams per liter (g/L) were derived from nanomolar (nM) concentrations using molecular weight-based conversion. The mean (dashed line) and standard deviation across donors are indicated in the label box. The barplot below presents the coefficient of variation (CV) across time points per donor. The inter-donor coefficient of variation is depicted as a dashed turquoise line, and its exact value is provided in the label. 16
Appendix Figure S15 Bar plot of IgG2 concentrations - A: Utrecht site, B: Greifswald site - The bar plot illustrates the mean and standard deviation of IgG2 concentrations across time points for each individual. Corresponding values in grams per liter (g/L) were derived from nanomolar (nM) concentrations using molecular weight-based conversion. The mean (dashed line) and standard deviation across donors are indicated in the label box. The barplot below presents the coefficient of variation (CV) across time points per donor. The inter-donor coefficient of variation is depicted as a dashed turquoise line, and its exact value is provided in the label. 17
Appendix Figure S16 CRP - A: Utrecht site, B: Greifswald site - Mean concentration shown in barplot (top panel). The tile plot below (lower panel) depicts the temporal profiles of CRP in donors (grey color represent missing values). Color scale boundaries were defined using log10-transformed donor specific averaged nM estimated values. The lower bound was set at the 1st percentile, and the upper bound at the 99th percentile. 18
Appendix Figure S17 SAA1 - A: Utrecht site, B: Greifswald site - Mean concentration shown in barplot (top panel). The tile plot below (lower panel) depicts the temporal profiles of SAA1 in donors (grey color represent missing values). Color scale boundaries were defined using log10-transformed donor specific averaged nM estimated values. The lower bound was set at the 1st percentile, and the upper bound at the 99th percentile. 19
Appendix Figure S18 SAA2 - A: Utrecht site, B: Greifswald site - Mean concentration shown in barplot (top panel). The tile plot below (lower panel) depicts the temporal profiles of SAA2 in donors (grey color represent missing values). Color scale boundaries were defined using log10-transformed donor specific averaged nM estimated values. The lower bound was set at the 1st percentile, and the upper bound at the 99th percentile. 20
Appendix Figure S19 S100A8 - A: Utrecht site, B: Greifswald site - Mean concentration shown in barplot (top panel). The tile plot below (lower panel) depicts the temporal profiles of S100A8 in donors (grey color represent missing values). Color scale boundaries were defined using log10-transformed donor specific averaged nM estimated values. The lower bound was set at the 1st percentile, and the upper bound at the 99th percentile. 21
Appendix Figure S20 S100A9 - A: Utrecht site, B: Greifswald site - Mean concentration shown in barplot (top panel). The tile plot below (lower panel) depicts the temporal profiles of S100A9 in donors (grey color represent missing values). Color scale boundaries were defined using log10-transformed donor specific averaged nM estimated values. The lower bound was set at the 1st percentile, and the upper bound at the 99th percentile. 22

List of Tables

Appendix Table S1 reference plasma protein concentrations. 3
Appendix Table S2 TIMES cohort overview 23
Appendix Table S3 diaPASEF windows timsTOF Pro 2 23
Appendix Table S4 diaPASEF windows timsTOF HT 23
Appendix Table S5 Spectronaut® settings 24
Appendix Table S6 DIA-NN settings 26

Plama Proteome Profiling

To evaluate the quality of the plasma samples the methodology from Geyer *et al* (2019) was used. Using the MaxLFQ protein intensities, proportions were calculated for platelets, erythrocytes, and coagulation contamination using their marker panels. This approach showed that only a marginal number of samples possess higher contaminations for platelets, erythrocytes, or coagulation (Appendix Figure S1) and that the overall sample quality of the cohort is in an acceptable range.

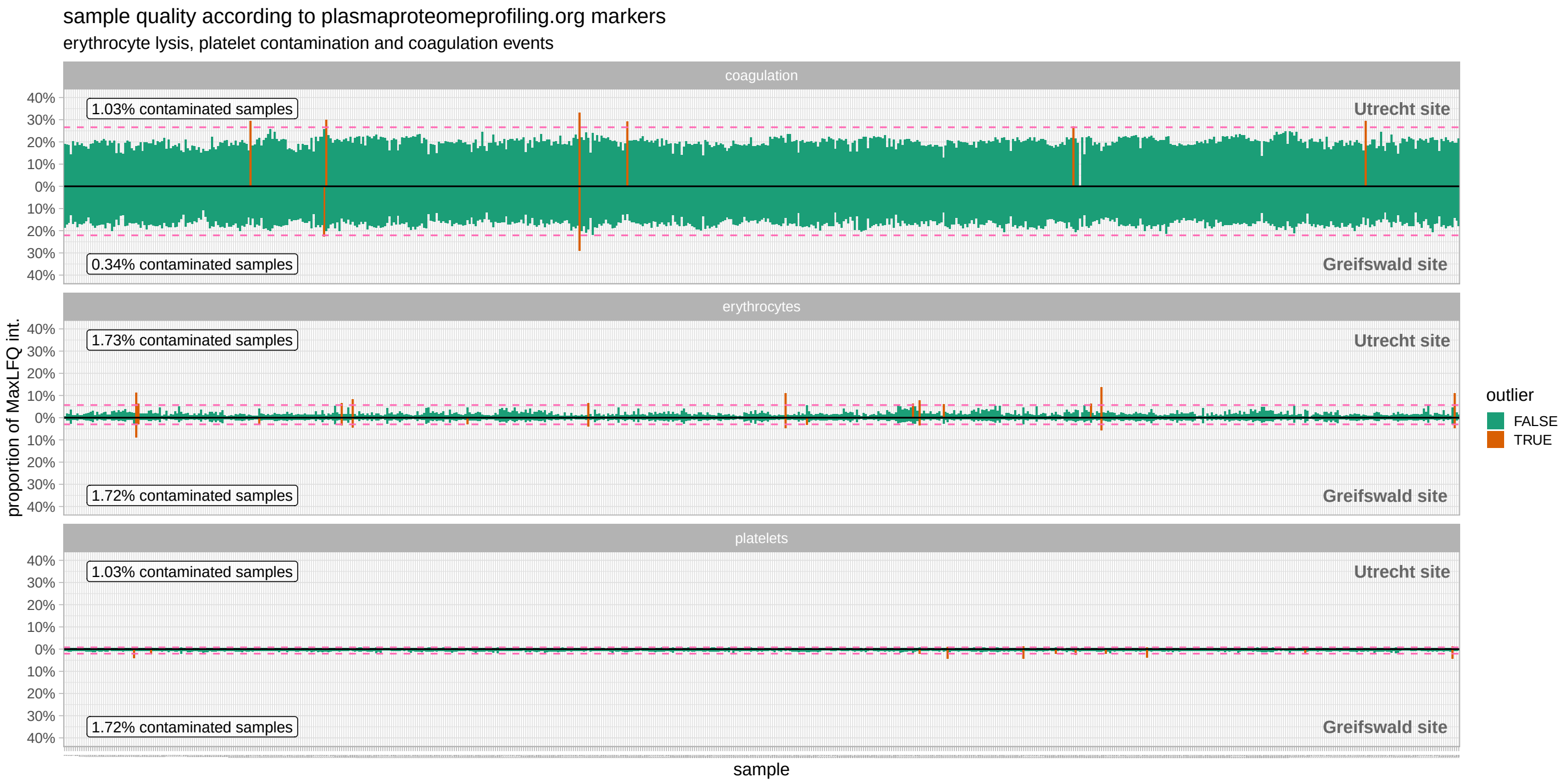

Appendix Figure S1: Identification of problematic plasma samples due to sampling and effective quality control of the samples using the PlasmaProteomeProfiling gene set list. The PlasmaProteomeProfiling marker list was used to compute MaxLFQ protein proportions per sample, site, and outliers exceeding three standard deviations above the harmonic mean per site were flagged.

R-squared of linear modeling

The log10-scaled protein intensities were used to determine the absolute concentrations of proteins. This was done by fitting a linear model to the log10-scaled reference plasma protein concentrations (Appendix Table S1) measured by Gaither *et al* (2020). The model was tested for different combinations, including iBAQ ~ g/l, iBAQ ~ nM, MaxLFQ ~ g/l, and MaxLFQ ~ nM. For the Greifswald site, the iBAQ~nM model showed the best performance (Appendix Figure S2), whereas for the Utrecht site the MaxLFQ~g/l and MaxLFQ~nM performed the equally better. We proceeded with the MaxLFQ~nM model, to achieve better comparisons with the Greifswald site.

Appendix Table S1: reference plasma protein concentrations.

uniprot entry	protein names	genes	mass [Da]	ref. conc. [fmol/μl]	ref. conc. [g/ml]
P02768	ALBU_HUMAN	ALB	69367	623874.66	4.33e-02
P01009	A1AT_HUMAN	SERPINA1	46737	18179.60	8.50e-04
P02787	TRFE_HUMAN	TF	77050	16981.75	1.31e-03
P02652	APOA2_HUMAN	APOA2	11175	9317.08	1.04e-04
P02790	HEMO_HUMAN	HPX	51676	8902.73	4.60e-04
P02765	FETUA_HUMAN	AHSG	39341	3958.15	1.56e-04
P02774	VTDB_HUMAN	GC	52918	3478.03	1.84e-04
P00734	THRB_HUMAN	F2	70037	2960.97	2.07e-04
P04003	C4BPA_HUMAN	C4BPA	67033	1853.66	1.24e-04
P01042	KNG1_HUMAN	KNG1	71957	1797.13	1.29e-04
P04217	A1BG_HUMAN	A1BG	54254	1729.38	9.38e-05
P10909	CLUS_HUMAN	CLU	52495	1368.92	7.19e-05
P19823	ITIH2_HUMAN	ITIH2	106463	1115.10	1.19e-04
P05546	HEP2_HUMAN	SERPIND1	57071	883.44	5.04e-05
P08697	A2AP_HUMAN	SERPINF2	54566	741.50	4.05e-05

(continued)

uniprot entry	protein names	genes	mass [Da]	ref. conc. [fmol/μl]	ref. conc. [g/ml]
P02747	C1QC_HUMAN	C1QC	25774	622.70	1.60e-05
P00747	PLMN_HUMAN	PLG	90569	470.07	4.26e-05
P05156	CFAI_HUMAN	CFI	65750	332.56	2.19e-05
P09871	C1S_HUMAN	C1S	76684	309.29	2.37e-05
P25311	ZA2G_HUMAN	AZGP1	34259	296.62	1.02e-05
P00736	C1R_HUMAN	C1R	80119	283.52	2.27e-05
P07225	PROS_HUMAN	PROS1	75123	271.04	2.04e-05
P36955	PEDF_HUMAN	SERPINF1	46312	234.36	1.09e-05
P02746	C1QB_HUMAN	C1QB	26722	212.78	5.70e-06
P07357	CO8A_HUMAN	C8A	65163	176.17	1.15e-05
Q14520	HABP2_HUMAN	HABP2	62672	164.95	1.03e-05
P01031	CO5_HUMAN	C5	188305	163.46	3.08e-05
P13671	CO6_HUMAN	C6	104786	145.14	1.52e-05
P02745	C1QA_HUMAN	C1QA	26017	123.06	3.20e-06
P00742	FA10_HUMAN	F10	54732	121.79	6.70e-06
O75882	ATRN_HUMAN	ATRN	158537	119.06	1.89e-05
P15169	CBPN_HUMAN	CPN1	52286	115.29	6.00e-06
P07358	CO8B_HUMAN	C8B	66948	96.28	6.50e-06
Q96IY4	CBPB2_HUMAN	CPB2	48424	72.08	3.50e-06
Q9NZP8	C1RL_HUMAN	C1RL	53498	52.66	2.80e-06
P22792	CPN2_HUMAN	CPN2	60557	42.19	2.60e-06
Q6EMK4	VASN_HUMAN	VASN	71713	12.89	9.00e-07

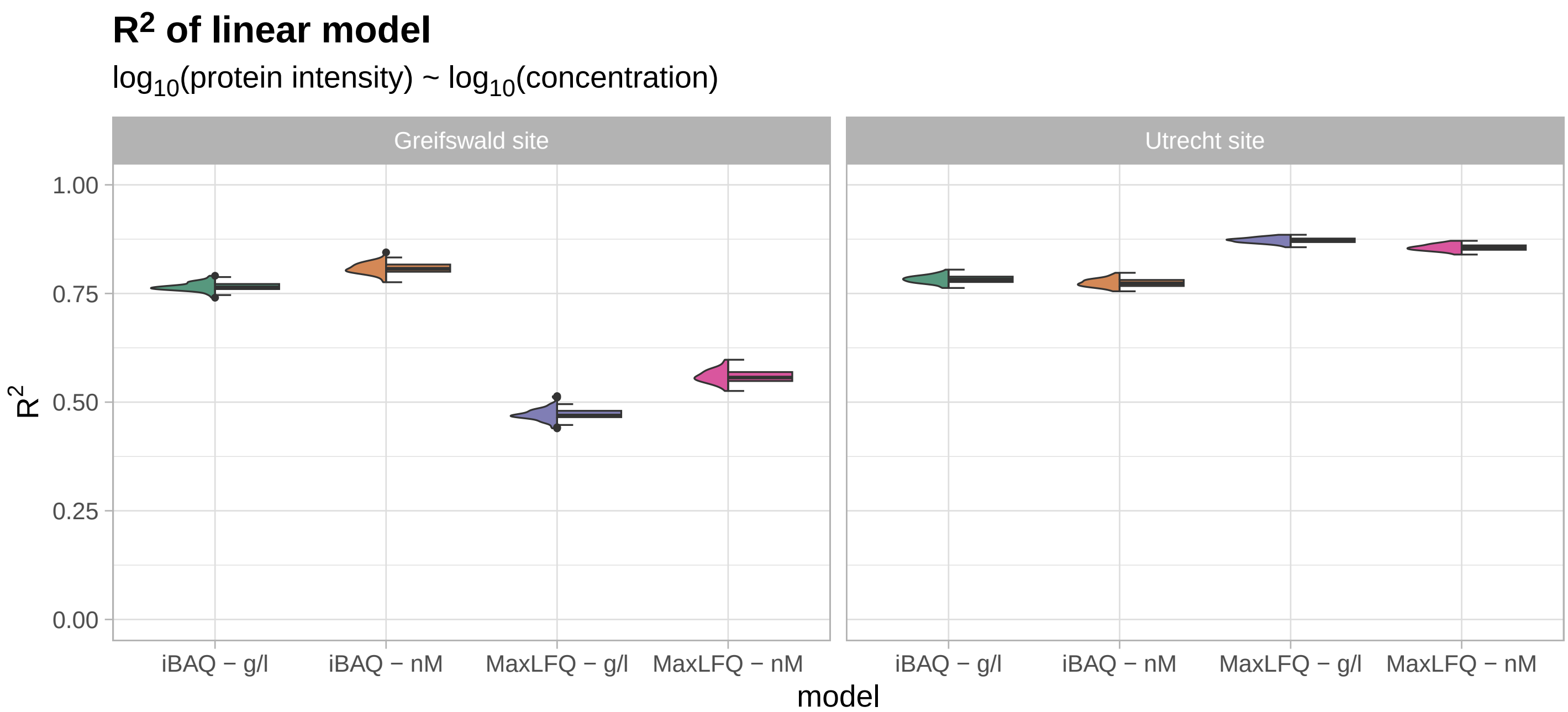

Appendix Figure S2: R-squared of linear modeling of absolute protein concentrations (either g/L or nM) against relative protein intensities (iBAQ or MaxLFQ) across different acquisition sites.

multi-class sample prediction

To determine the feasibility of re-attributing a random sample from a donor to its specific donor using the quantitative plasma protein profile, we employed a leave-one-out multi-class support vector machine (SVM) modeling approach (Appendix Figure S3). Therefore, missing values were imputed by randomly sampling from the lower 5th percentile of observed concentrations. The predictors were standardized to maintain consistency across the analysis, and a fixed cost parameter (C = 1) was applied. After 1000 iterations probabilistic predictions were generated, and performance metrics were computed. The SVM achieved an accuracy of 0.98 in the median, indicating that when using a blinded random single sample with extremely high certainty, it can be accurately assigned to a donor.

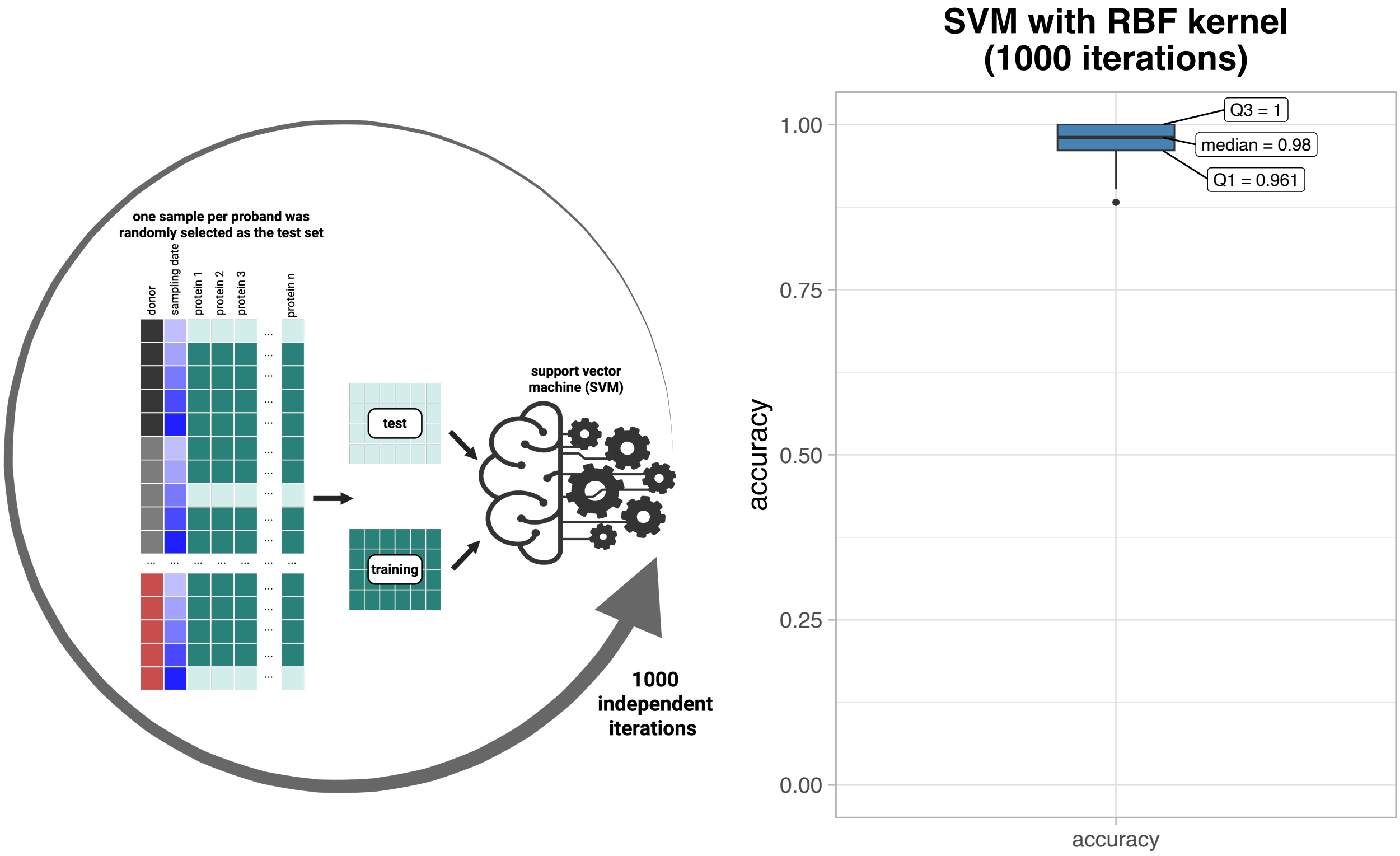

Appendix Figure S3: Results of the multi-class SVM with RBF kernel modeling. Accuracy is depicted in a boxplot.

high abundant protein examples

The Greifswald site utilized the timsTOF Pro2, while the Utrecht site employed the timsTOF HT. Saturation may occur during the measurement of highly abundant plasma proteins (e.g. albumin and transferrin), potentially leading to a reduction in inter-donor variability. In the timsTOF Pro2 measurements, the inter-donor coefficient of variation (CV) is significantly lower compared to the timsTOF HT measurements, suggesting a saturation issue with the methodological workflow used for the timsTOF Pro2 (Appendix Figure S4, Appendix Figure S5).

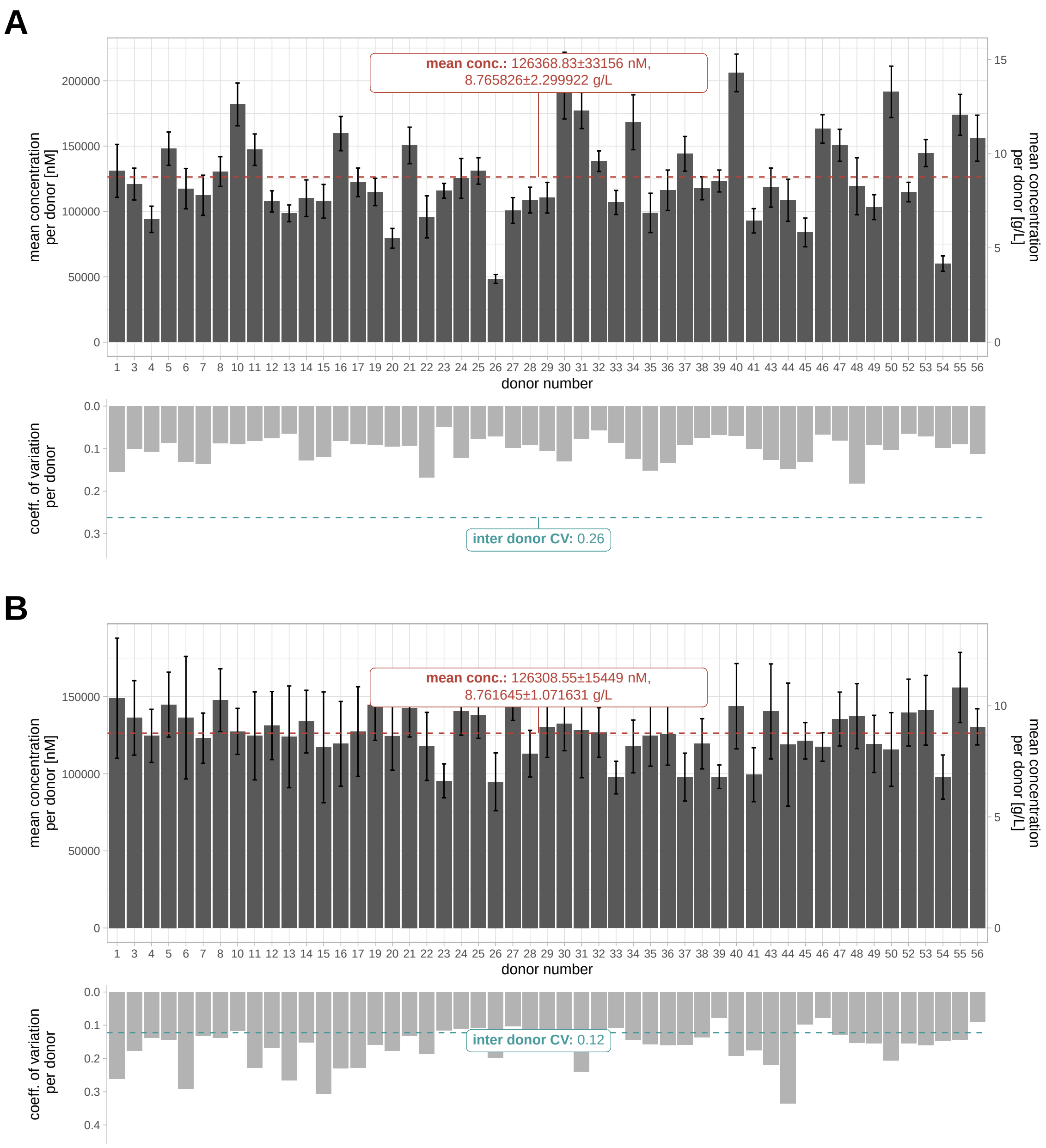

Appendix Figure S4: Bar plot of Albumin (Alb) concentrations - A: Utrecht site, B: Greifswald site - The bar plot illustrates the mean and standard deviation of IgG3 concentrations across time points for each individual. Corresponding values in grams per liter (g/L) were derived from nanomolar (nM) concentrations using molecular weight-based conversion. The mean (dashed line) and standard deviation across donors are indicated in the label box. The barplot below presents the coefficient of variation (CV) across time points per donor. The inter-donor coefficient of variation is depicted as a dashed turquoise line, and its exact value is provided in the label.

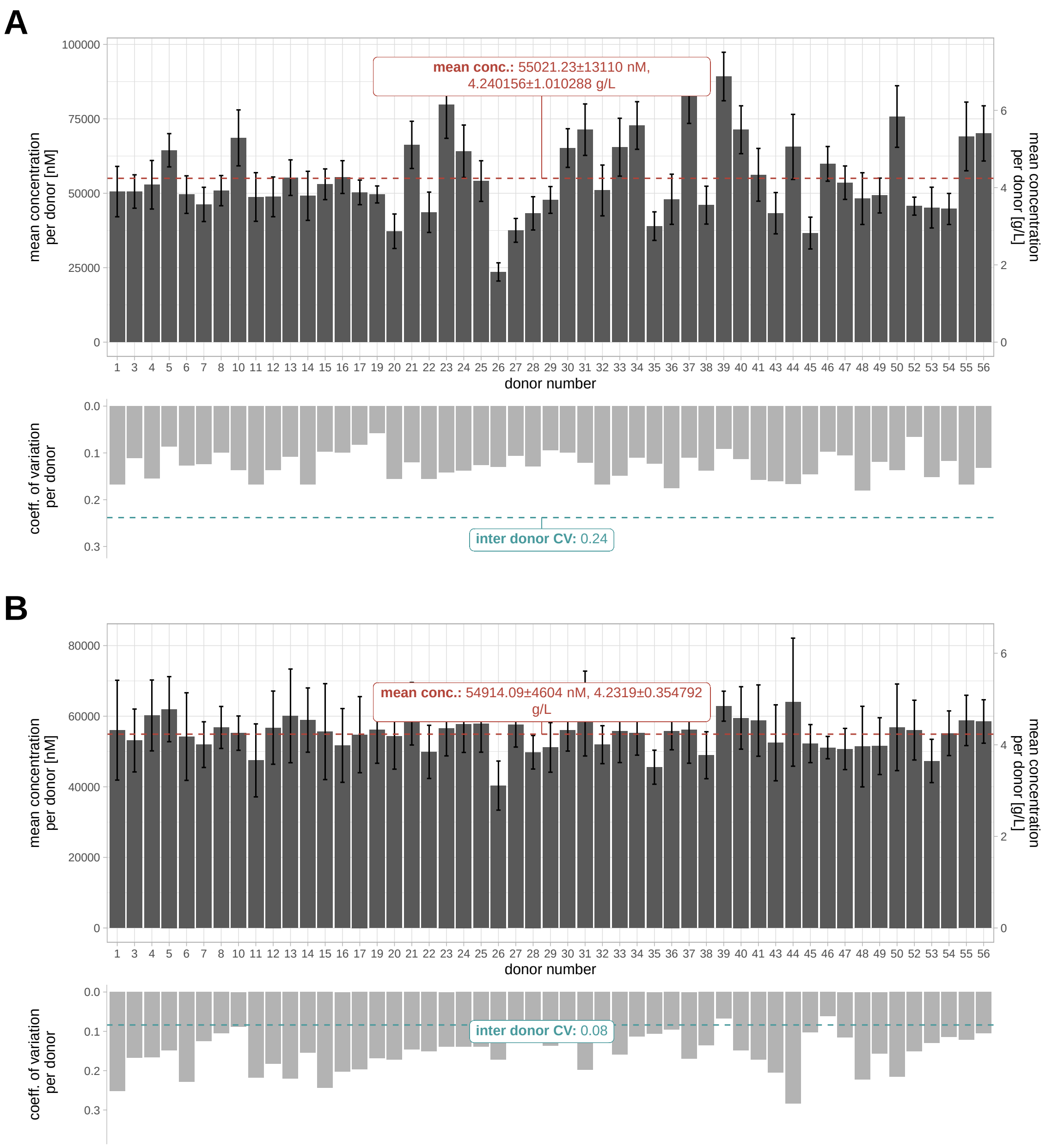

Appendix Figure S5: Bar plot of Transferrin (TF) concentrations - A: Utrecht site, B: Greifswald site - The bar plot illustrates the mean and standard deviation of IgG3 concentrations across time points for each individual. Corresponding values in grams per liter (g/L) were derived from nanomolar (nM) concentrations using molecular weight-based conversion. The mean (dashed line) and standard deviation across donors are indicated in the label box. The barplot below presents the coefficient of variation (CV) across time points per donor. The inter-donor coefficient of variation is depicted as a dashed turquoise line, and its exact value is provided in the label.

immunoglobulin sub-types and CD5L

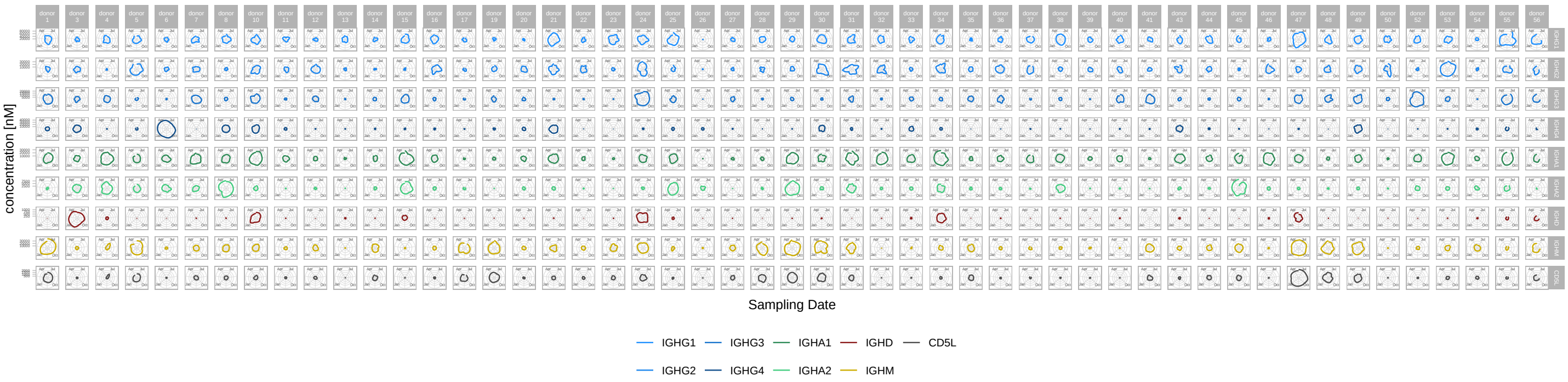

Appendix Figure S6: Nanomolar (nM) concentrations of immunoglobulin subclasses (IgG, IgA, IgM, IgD) and CD5L from Utrecht site are visualized per donor using radar plots. In this representation, a smaller circular diameter indicates a lower relative concentration compared to other donors. The closer the plotted line approximates a circle, the more stable the concentration remains over the 12-month observation period.

single plots IgG, IgA subtypes and IgM, IgD and CD5L

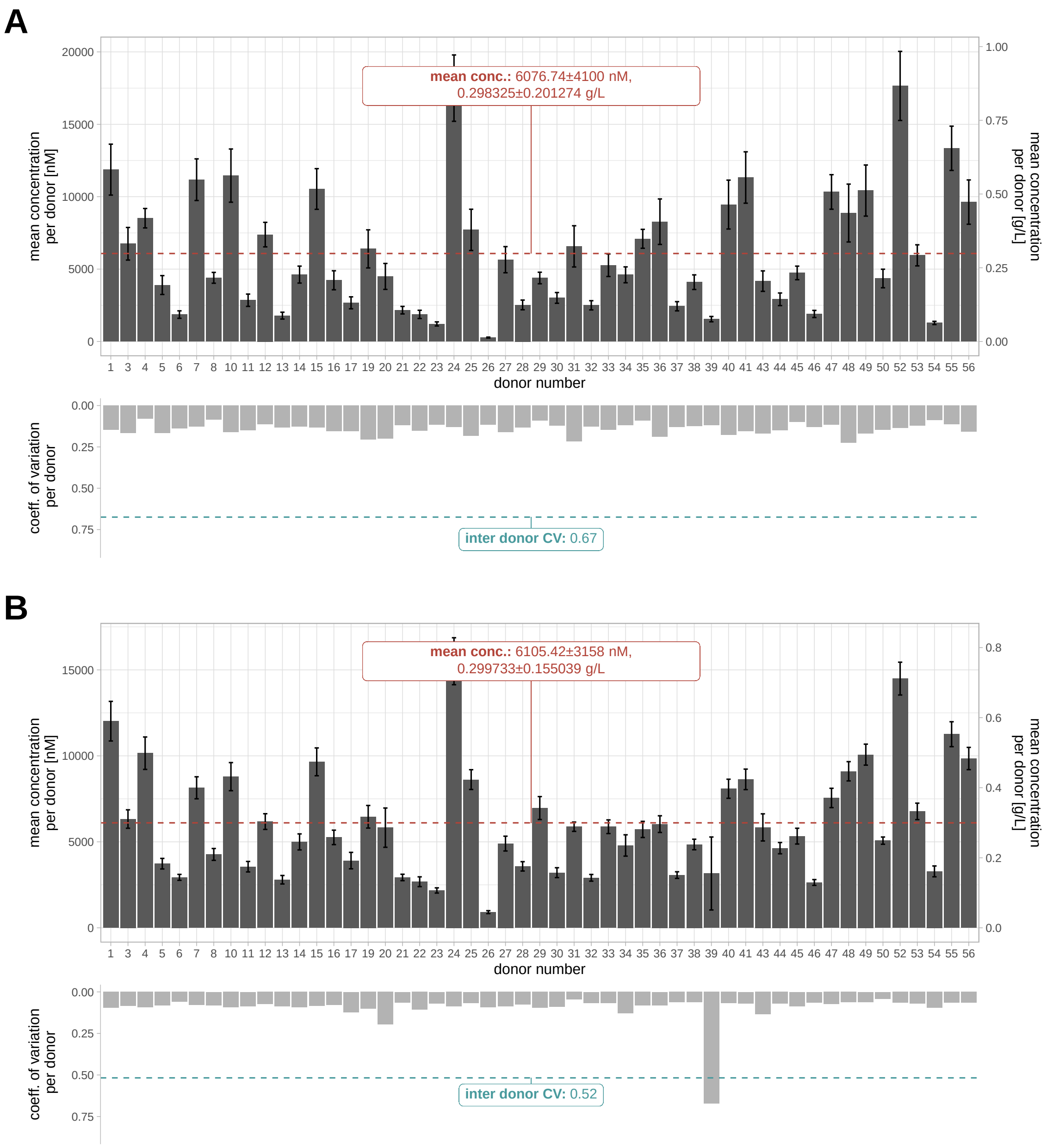

Appendix Figure S7: Bar plot of IgG3 concentrations - A: Utrecht site, B: Greifswald site - The bar plot illustrates the mean and standard deviation of IgG3 concentrations across time points for each individual. Corresponding values in grams per liter (g/L) were derived from nanomolar (nM) concentrations using molecular weight-based conversion. The mean (dashed line) and standard deviation across donors are indicated in the label box. The barplot below presents the coefficient of variation (CV) across time points per donor. The inter-donor coefficient of variation is depicted as a dashed turquoise line, and its exact value is provided in the label.

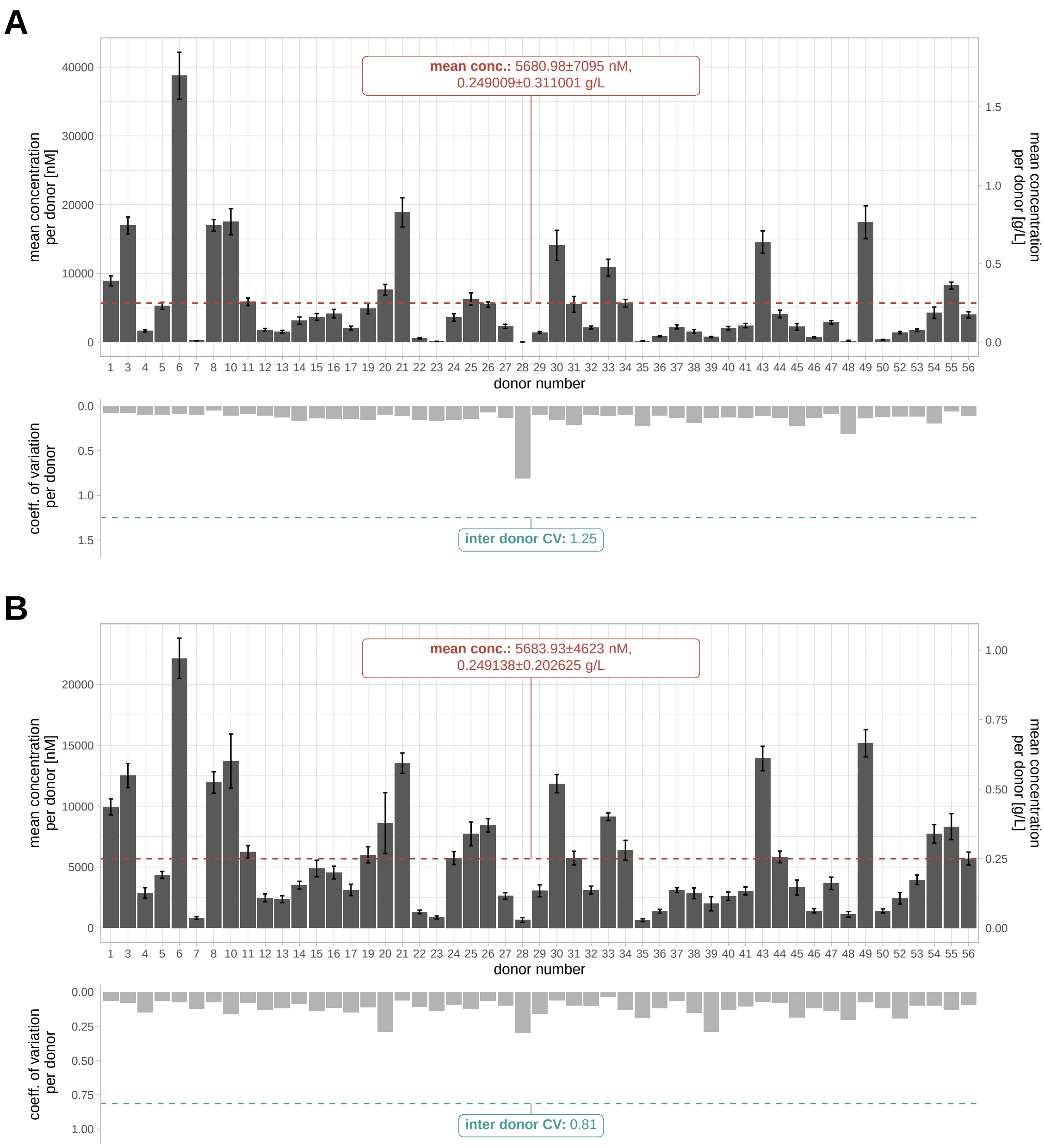

Appendix Figure S8: Bar plot of IgG4 concentrations - A: Utrecht site, B: Greifswald site - The bar plot illustrates the mean and standard deviation of IgG4 concentrations across time points for each individual. Corresponding values in grams per liter (g/L) were derived from nanomolar (nM) concentrations using molecular weight-based conversion. The mean (dashed line) and standard deviation across donors are indicated in the label box. The barplot below presents the coefficient of variation (CV) across time points per donor. The inter-donor coefficient of variation is depicted as a dashed turquoise line, and its exact value is provided in the label.

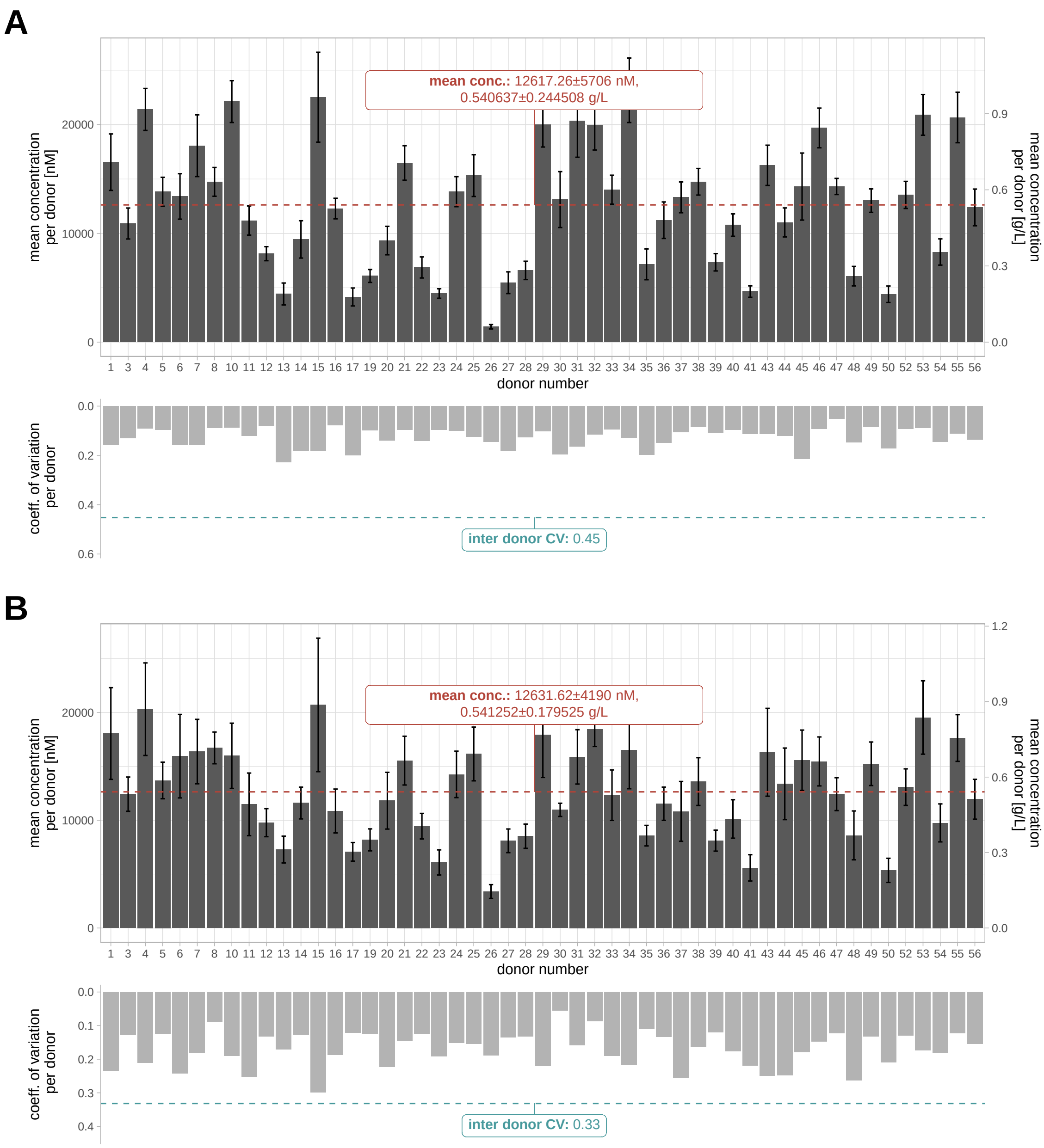

Appendix Figure S9: Bar plot of IgA1 concentrations - A: Utrecht site, B: Greifswald site - The bar plot illustrates the mean and standard deviation of IgA1 concentrations across time points for each individual. Corresponding values in grams per liter (g/L) were derived from nanomolar (nM) concentrations using molecular weight-based conversion. The mean (dashed line) and standard deviation across donors are indicated in the label box. The barplot below presents the coefficient of variation (CV) across time points per donor. The inter-donor coefficient of variation is depicted as a dashed turquoise line, and its exact value is provided in the label.

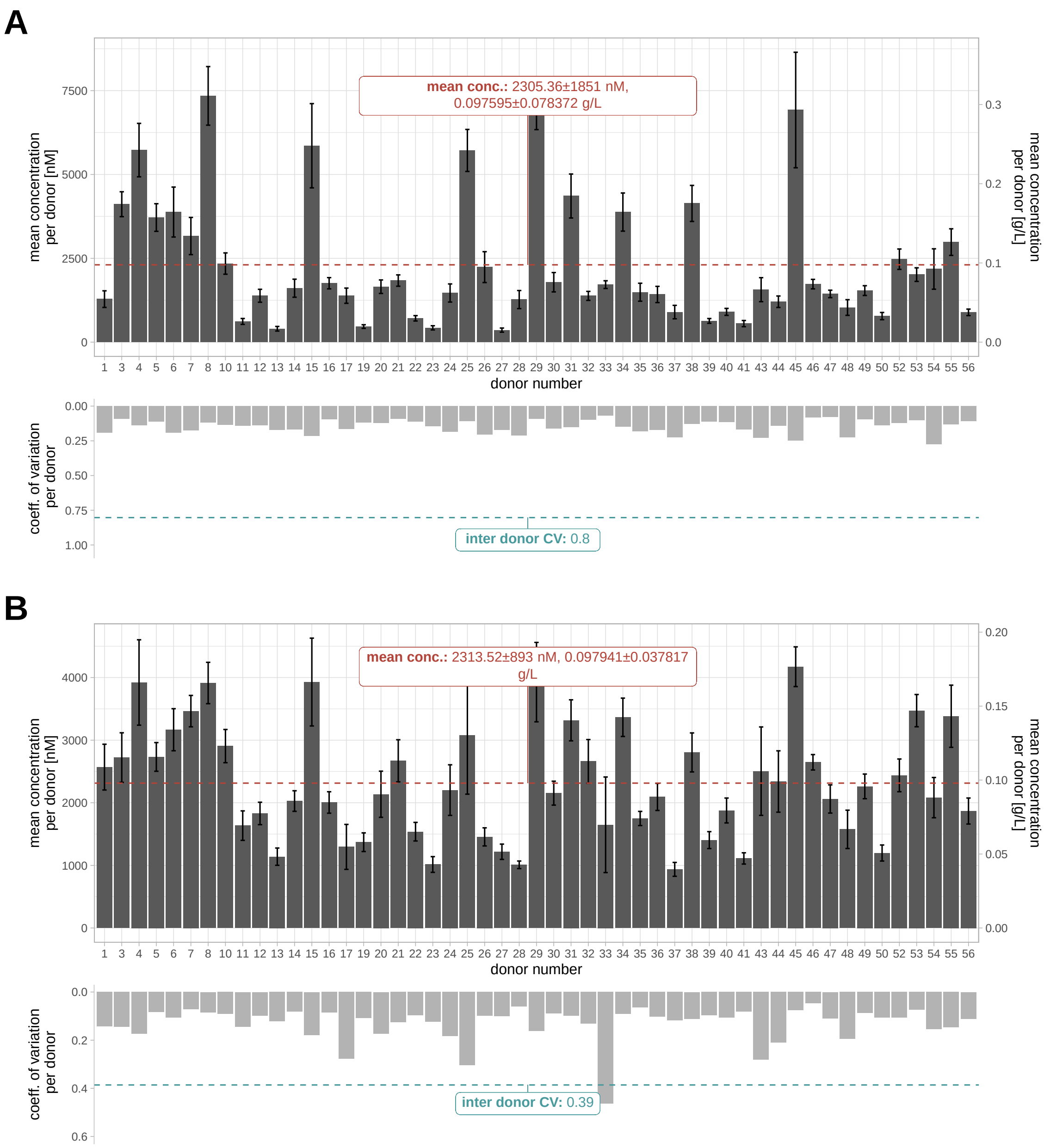

Appendix Figure S10: Bar plot of IgA2 concentrations - A: Utrecht site, B: Greifswald site - The bar plot illustrates the mean and standard deviation of IgA2 concentrations across time points for each individual. Corresponding values in grams per liter (g/L) were derived from nanomolar (nM) concentrations using molecular weight-based conversion. The mean (dashed line) and standard deviation across donors are indicated in the label box. The barplot below presents the coefficient of variation (CV) across time points per donor. The inter-donor coefficient of variation is depicted as a dashed turquoise line, and its exact value is provided in the label.

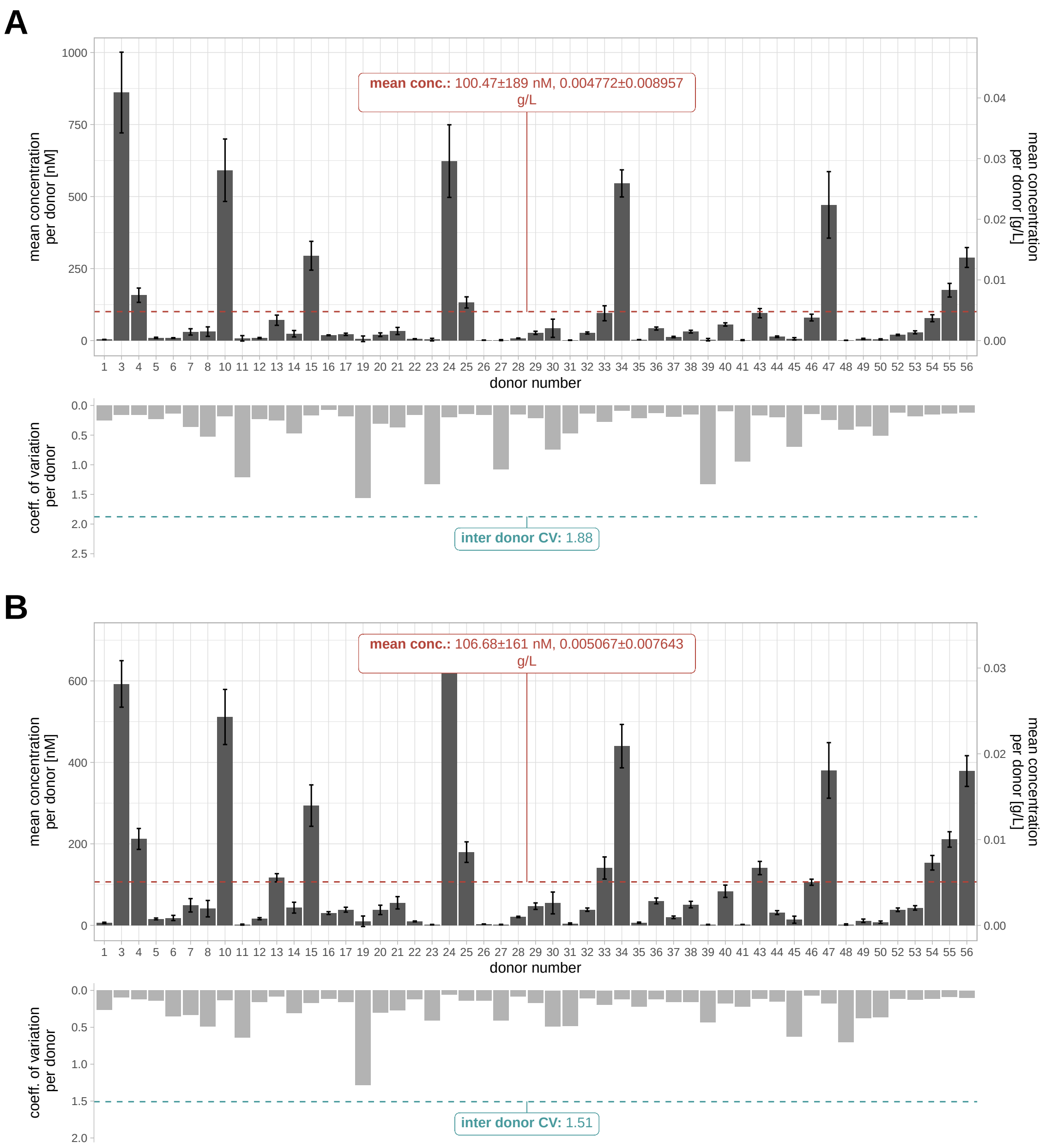

Appendix Figure S11: Bar plot of IgD concentrations - A: Utrecht site, B: Greifswald site - The bar plot illustrates the mean and standard deviation of IgD concentrations across time points for each individual. Corresponding values in grams per liter (g/L) were derived from nanomolar (nM) concentrations using molecular weight-based conversion. The mean (dashed line) and standard deviation across donors are indicated in the label box. The barplot below presents the coefficient of variation (CV) across time points per donor. The inter-donor coefficient of variation is depicted as a dashed turquoise line, and its exact value is provided in the label.

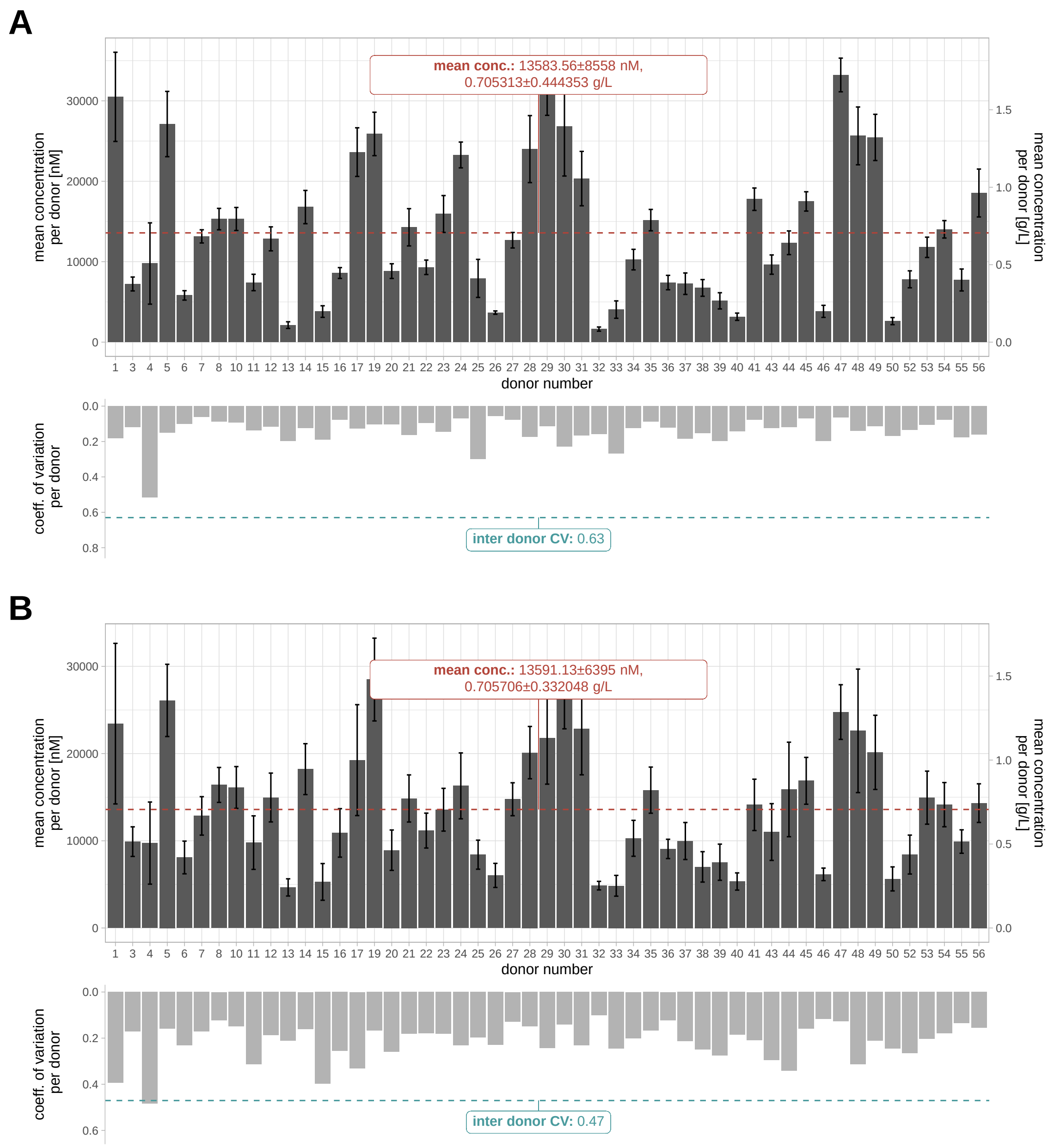

Appendix Figure S12: Bar plot of IgM concentrations - A: Utrecht site, B: Greifswald site - The bar plot illustrates the mean and standard deviation of IgM concentrations across time points for each individual. Corresponding values in grams per liter (g/L) were derived from nanomolar (nM) concentrations using molecular weight-based conversion. The mean (dashed line) and standard deviation across donors are indicated in the label box. The barplot below presents the coefficient of variation (CV) across time points per donor. The inter-donor coefficient of variation is depicted as a dashed turquoise line, and its exact value is provided in the label.

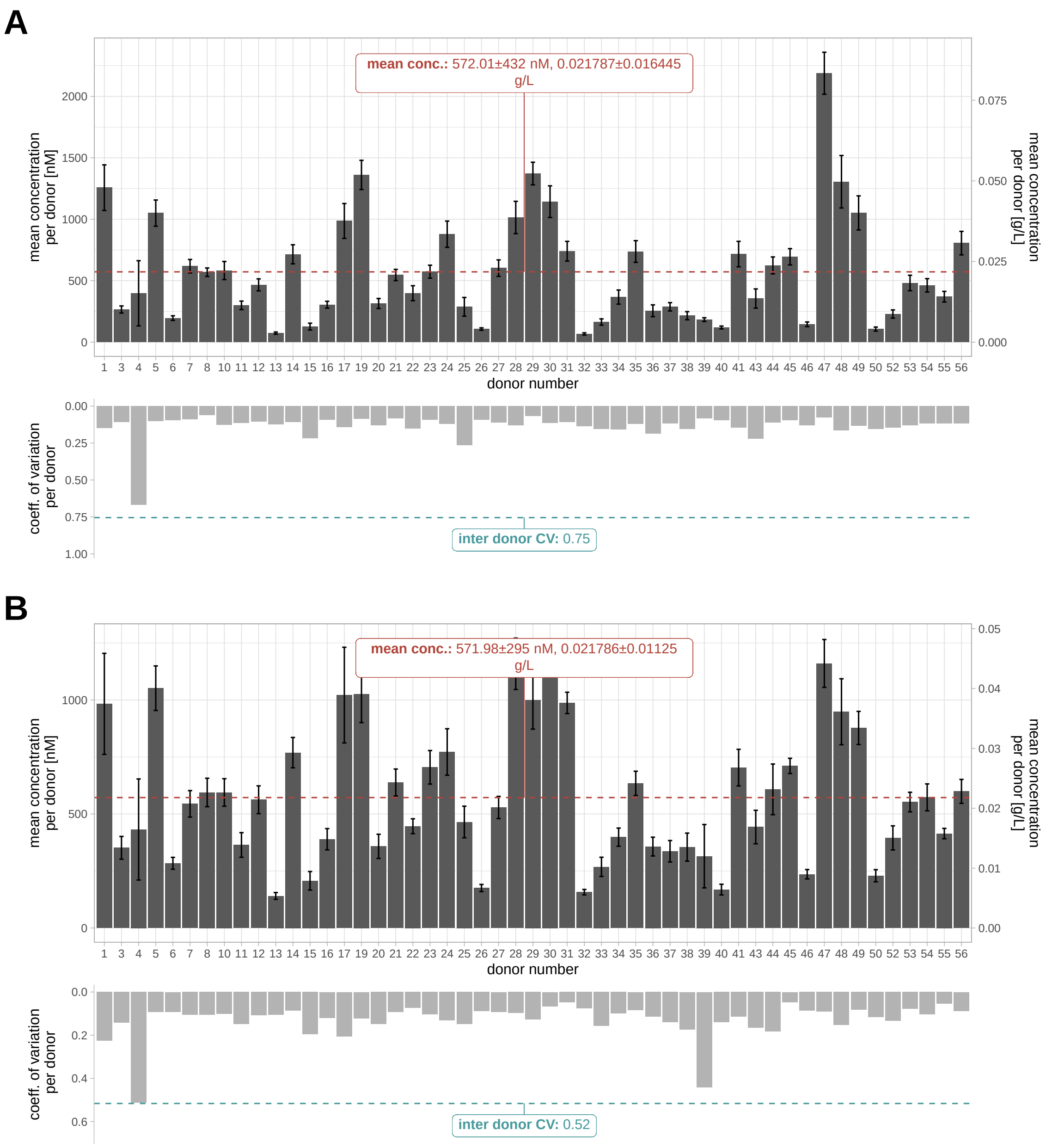

Appendix Figure S13: Bar plot of CD5L concentrations - A: Utrecht site, B: Greifswald site - The bar plot illustrates the mean and standard deviation of CD5L concentrations across time points for each individual. Corresponding values in grams per liter (g/L) were derived from nanomolar (nM) concentrations using molecular weight-based conversion. The mean (dashed line) and standard deviation across donors are indicated in the label box. The barplot below presents the coefficient of variation (CV) across time points per donor. The inter-donor coefficient of variation is depicted as a dashed turquoise line, and its exact value is provided in the label.

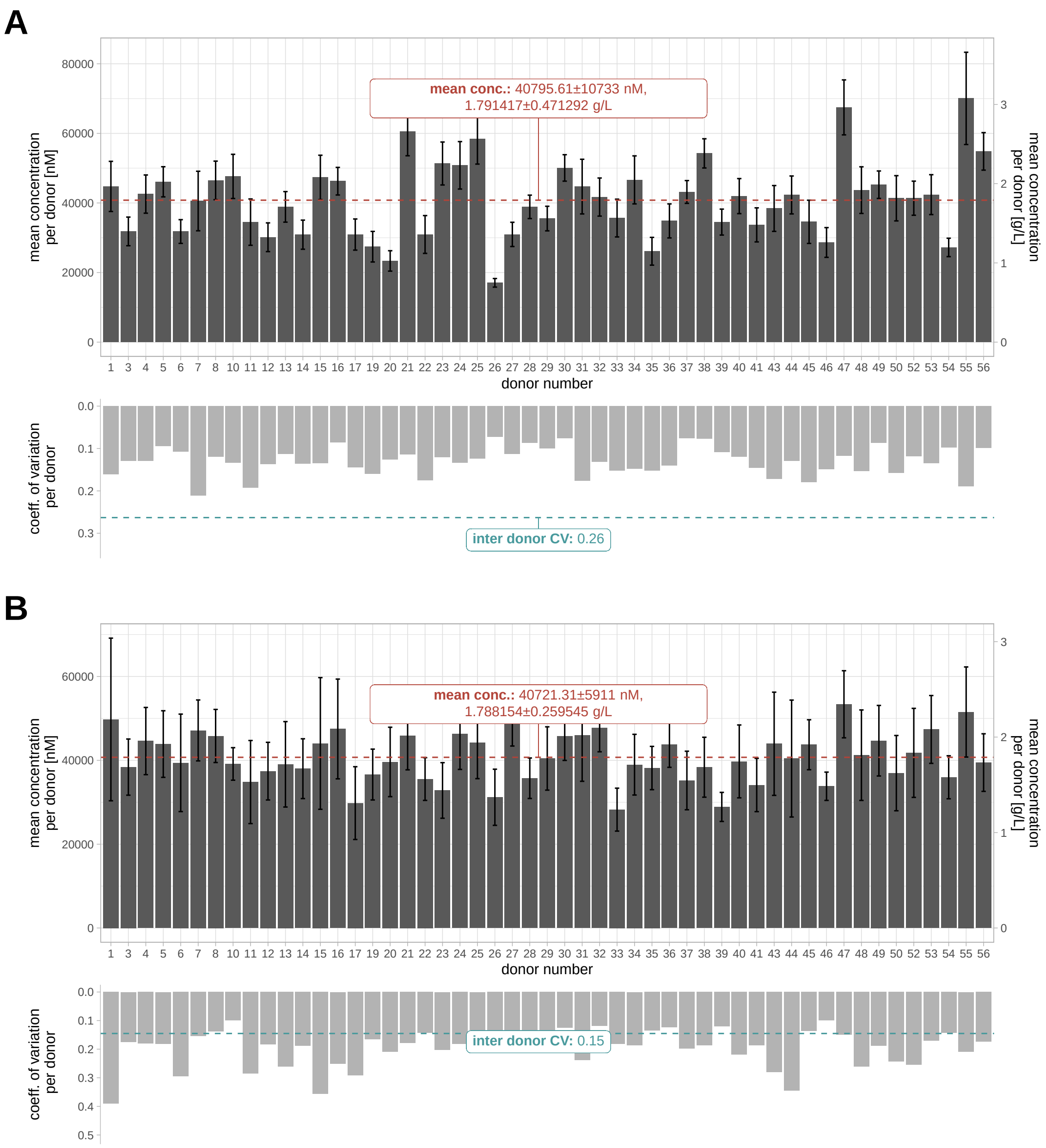

Appendix Figure S14: Bar plot of IgG1 concentrations - A: Utrecht site, B: Greifswald site - The bar plot illustrates the mean and standard deviation of IgG1 concentrations across time points for each individual. Corresponding values in grams per liter (g/L) were derived from nanomolar (nM) concentrations using molecular weight-based conversion. The mean (dashed line) and standard deviation across donors are indicated in the label box. The barplot below presents the coefficient of variation (CV) across time points per donor. The inter-donor coefficient of variation is depicted as a dashed turquoise line, and its exact value is provided in the label.

A

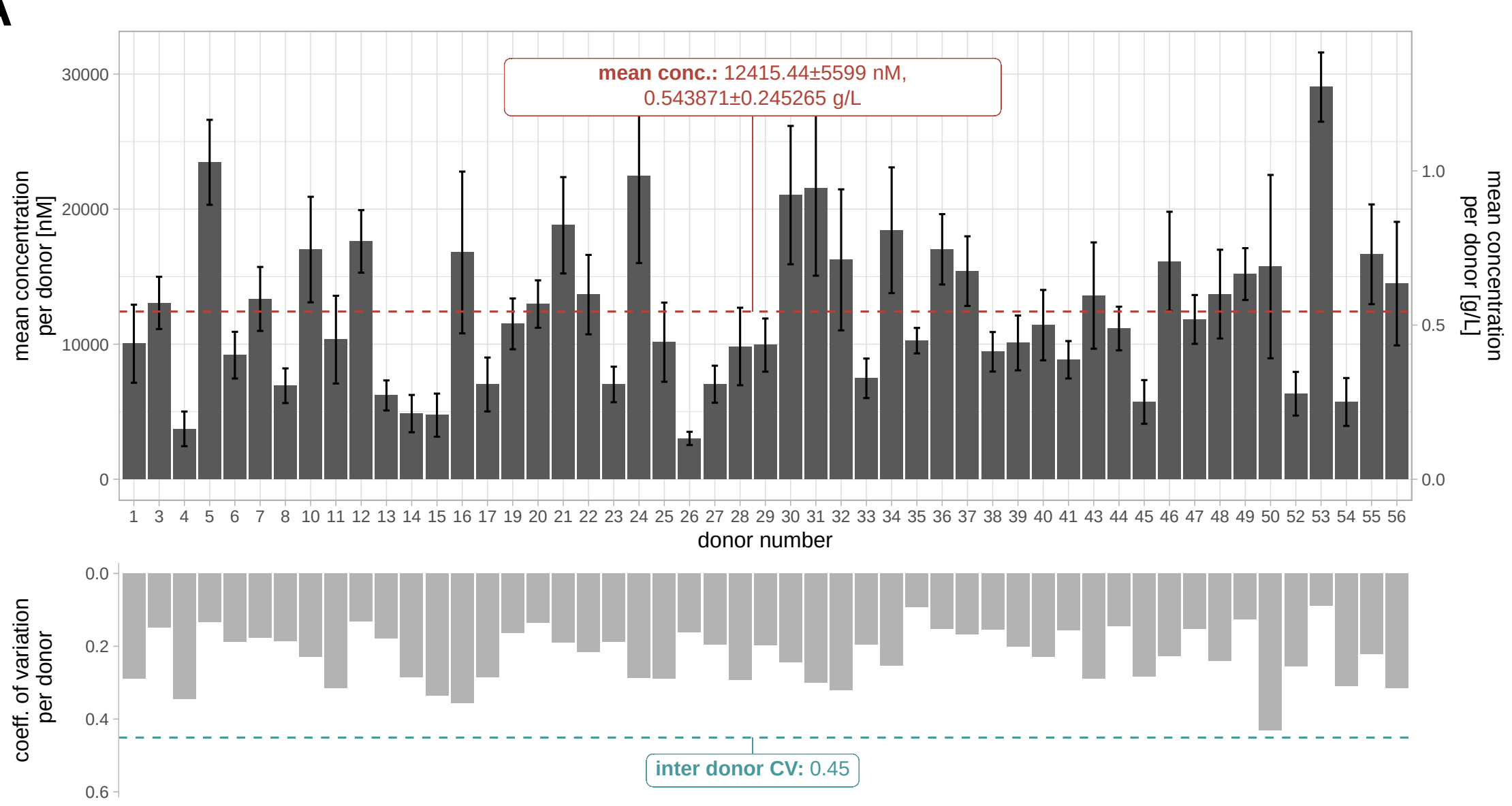

B

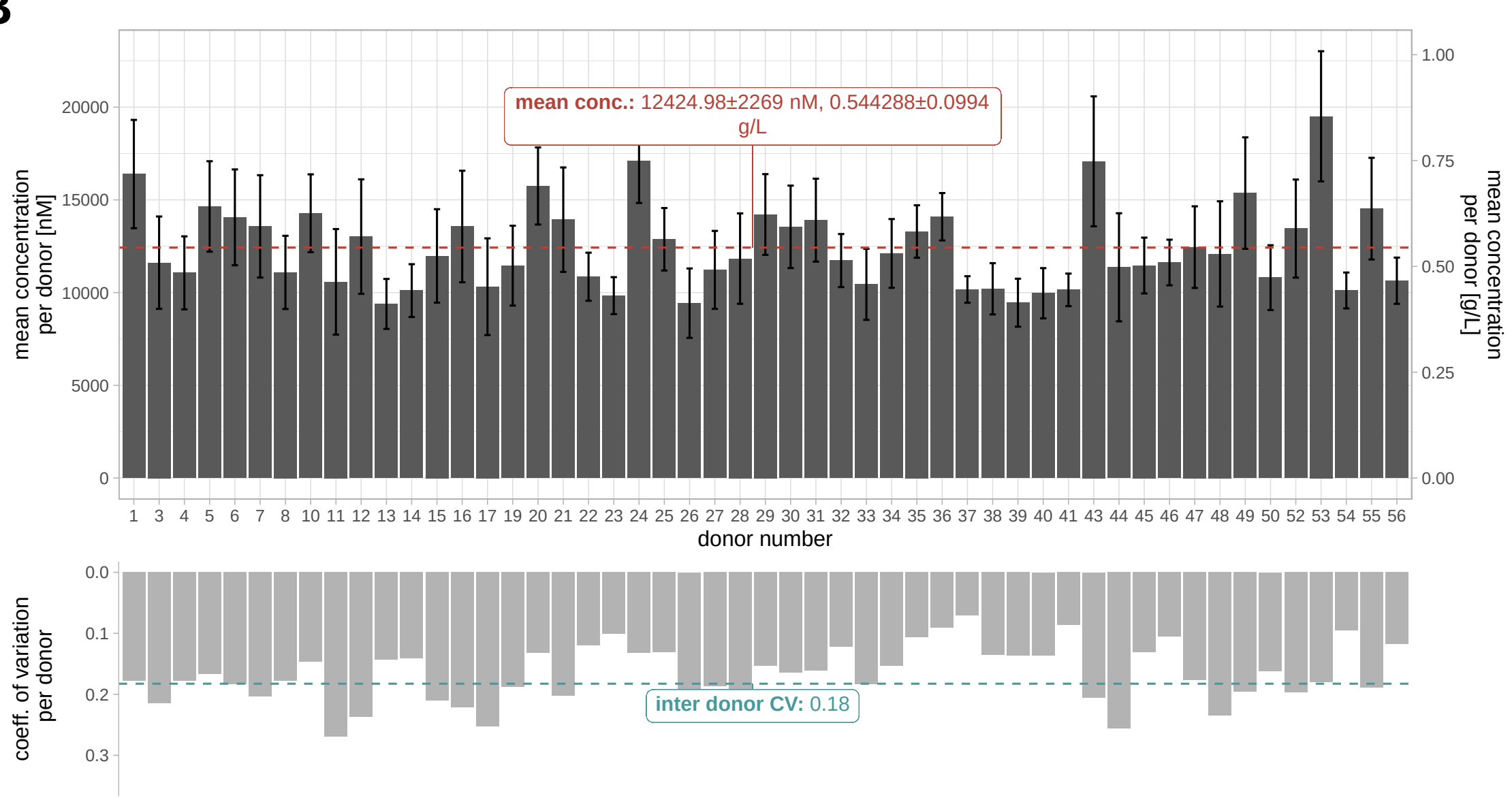

Appendix Figure S15: Bar plot of IgG2 concentrations - A: Utrecht site, B: Greifswald site - The bar plot illustrates the mean and standard deviation of IgG2 concentrations across time points for each individual. Corresponding values in grams per liter (g/L) were derived from nanomolar (nM) concentrations using molecular weight-based conversion. The mean (dashed line) and standard deviation across donors are indicated in the label box. The barplot below presents the coefficient of variation (CV) across time points per donor. The inter-donor coefficient of variation is depicted as a dashed turquoise line, and its exact value is provided in the label.

selected inflammation markers

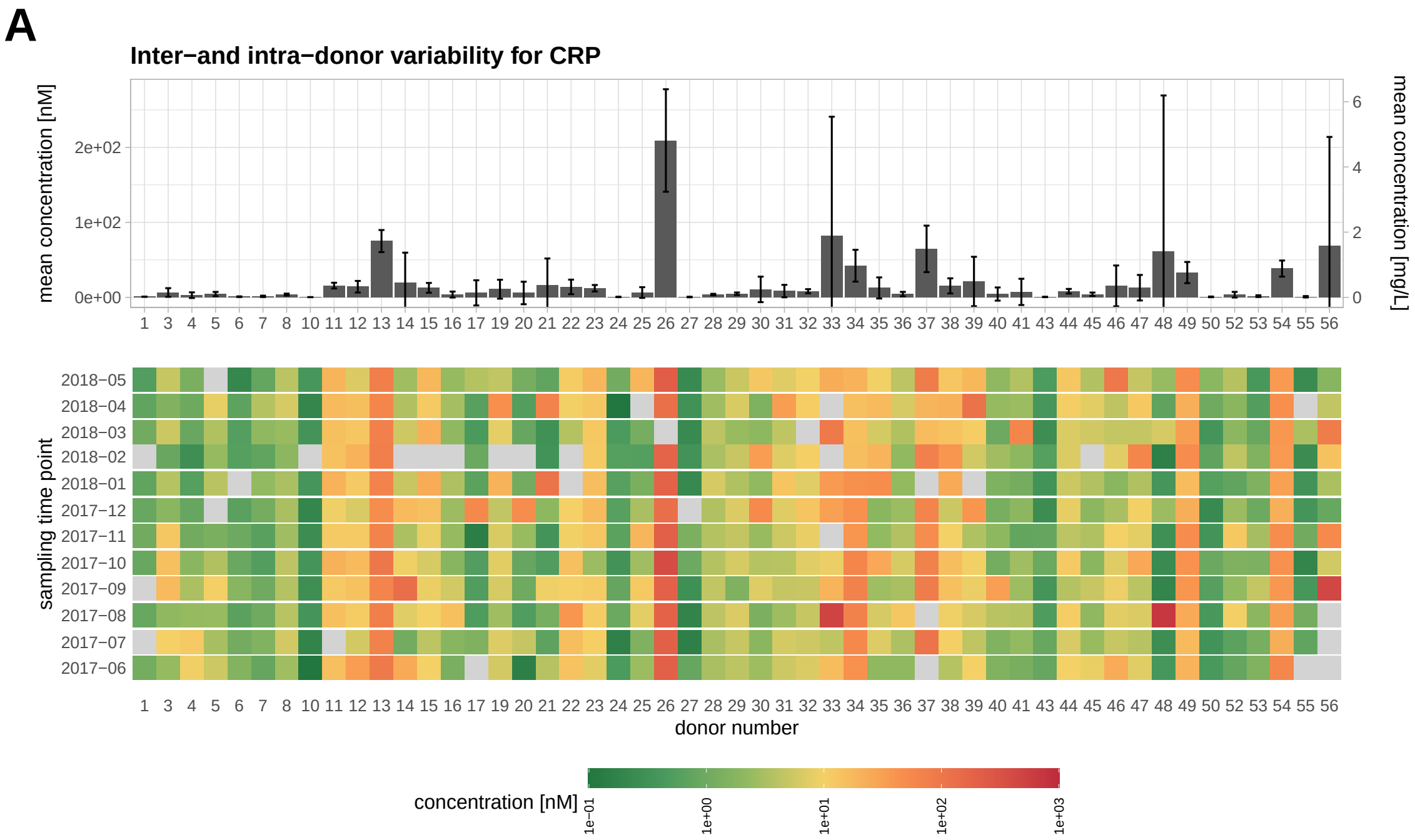

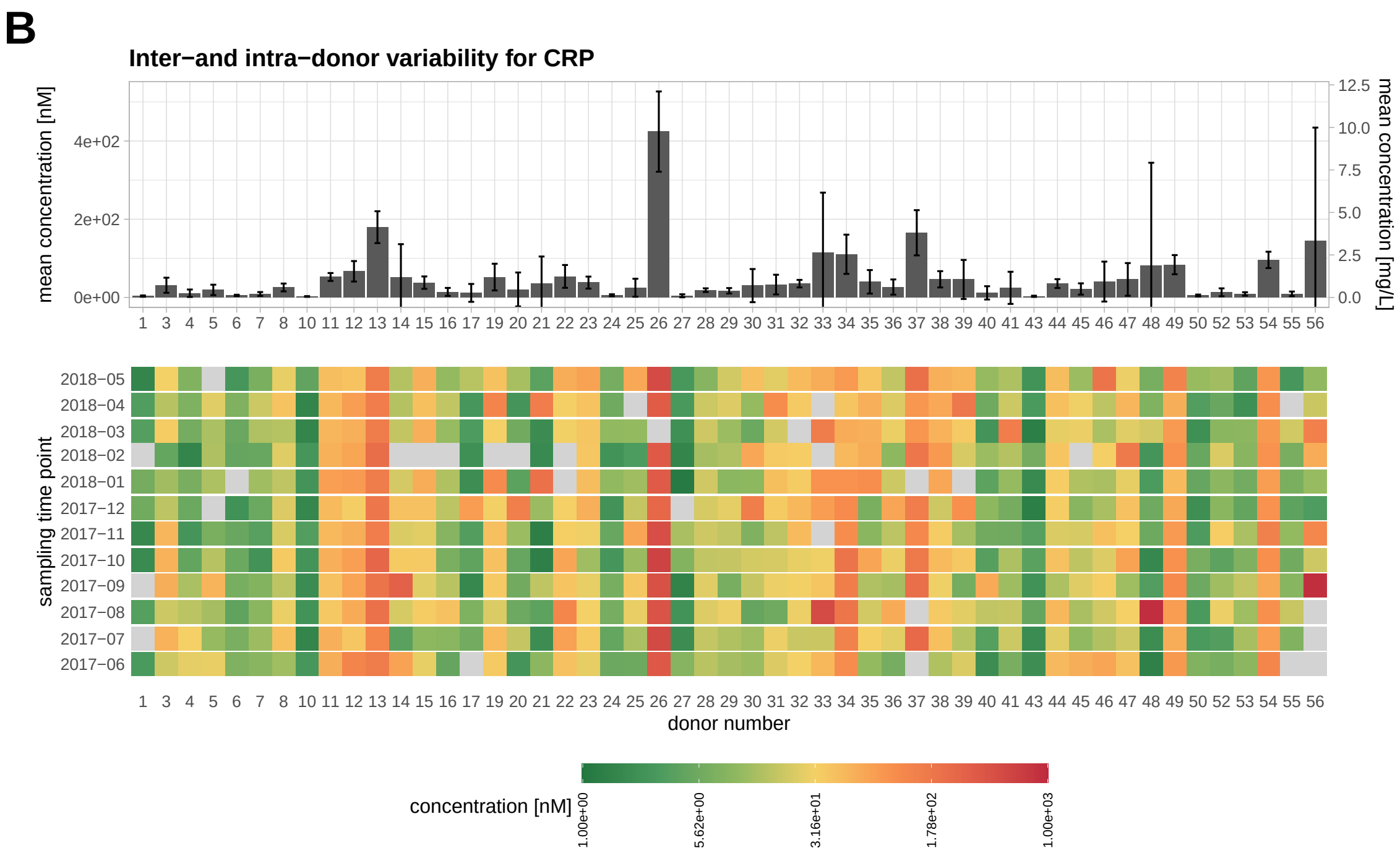

Appendix Figure S16: CRP - A: Utrecht site, B: Greifswald site - Mean concentration shown in barplot (top panel). The tile plot below (lower panel) depicts the temporal profiles of CRP in donors (grey color represent missing values). Color scale boundaries were defined using log10-transformed donor specific averaged nM estimated values. The lower bound was set at the 1st percentile, and the upper bound at the 99th percentile.

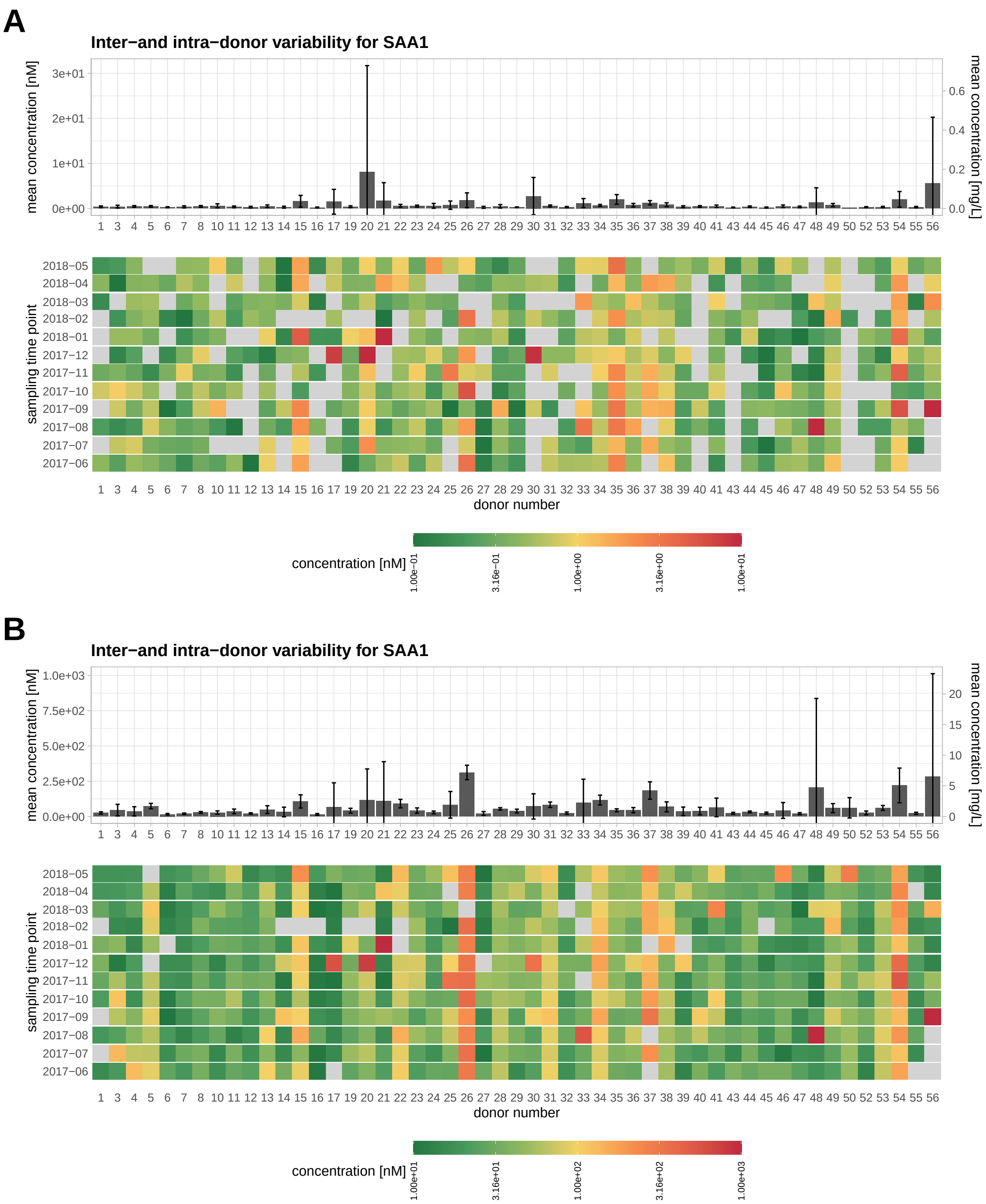

Appendix Figure S17: SAA1 - A: Utrecht site, B: Greifswald site - Mean concentration shown in barplot (top panel). The tile plot below (lower panel) depicts the temporal profiles of SAA1 in donors (grey color represent missing values). Color scale boundaries were defined using log10-transformed donor specific averaged nM estimated values. The lower bound was set at the 1st percentile, and the upper bound at the 99th percentile.

A

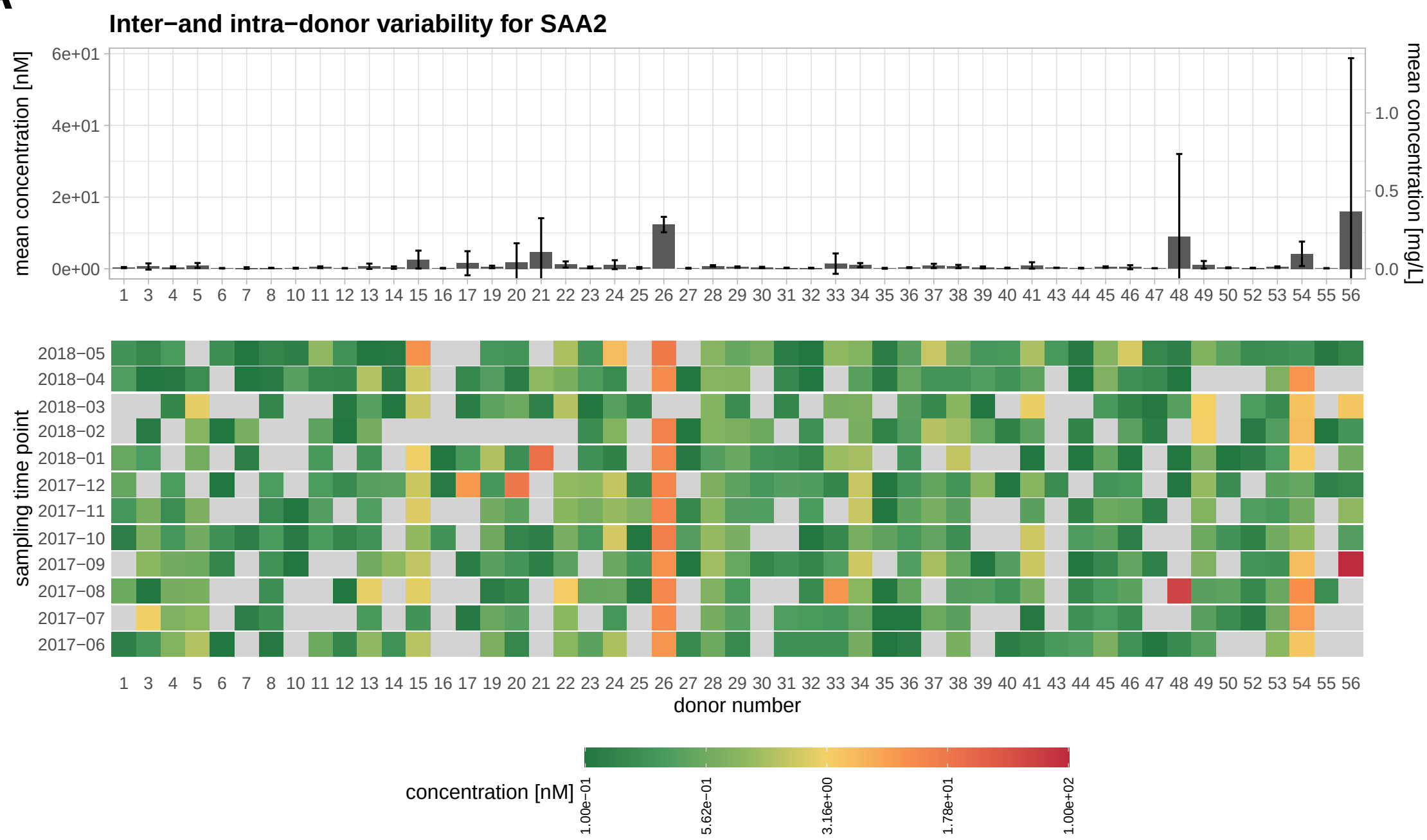

B

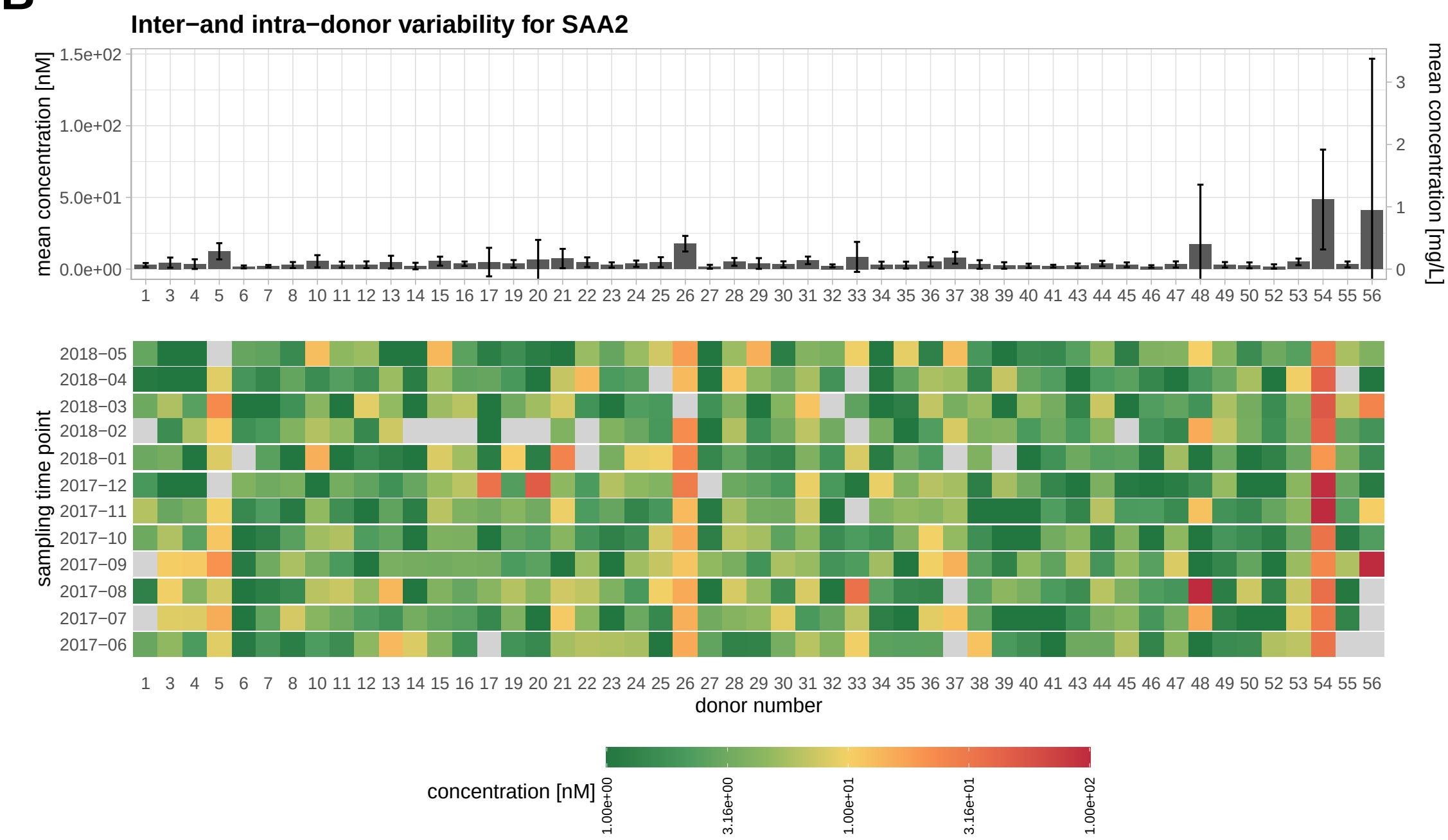

Appendix Figure S18: SAA2 - A: Utrecht site, B: Greifswald site - Mean concentration shown in barplot (top panel). The tile plot below (lower panel) depicts the temporal profiles of SAA2 in donors (grey color represent missing values). Color scale boundaries were defined using log10-transformed donor specific averaged nM estimated values. The lower bound was set at the 1st percentile, and the upper bound at the 99th percentile.

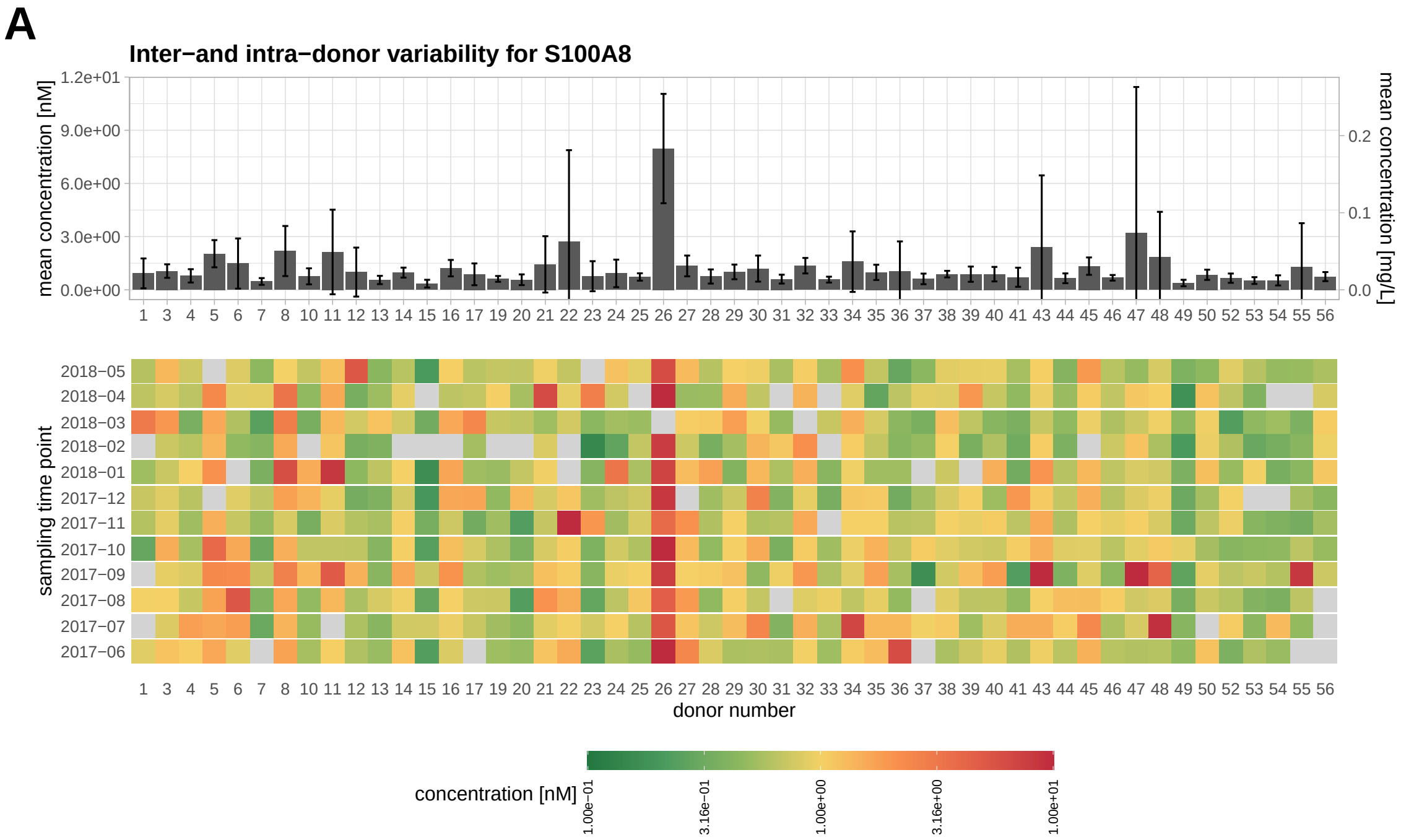

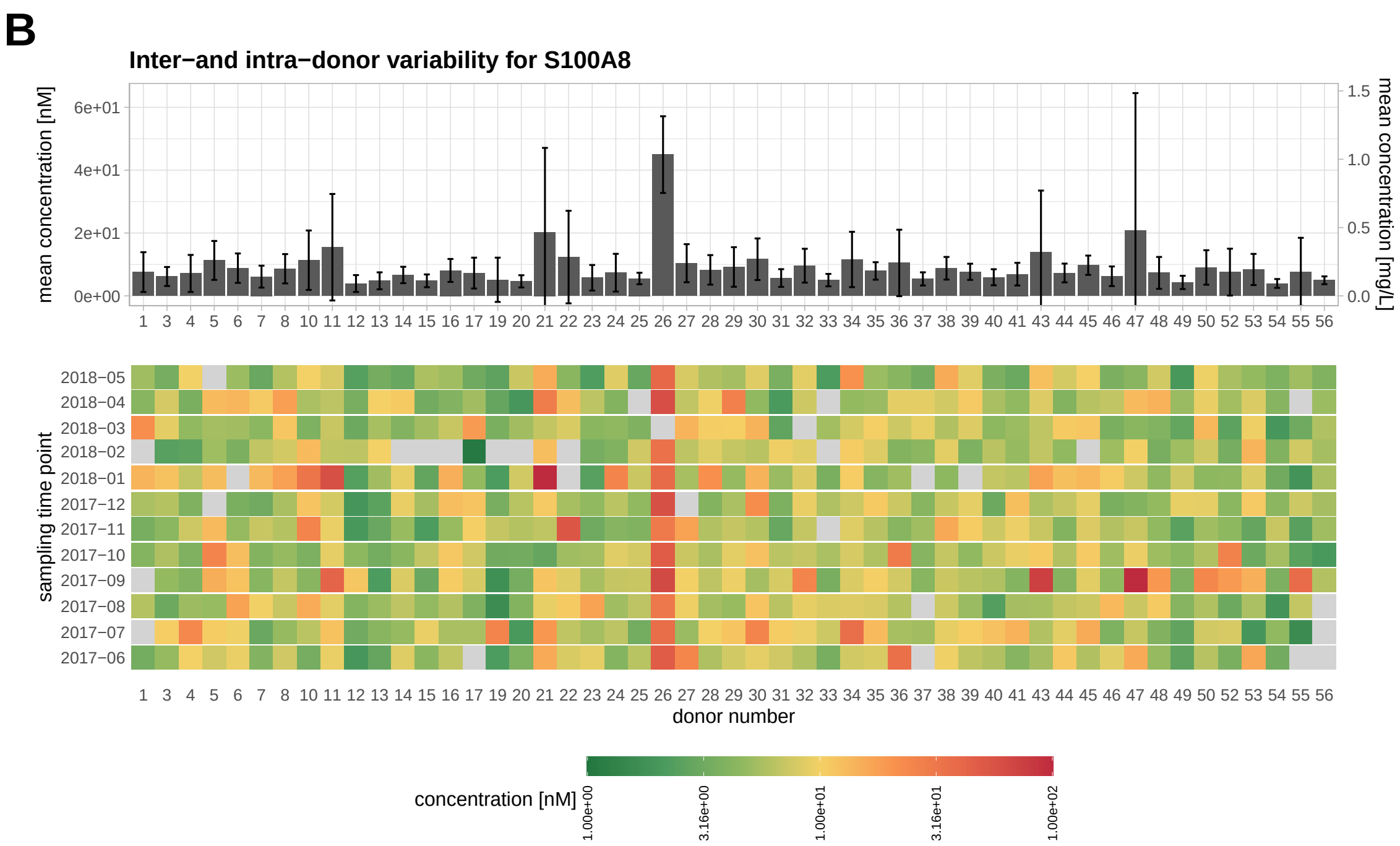

Appendix Figure S19: S100A8 - A: Utrecht site, B: Greifswald site - Mean concentration shown in barplot (top panel). The tile plot below (lower panel) depicts the temporal profiles of S100A8 in donors (grey color represent missing values). Color scale boundaries were defined using log10-transformed donor specific averaged nM estimated values. The lower bound was set at the 1st percentile, and the upper bound at the 99th percentile.

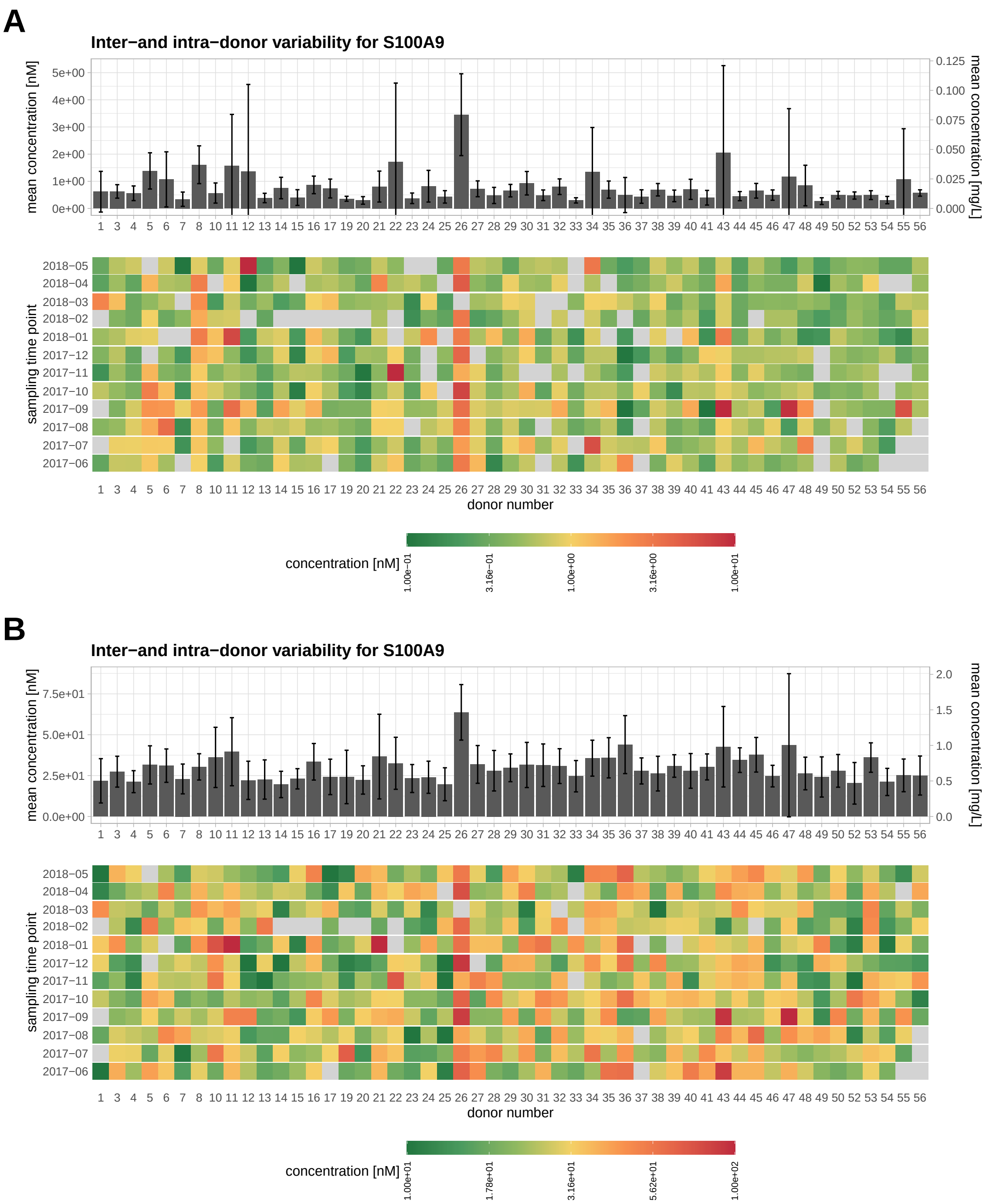

Appendix Figure S20: S100A9 - A: Utrecht site, B: Greifswald site - Mean concentration shown in barplot (top panel). The tile plot below (lower panel) depicts the temporal profiles of S100A9 in donors (grey color represent missing values). Color scale boundaries were defined using log10-transformed donor specific averaged nM estimated values. The lower bound was set at the 1st percentile, and the upper bound at the 99th percentile.

Supplementary Methods

TIMES cohort - study design

The TIMES study aimed to investigate the annual variations in the plasma proteome of healthy individuals. Eligibility criteria included being between 18 and 60 years old, providing written informed consent, and not having any acute illness within the past seven days before sampling. Exclusion criteria included a body mass index below 18.5 kg/m², as well as the presence of blood coagulation disorders, anemia, or similar conditions. Plasma samples were collected from a total of 53 participants (with a mean age of 34.4 ± 10.0 years) once a month for a year, spanning from June 2017 to May 2018. Due to early loss to follow-up, two subjects were excluded from the data analyses. Consequently, data from the remaining 51 participants were included in the analyses. A table with phenotyping data of donors can be find below (Appendix Table S2).

Appendix Table S2: TIMES cohort overview

	Overall (N=51)
**Sex**	
- female	32 (62.7%)
- male	19 (37.3%)
**Age (years)**	
- Mean (SD)	34.33 (9.96)
- Range	19.00 - 56.00
**weight (kg)**	
- Mean (SD)	74.81 (18.76)
- Range	51.00 - 145.00
**size (cm)**	
- Mean (SD)	173.61 (10.97)
- Range	151.00 - 198.00
**sample count per donor**	
- Mean (SD)	11.39 (0.92)
- Range	9.00 - 12.00
**BMI**	
- Mean (SD)	24.58 (4.36)
- Range	18.40 - 39.80

timsTOF - diaPASEF settings

diaPASEF settings timsTOF Pro 2 - Greifswald site

Appendix Table S3: diaPASEF windows timsTOF Pro 2

cycle ID	1/K0 start	1/K0 end	m/z start	m/z end
1	0.67	0.85	330.17	415.23
1	0.85	1.12	688.69	728.86
2	0.70	0.90	413.23	485.01
2	0.90	1.15	726.86	768.90
3	0.72	0.93	483.01	528.94
3	0.93	1.19	766.90	813.39
4	0.74	0.96	526.94	561.75
4	0.96	1.23	811.39	861.41
5	0.76	0.99	559.75	595.00
5	0.99	1.28	859.41	923.50
6	0.78	1.02	593.00	625.33
6	1.02	1.32	921.50	1001.24
7	0.79	1.05	623.33	656.11
7	1.05	1.39	999.24	1107.20
8	0.80	1.08	654.11	690.69
8	1.08	1.47	1105.20	1638.48

diaPASEF settings timsTOF HT - Utrecht site

Appendix Table S4: diaPASEF windows timsTOF HT

cycle ID	1/K0 start	1/K0 end	m/z start	m/z end
1	0.600	0.832	301.84	407.88
1	0.832	1.600	621.80	641.85
2	0.600	0.862	407.88	436.22
2	0.862	1.600	641.85	662.35
3	0.600	0.872	436.22	458.25
3	0.872	1.600	662.35	683.84
4	0.600	0.892	458.25	478.22
4	0.892	1.600	683.84	707.82
5	0.600	0.902	478.22	496.74

(continued)

cycle ID	1/K0 start	1/K0 end	m/z start	m/z end
5	0.902	1.600	707.82	732.86
6	0.600	0.912	496.74	514.77
6	0.912	1.600	732.86	761.38
7	0.600	0.922	514.77	531.80
7	0.922	1.600	761.38	792.34
8	0.600	0.942	531.80	549.29
8	0.942	1.600	792.34	827.87
9	0.600	0.952	549.29	566.80
9	0.952	1.600	827.87	869.93
10	0.600	0.972	566.80	584.62
10	0.972	1.600	869.93	924.00
11	0.600	1.002	584.62	602.33
11	1.002	1.600	924.00	1001.48
12	0.600	1.052	602.33	621.80
12	1.052	1.600	1001.48	1199.55

Spectronaut® settings

The raw data analysis at the Greifswald site was conducted using Spectronaut® (version 20.1.250624.92449) in directDIA+ mode. The analysis utilized reviewed uniprot databases, specifically the *Homo sapiens* database, which contains 42,529 canonical and isoform entries. A detailed list of all parameters used is provided in the table below (Appendix Table S5).

Appendix Table S5: Spectronaut® settings

Level 1	Level 2	Level 3	Level 4	Value
Calibration	Calibration Mode			Automatic
Calibration	MZ Extraction Strategy			Maximum Intensity
Calibration	Used Biognosys' iRT Kit			FALSE
Calibration	Allow source specific iRT Calibration			TRUE
Calibration	Precision iRT	Exclude De-amidated Peptides		TRUE
Calibration	Precision iRT	iRT <-> RT Regression Type		Local (Non-Linear) Regression
Calibration	Calibration Carry-Over			FALSE
Calibration	MS1 Mass Tolerance Strategy			System Default
Calibration	MS2 Mass Tolerance Strategy			System Default
Identification	Precursor Qvalue Cutoff			0.01
Identification	Precursor Qvalue Cutoff (Experiment)			0.01
Identification	Precursor PEP Cutoff			0.2
Identification	Protein Qvalue Cutoff (Experiment)			0.01
Identification	Protein Qvalue Cutoff (Run)			0.05
Identification	Protein PEP Cutoff			0.75
Identification	Single Hit Definition			By Stripped Sequence
Identification	Single Hit Protein Rule			Stratified Single Hit Protein FDR
Identification	Run-Level Protein Scoring			All Observations
Identification	Exclude Duplicate Assays			TRUE

(continued)

Level 1	Level 2	Level 3	Level 4	Value
Identification	Exclude Predicted Fragment Scores			FALSE
Identification	Generate Decoys	Decoy Generation Method	Preferred Fragment Source	NN Predicted Fragments
Identification	Generate Decoys	Decoy Limit Strategy	Library Size Fraction	0.1
Identification	Pvalue Estimator			Kernel Density Estimator
Protein Inference	Protein Inference Workflow	Inference Algorithm		IDPicker
Quantification	Precursor Filtering	Imputation Strategy		Use Background Signal
Quantification	Precursor Filtering	Multi Channel Qvalue Filter		Group Qvalue
Quantification	Proteotypicity Filter			None
Quantification	Protein LFQ Method			Automatic
Quantification	Quantity MS Level			MS2
Quantification	Quantity Type			Area
Quantification	Cross-Run Normalization	Normalization Filter Type		None
Quantification	Cross-Run Normalization	Normalization Strategy		Automatic
Quantification	Cross-Run Normalization	Row Selection		Automatic
Quantification	Perform background noise removal			TRUE
Quantification	Quantification window			Not Synchronized (SN 17)
Quantification	Interference Correction	Only Identified Peptides		TRUE
Quantification	Interference Correction	Exclude All Multi-Channel Interferences		TRUE
Quantification	Interference Correction	MS1 Min		2
Quantification	Interference Correction	MS2 Min		3
Quantification	Major Group Quantity			Mean peptide quantity
Quantification	Minor (Peptide) Grouping			by Stripped Sequence
Quantification	Major (Protein) Grouping			by Protein Group Id
Quantification	Major Group Top N	Max		3
Quantification	Major Group Top N	Min		1
Quantification	Minor Group Quantity			Mean precursor quantity
Quantification	Minor Group Top N	Max		3
Quantification	Minor Group Top N	Min		1
Quantification	Use Log2 Quantity Filter			FALSE
Quantification	Perform IM Peak Picking for Quantification			TRUE
Workflow	Multi-Channel Workflow Definition	Fallback Option		Labeled
Workflow	Profiling Strategy			None
Workflow	Hybrid (DDA + DIA) Library			FALSE
Workflow	In-Silico Library Optimization			FALSE

(continued)

Level 1	Level 2	Level 3	Level 4	Value
Workflow	Unify Peptide Peaks Strategy			None
XIC Extraction	XIC IM Extraction Window	Correction Factor		1
XIC Extraction	XIC RT Extraction Window	Correction Factor		1
XIC Extraction	MS1 Mass Tolerance Strategy	Tolerance (ppm)		7
XIC Extraction	MS2 Mass Tolerance Strategy	Tolerance (ppm)		7

DIA-NN settings

The raw data analysis at the Utrecht site was conducted using DIA-NN (version 1.9). The analysis utilized and in-house blood protein database, that includes the SwissProt *Homo sapiens* sequences of the 2,445 proteins that are found in blood quantified, by either ELISA or MS, in blood according to the Human Protein Atlas (acquired November 13 2023). A detailed list of all parameters used is provided in the table below (Appendix Table S6).

Appendix Table S6: DIA-NN settings

parameter	value
Cross-run normalization	RT-dependent
Deep learning-based spectra, RTs and Ims prediction	enabled
FASTA digest for library-free search	enabled
Fixed modifications	N-term M excision, C carbamidomethylation
Fragment ion m/z range	200-1800
Heuristic protein inference	disabled
Library generation	Ids, RT & IM profiling
Mass accuracy	20
Maximum number of variable modifications	1
MBR	enabled
Missed cleavages	2
MS1 accuracy	10
Neural network classifier	Single-pass mode
No shared spectra	enabled
Peptide length range	7-32
Peptideoforms	enabled
Precursor charge range	1-4
Precursor m/z range	300-1800
Protein inference	Genes
Quantification strategy	Legacy (direct)
Threads	40
Unrelated runs	enabled
Variable modifications	Oxidation (M)

References

Gaither C, Popp R, Mohammed Y & Borchers CH (2020) Determination of the concentration range for 267 proteins from 21 lots of commercial human plasma using highly multiplexed multiple reaction monitoring mass spectrometry. *Analyst* 145: 3634–3644

Geyer PE, Voytik E, Treit PV, Doll S, Kleinhempel A, Niu L, Müller JB, Buchholtz M, Bader JM, Teupser D, *et al* (2019) Plasma proteome profiling to detect and avoid sample-related biases in biomarker studies. *EMBO Molecular Medicine* 11: e10427
