# Supplementary material for: Immunoglobulin sub-class levels define inter-donor plasma variability: a longitudinal dual-lab study
Source: Mol Syst Biol. 2026 May 26;22(8):1312–32. doi: 10.1038/s44320-026-00218-5 (PMC13434392; doi:10.1038/s44320-026-00218-5)
Supplement: Supplementary file 2 [file 44320_2026_218_MOESM2_ESM.pdf]

molecular systems biology

Immunoglobulin sub-class levels define inter-donor plasma variability: a longitudinal dual-lab study

Stephan Michalik, Sofia Kalaidopoulou Nteak, Nicolas Drouin, Manuela Gesell-Salazar, Elke Hammer, Vishnu Dhople, Silva Holtfreter, Stefan Weiss, Henk W P van den Toorn, Barbara Bröker, Grazyna Domanska, Uwe Völker, and Albert Heck

Corresponding author(s): Albert Heck (a.j.r.heck@uu.nl) , Uwe Völker (voelker@uni-greifswald.de)

Review Timeline:

Submission Date:	5th Nov 25
Editorial Decision:	5th Dec 25
Revision Received:	5th Mar 26
Editorial Decision:	31st Mar 26
Revision Received:	30th Apr 26
Accepted:	8th May 26

Editor: Jingyi Hou

Transaction Report:

(Note: With the exception of the correction of typographical or spelling errors that could be a source of ambiguity, letters and reports are not edited. Depending on transfer agreements, referee reports obtained elsewhere may or may not be included in this compilation. Referee reports are anonymous unless the Referee chooses to sign their reports.)

5th Dec 2025

Manuscript Number: MSB-2025-13450
Title: Immunoglobulin sub-class levels define inter-donor plasma variability: a longitudinal dual-lab study
Author: Stephan Michalik
Sofia Kalaidopoulou Nteak
Nicolas Drouin
Manuela Gesell-Salazar
Elke Hammer
Vishnu Dhople
Silva Holtfreter
Stefan Weiss
Henk W P van den Toorn
Barbara Bröker
Grazyna Domanska
Uwe Völker
Albert Heck

Dear Albert,

Thank you for submitting your work to Molecular Systems Biology. We have now received evaluations from three reviewers who kindly agreed to assess your manuscript.

As you will see from their comments below, Reviewers #1 and #2 consider the manuscript to be of interest and are generally supportive. However, Reviewer #3 has raised significant concerns. Given the overall balance of the reviewer reports, we are willing to offer you the opportunity to revise the manuscript, provided that all technical and analytical issues raised by the reviewers are thoroughly addressed.

Additionally, in response to Reviewer #3's comments on conceptual novelty, we ask that you more clearly articulate the new insights your study offers and explain how these insights could guide future research in the field. Reviewer #1 has also provided an annotated version of the manuscript with suggestions, which is attached here for your reference. Please take these suggestions into careful consideration during your revision.

As you may already know, our editorial policy allows in principle a single round of major revision so it is essential to provide responses to the reviewers' comments that are as complete as possible. Please feel free to contact me in case you would like to discuss in further detail any of the issues raised by the reviewers.

On a more editorial level, we would ask you to address the following issues:

- Please provide a .docx formatted version of the manuscript text (including legends for main figures, EV figures and tables). Please make sure that the changes are highlighted to be clearly visible.

- Please provide individual production quality figure files as .eps, .tif, .jpg (one file per figure). When assembling figures, please refer to our figure preparation guideline in order to ensure proper formatting and readability in print as well as on screen: https://media.springernature.com/original/springer-cms/rest/v1/content/27825798/data/v1

-Please provide a .docx formatted letter INCLUDING the reviewers' reports and your detailed point-by-point responses to their comments. As part of the EMBO Press transparent editorial process, the point-by-point response is part of the Review Process File (RPF), which will be published alongside your paper.

-Please note that all corresponding authors are required to supply an ORCID ID for their name upon submission of a revised manuscript.

-We replaced Supplementary Information with Expanded View (EV) Figures and Tables that are collapsible/expandable online. A maximum of 5 EV Figures can be typeset. EV Figures should be cited as 'Figure EV1, Figure EV2" etc... in the text and their respective legends should be included in the main text after the legends of regular figures.

Additional Tables/Datasets should be labeled and referred to as Table EV1, Dataset EV1, etc. Legends have to be provided in a separate tab in case of .xls files. Alternatively, the legend can be supplied as a separate text file (README) and zipped together with the Table/Dataset file.

For the figures and tables that you do NOT wish to display as Expanded View figures, they should be bundled together with their legends in a single PDF file called *Appendix*, which should start with a short Table of Content. Each legend should be below the corresponding Figure/Table in the Appendix. Appendix figures and tables should be referred to in the main text as: "Appendix Figure S1, Appendix Figure S2, Appendix Table S1" etc. See detailed instructions regarding expanded view here: https://link.springer.com/journal/44320/submission-guidelines#cms-Expanded-View-data.

-Before submitting your revision, primary datasets (and computer code, where appropriate) produced in this study need to be deposited in an appropriate public database.

Please remember to provide a reviewer password if the datasets are not yet public.
The accession numbers and database should be listed in a formal "Data Availability" section (placed after Materials & Method) that follows the model below (see also https://link.springer.com/partners/embo-press/editorial-policies#Data%20availability%20statement). Please note that the Data Availability Section is restricted to new primary data that are part of this study.
Data availability
The datasets (and computer code) produced in this study are available in the following databases:
- RNA-Seq data: Gene Expression Omnibus GSE46843 (https://www.ncbi.nlm.nih.gov/geo/query/acc.cgi?acc=GSE46843)
- [data type]: [name of the resource] [accession number/identifier/doi] ([URL or identifiers.org/DATABASE:ACCESSION])
*** Note - All links should resolve to a page where the data can be accessed. ***

- At EMBO Press we ask authors to provide source data for the main manuscript figures. You will receive a separate email with instructions for providing source data with your revised manuscript, including how to upload and organize the files.
Additional information on source data and instruction on how to label the files are available < https://link.springer.com/journal/44320/submission-guidelines#cms-Source-data >

- Our journal encourages inclusion of *data citations in the reference list* to directly cite datasets that were re-used and obtained from public databases. Data citations in the article text are distinct from normal bibliographical citations and should directly link to the database records from which the data can be accessed. In the main text, data citations are formatted as follows: "Data ref: Smith et al, 2001". In the Reference list, data citations must be labeled with "[DATASET]". A data reference must provide the database name, accession number/identifiers and a resolvable link to the landing page from which the data can be accessed at the end of the reference. Further instructions are available at < https://link.springer.com/journal/44320/submission-guidelines#cms-Reference-guidelines >.

- We updated our journal's competing interests policy in January 2022 and request authors to consider both actual and perceived competing interests. Please review the policy (https://link.springer.com/partners/embo-press/editorial-policies#Competing%20interest%20disclosures) and update your competing interests if necessary.
Please use the heading "Disclosure statement and competing interests".

- All Materials and Methods need to be described in the main text using our 'Structured Methods' format. According to this format, the Methods section includes a Reagents and Tools Table (listing key reagents, experimental models, software and relevant equipment and including their sources and relevant identifiers) followed by a Methods and Protocols section describing the methods, ideally using a step-by-step protocol format. The aim is to facilitate adoption of the methodologies across labs.

Please download and fill our Reagents and Tools Table template (.docx), which you can find in our author guidelines: https://link.springer.com/journal/44320/submission-guidelines#structuredmethods.

When submitting your revised manuscript, please do not include the Reagents and Tools Table in the Methods section of the manuscript but upload it as a separate file choosing the file type "Reagent Table".

-Regarding data quantification:
Please ensure to specify the name of the statistical test used to generate error bars and P values, the number (n) of independent experiments (please specify technical or biological replicates) underlying each data point and the test used to calculate p-values in each figure legend. Discussion of statistical methodology can be reported in the materials and methods section, but figure legends should contain a basic description of n, P and the test applied.
Graphs must include a description of the bars and the error bars (s.d., s.e.m.).
Please also include scale bars in all microscopy images.

- Please provide a "standfirst text" summarizing the study in one or two sentences (approximately 250 characters, including space), three to four "bullet points" highlighting the main findings and a "synopsis image" (550px width and 400-600 px height, PNG format) to highlight the paper on our homepage.
Please refer to published papers for examples.

When you resubmit your manuscript, please download our CHECKLIST (https://media.springernature.com/original/springer-cms/rest/v1/content/27825796/data/v1) and include the completed form in your submission.

Please note that the Author Checklist will be published alongside the paper as part of the transparent process .

If you feel you can satisfactorily deal with these points and those listed by the referees, you may wish to submit a revised version of your manuscript. Please attach a covering letter giving details of the way in which you have handled each of the points raised by the referees. A revised manuscript will be once again subject to review and you probably understand that we can give you no guarantee at this stage that the eventual outcome will be favorable.

I look forward to receiving the revised manuscript soon.

Kind regards,
Jingyi

Jingyi Hou, PhD
Senior Editor
Molecular Systems Biology

We realize that it is difficult to revise to a specific deadline. In the interest of protecting the conceptual advance provided by the work, we recommend a revision within 3 months (5th Mar 2026). Please discuss the revision progress ahead of this time with the editor if you require more time to complete the revisions. Use the link below to submit your revision:

https://msb.msubmit.net/cgi-bin/main.plex

*** PLEASE NOTE *** As part of the EMBO Press transparent editorial process initiative (see our Editorial at https://dx.doi.org/10.1038/msb.2010.72), Molecular Systems Biology publishes online a Review Process File with each accepted manuscripts. This file will be published in conjunction with your paper and will include the anonymous referee reports, your point-by-point response and all pertinent correspondence relating to the manuscript. If you do NOT want this File to be published, please inform the editorial office at contact@molsystbiol.org within 14 days upon receipt of the present letter.

Reviewer #1:

Minor revision.
This is a very nice study. My only suggestion would be to include references such as PMID: 38655860, where the authors also found that the blood proteome was very stable and specific to an individual.
I also wholeheartedly agree with the author's statement about the importance of using internal standards, altho I would change it to "spiked-in isotopically labelled internal standards.":
Line 549-552:
"Although averaging 548 across proteins and adjusting the mean values could facilitate such comparisons, the 549 inter-laboratory evaluation results strongly support including a universal plasma 550 reference sample or using spiked-in standards with each measurement workflow (Cai 551 et al, 2025) since cohorts and disease phenotype plasma proteome can be highly variable."
Other minor changes are shown on the annotated pdf.

Reviewer #2:

Michalik et al. reported a comprehensive and rigorously conducted longitudinal study of the human plasma proteome. The dual lab design is a particular strength, providing a valuable empirical assessment of cross-site reproducibility in clinical proteomics. The central finding that immunoglobulin levels are a major source of stable and individual-specific proteomic signatures, while inflammation markers show high temporal variability is well-supported by the data. The study makes a compelling case for the importance of longitudinal sampling in biomarker discovery. The manuscript is generally well-written, and the experimental design is sound. However, several points require clarification and discussion to strengthen the interpretation of the findings.

The authors explicitly state they focused on a core set of ~200 consistently quantified proteins to ensure robustness, acknowledging that deeper proteome coverage was not the goal. While this is a valid approach for a reproducibility study, it necessitates a clearer justification. The reader is left wondering how the conclusions might change if a larger, more dynamic set of low-abundance proteins were included. A brief discussion on the potential biological insights that may be missed by excluding

this larger range of proteome, and how the balance between robustness and breadth was struck, would be beneficial.

The protein-wise normalization method employed (averaging concentrations per site and calculating a ratio) is pragmatic but raises a key question: whether this adjustment may mask differences in protein quantification between the workflows. The method essentially forces the average concentration for each protein to be equal between sites. A more detailed description or a positive control (e.g., using a set of spike-in standards) to validate that this method accurately recovers true biological concentrations, rather than just improving correlation, would significantly strengthen the claims about quantitation.

The discussion should more deeply contextualize the intra-individual stability of immunoglobulins. A comment on how this stability might relate to the dynamic nature of the adaptive immune response would provide a more nuanced view.

The discussion of the SP3 vs. liquid digestion methods is informative. However, it would be useful to briefly speculate on which specific aspects of the differing workflows (database size, digestion protocol, or MS instrument) contributed most to the variation in the number of proteins identified before overlap. Was the larger database in Greifswald the primary driver, or were sensitivity and loading amount more critical?

The limitations section is appropriate but could be expanded. In addition to cohort size and depth, the authors should mention the health status of the cohort. The finding of donors with consistently elevated basal CRP levels suggests possible subclinical conditions. A discussion on how undiagnosed morbidity in a "supposedly healthy" cohort might influence the interpretation of "stable" vs. "variable" proteins would be prudent.

Reviewer #3:

This manuscript reports a longitudinal, dual-site plasma proteomics study (TIMES) following healthy donors monthly for one year, with a focus on cross-laboratory harmonization and on immunoglobulin subclasses as key contributors to inter-donor variability. The experimental work appears carefully conducted, and the cohort design and analytical pipelines are described in substantial technical detail. The dataset is potentially valuable as a reference resource. However, in its current form the study is largely descriptive and confirms several expected observations (e.g. low intra-individual vs high inter-individual variability, stable immunoglobulin levels, episodic CRP spikes) without providing substantial mechanistic or systems-level insight. The "dual-lab" design is only partially exploited, as most biological conclusions are derived from one site's data. In addition, important questions about cohort design, generalizability, cross-site harmonization, and pre-analytical bias remain insufficiently addressed. Together, the work does not yet reach the level of conceptual advance and integrative systems biology expected for Molecular Systems Biology; I therefore recommend rejection, while noting that the study could be suitable for a more technically oriented proteomics journal after revision.

Major concerns
1. Limited conceptual novelty and largely descriptive nature of the study.The main biological findings: the intra-individual plasma proteomes are highly stable over one year; the inter-individual differences dominate; and the immunoglobulin subclasses and CRP explain much of this variation,are interesting but largely confirm prior work and align with intuitive expectations. To reach the bar of Molecular Systems Biology, the study would benefit from deeper systems-level analyses.
2. Cohort design and generalizability (age range, sex balance, covariates). The TIMES cohort is restricted to relatively young and middle-aged adults (reported inclusion 18-60 years, actual range 19-56 years, mean age ~34 years), with no representation of older individuals in whom immunosenescence, chronic inflammation, and comorbidities are more prevalent. Yet the manuscript makes broad statements about inter-individual variability in "human plasma proteomes" that may not generalize to older or less healthy populations. A clear justification for excluding older donors is needed, and claims about generalizability should be moderated or validated in an independent cohort. In addition, the sex distribution is imbalanced (32 females vs 19 males), but sex effects on immunoglobulin levels and inflammatory markers are not explored or adjusted for. Please consider (i) providing a more detailed description of recruitment, health status, medications, and lifestyle factors, and (ii) performing at least exploratory analyses testing whether age, sex, and BMI influence key readouts (IgG subclasses, CRP trajectories) or explicitly discussing this as a limitation.
3. Underuse of the dual-laboratory design and potential cross-site biases. A major strength is that ~600 samples were processed independently at two sites using distinct workflows and databases. Yet, after demonstrating strong correlations for a relatively small set of overlapping proteins (~163 after stringent filtering), the authors proceed to perform almost all subsequent biological interpretation on the Utrecht dataset only. This substantially weakens the impact of the "dual-lab" aspect and raises the question of whether the main conclusions are truly robust across workflows. It would be important either to (i) replicate key analyses with the Greifswald data (or a meta-analyzed dataset) and show concordance, or (ii) reposition the paper more explicitly as a primarily single-site biological analysis with a technical cross-site validation. Relatedly, the description of cross-site harmonization could be strengthened: Were sample aliquots randomized and processed in a blinded fashion at each lab? How was ongoing communication between teams managed to avoid unintentional tuning of methods to match each other? A more systematic description of cross-site QC (including any shared reference or spike-in materials) would help to demonstrate that human and protocol-induced biases are minimized.
4.Pre-analytical variation, storage effects, and the circannual objective. The study is framed as an investigation of "circannual

fluctuations" in the plasma proteome, yet pre-analytical factors (sample storage time and conditions, freeze-thaw cycles, shipment, acquisition order, instrument drift) are not thoroughly modeled or quantified. Although the authors describe standardized handling from phlebotomy to freezing, samples from different months will necessarily have different storage durations before MS analysis, and the two sites likely measured samples in different temporal sequences. It is not clear how the authors ensured that the observed stability of immunoglobulins and the variation in CRP and other markers are not confounded by storage or batch effects that are only partially addressed. More quantitative QC (e.g. performance of housekeeping proteins and/or spike-in standards over time, explicit modeling of storage duration and acquisition batch as covariates, and sensitivity analyses) would substantially strengthen the longitudinal claims. Clarifying whether each time point used a unique aliquot (single freeze-thaw) and whether a universal QC sample was run throughout the project would also be important.

Minor concerns

1. Alignment between Introduction and actual analyses (PTMs and glycosylation). The Introduction gives significant space to discussing post-translational modifications, especially glycosylation of immunoglobulins, as a contributor to plasma proteome complexity. However, the current analyses are limited to protein-level (or subclass-level) concentrations and do not address PTMs directly. Either include at least a limited analysis of relevant PTMs (e.g. IgG Fc glycosylation features that are accessible from the DIA data) or substantially tone down the PTM-focused narrative and clarify that PTMs are out of scope for this study.
2. Contextualization with UK Biobank and other large proteomics resources. To better position the TIMES cohort, it would be helpful to compare key distributions (e.g. total IgG and subclass concentrations, CRP baseline levels, frequency of high-CRP outliers) with those observed in large-scale resources such as the UK Biobank proteomics datasets or other population-based plasma atlases. Even a qualitative comparison or a brief meta-analysis of published summary statistics would help to assess whether the strong inter-individual differences seen here are typical and to what extent this relatively small cohort reflects broader populations.
3. Cohort description and covariate analyses.The manuscript would benefit from a more detailed description of the cohort and sampling framework: e.g. recruitment source (blood donors vs general population), frequency of blood donation, smoking status, metabolic status, and any exclusion of chronic diseases or medications that may influence immunoglobulin or CRP levels. Since the BMI range is wide (18.4-39.8 kg/m²), and several donors appear to have chronically elevated CRP, it would be informative to report whether BMI or other available covariates correlate with immunoglobulin profiles or inflammatory markers, even at a descriptive level.
4. Clarification of immunoglobulin peptide handling across workflows. For the cross-site comparison, proteins "containing variable immunoglobulin regions" were removed, while the main biological analyses explicitly rely on immunoglobulin subclass quantification. It would be useful to explain more clearly which immunoglobulin peptides (constant vs variable regions) were used in each pipeline, how subclass-specific peptides were defined, and whether immunoglobulin measurements are directly comparable between sites. This is particularly important given the different FASTA/databases used (compact blood-specific vs large UniProt with isoforms and manually added Ig sequences).
5. Quality control and sample identity checks. The detection of an apparently mislabelled/duplicated sample (December sample of donor 27 clustering with donor 4) illustrates the value of longitudinal design for QC. However, the criteria used to flag this sample as mislabelled (UMAP and clustering thresholds) should be described more systematically, and the authors should clarify whether other potential swaps or mix-ups were formally ruled out. In addition, please expand the description of QC procedures: were any stable isotope-labeled or protein spike-in controls used, and were pooled reference samples monitored across the entire acquisition to control for long-term instrument drift and plate-to-plate variation? Including summary QC plots (e.g. CVs of reference proteins/spike-ins over time) would be helpful.

This is the point-to-point response, with the reviewer's comments in black and your response in blue.

Reviewer #1:

Minor revision.
This is a very nice study. My only suggestion would be to include references such as PMID: 38655860, where the authors also found that the blood proteome was very stable and specific to an individual.
I also wholeheartedly agree with the author's statement about the importance of using internal standards, altho I would change it to "spiked-in isotopically labelled internal standards.": Line 549-552:
"Although averaging 548 across proteins and adjusting the mean values could facilitate such comparisons, the 549 inter-laboratory evaluation results strongly support including a universal plasma 550 reference sample or using spiked-in standards with each measurement workflow (Cai 551 et al, 2025) since cohorts and disease phenotype plasma proteome can be highly variable."
Other minor changes are shown on the annotated pdf.

We appreciate the very positive feedback and the comments made by the reviewer. We have now cited (Richard *et al*, 2024) in line 369. We also adjusted our text in line 625 to include the "spiked-in isotopically labelled internal standards." Furthermore, all minor changes from the annotated pdf file were implemented.

Reviewer #2:

Michalik et al. reported a comprehensive and rigorously conducted longitudinal study of the human plasma proteome. The dual lab design is a particular strength, providing a valuable empirical assessment of cross-site reproducibility in clinical proteomics. The central finding that immunoglobulin levels are a major source of stable and individual-specific proteomic signatures, while inflammation markers show high temporal variability is well-supported by the data. The study makes a compelling case for the importance of longitudinal sampling in biomarker discovery. The manuscript is generally well-written, and the experimental design is sound. However, several points require clarification and discussion to strengthen the interpretation of the findings.

We appreciate the positive comments made by the reviewer about the manuscript structure, the experimental design and the appreciation of the dual lab design providing a valuable empirical assessment of cross-site reproducibility in clinical proteomics.

1. The authors explicitly state they focused on a core set of ~200 consistently quantified proteins to ensure robustness, acknowledging that deeper proteome coverage was not the goal. While this is a valid approach for a reproducibility study, it necessitates a clearer justification. The reader is left wondering how the conclusions might change if a larger, more dynamic set of low-abundance proteins were included. A brief discussion on the potential biological insights that may be missed by excluding this larger range of proteome, and how the balance between robustness and breadth was struck, would be beneficial.

The reviewer makes a valuable comment in that deeper coverage of low abundant proteins could be beneficial. Our decision to focus on a core set of approximately 200 consistently quantified proteins was motivated by ensuring reproducibility and minimizing variability across biological and technical replicates. Previous studies have demonstrated that low-abundance proteins in DIA-MS analysis are inherently more susceptible to higher variability, due to missing values, higher FDR rates and lower S/N ratios, in label-free quantitative proteomics, which can compromise reproducibility (Distler *et al*, 2025).
Furthermore, the enhanced ion capacity and dynamic range of timsTOF HT (Utrecht) resulted in more reproducibly quantified precursors compared to timsTOF Pro 2 (Greifswald). The timsTOF HT yielded over 2-fold more reproducible precursors (triplicate LC−MS injections, CV < 20%) compared to timsTOF Pro 2 (Vitko *et al*, 2024).
Besides these technical reasons our study also stands out from most plasma proteomics studies by largely focusing on the immunoglobulins, very high abundant proteins in serum, but still often overlooked or ignored in other plasma proteomics studies. Often these immunoglobulins are even depleted from the serum to reach more depth in the proteome.
Still, as the reviewer makes a valuable point, we added in the revision a statement about the limited “depth” in the proteome in our study, in the now substantially extended “Limitations of the study” section (line 672-708).

2. The protein-wise normalization method employed (averaging concentrations per site and calculating a ratio) is pragmatic but raises a key question: whether this adjustment may mask differences in protein quantification between the workflows. The method essentially forces the average concentration for each protein to be equal between sites. A more detailed description or a positive control (e.g., using a set of spike-in standards) to validate that this method accurately recovers true biological concentrations, rather than just improving correlation, would significantly strengthen the claims about quantitation.

We thank the reviewer for the valuable comment. In response, we have removed the per-protein mean adjustment from the manuscript and revised figure 1. This normalization step was originally included with the intent of placing individual proteins on a comparable intensity scale across sites, without the goal of adjusting biological variation between samples or sites. However, we agree with the reviewer that this approach effectively forces the mean

concentration of each protein to be equal between sites, which could mask true quantitative differences between the two workflows and therefore risks overstating cross-site agreement.

In the revised manuscript, we now present the correlation analysis of the non-adjusted, non-normalized concentration data for both sites (revised figure 2), which provides a more transparent and unbiased assessment of cross-site quantitative agreement. The observed correlation in the non-adjusted data reflects the raw technical concordance between the two independent workflows without any scaling intervention, and shows that this correlation is still very high. We acknowledge that the absence of heavy labeled spike-in standards or shared reference materials prevents a definitive validation of absolute protein quantification accuracy across sites, and we have added this as an explicit limitation in the discussion (line 622-630).

3. The discussion should more deeply contextualize the intra-individual stability of immunoglobulins. A comment on how this stability might relate to the dynamic nature of the adaptive immune response would provide a more nuanced view.

The reviewer raises an intriguing point. Notably, we recently (Rijswijck *et al*, 2025) have shown that the majority of very high abundant antibodies in the plasma are generated by long-lived plasma cells likely residing in niches in the bone marrow. Also, the production and secretion levels of most of these high abundant antibodies are very stable over time in most donors not experiencing serious infection of other diseases states. We thank the reviewer for bringing this up and have added a statement and reference to this work in the discussion of the revised manuscript (line 643-648).

4. The discussion of the SP3 vs. liquid digestion methods is informative. However, it would be useful to briefly speculate on which specific aspects of the differing workflows (database size, digestion protocol, or MS instrument) contributed most to the variation in the number of proteins identified before overlap. Was the larger database in Greifswald the primary driver, or were sensitivity and loading amount more critical?

We thank the reviewer for this question. Disentangling the relative contributions of database size, digestion protocol, and instrument sensitivity to the observed differences in protein identification between the two sites is not straightforward, as these variables are inherently confounded in the current study design. Nevertheless, we would like to briefly discuss their likely contributions.

The larger spectral library and protein database employed in Greifswald likely contributed to the higher number of identified proteins at that site. Larger databases increase the likelihood of matching spectra to low-abundance proteins, although this comes at the expense of a potentially higher false discovery rate. However, database size alone is unlikely to fully explain the differences, as instrument sensitivity and data acquisition strategy are equally crucial in plasma proteomics. In this context, the timsTOF HT data acquisition used in Utrecht offers

relevant advantages. A recent systematic comparison by Vitko *et al.* (Vitko *et al*, 2024) demonstrated that the timsTOF Pro 2 and timsTOF HT platforms, which employ trapped ion mobility spectrometry combined with parallel accumulation serial fragmentation (PASEF), showed that the timsTOF HT outperforms the timsTOF Pro2. We've included a revised Figure 2 in the manuscript. It shows the scattered protein concentrations, which have a narrow dynamic range in the low-abundance protein range for the timsTOF Pro2. This highlights the superior quantitative performance of the timsTOF HT, which is further supported by a higher inter-donor CV for the timsTOF HT data for highly abundant proteins like albumin or transferrin (Appendix Figure S4-S5).
Regarding sample preparation and the comparison between SP3-based digestion used in Greifswald and in-solution digestion used in Utrecht: Varnavides *et al.* (Varnavides *et al*, 2022) performed a comprehensive survey of 16 bottom-up proteomics sample preparation protocols and found that SP3 yielded comparable overall protein identification depth to in-solution SDC-based digestion. However, SP3 exhibited lower cleavage efficiency with a slightly lower number of zero missed cleavage peptides, and a minor bead-driven extraction profile, resulting in subtle but consistent qualitative differences in the identified protein and peptide repertoire despite comparable overall coverage. This suggests that while the two digestion protocols perform similarly at the level of total protein identifications, they may subtly differ in the specific subset of proteins and peptides captured, which could contribute to the incomplete overlap observed between the two sites beyond what would be explained by database size or instrument sensitivity alone.

5. The limitations section is appropriate but could be expanded. In addition to cohort size and depth, the authors should mention the health status of the cohort. The finding of donors with consistently elevated basal CRP levels suggests possible subclinical conditions. A discussion on how undiagnosed morbidity in a "supposedly healthy" cohort might influence the interpretation of "stable" vs. "variable" proteins would be prudent.

Based on the comments of the reviewers we extended the limitation section substantially and addressed these concerns. We highlight donor 26 that exhibited a very high BMI, continuously high levels of CRP and consistently very low levels of the immunoglobulins IgG1, IgG2, IgG3, IgA1 and IgM, from which we now concluded that this is a clear outlier, and possibly should be considered as a not healthy donor (line 557-559 & 687-691).

Elevated baseline CRP, even in the absence of clinically overt disease, is often indicative of subclinical inflammatory or metabolic conditions that can modulate systemic protein expression and stability (Ridker *et al*, 2000; Pearson *et al*, 2003). This raises the possibility that the cohort, although nominally characterized as “healthy,” may include individuals with undiagnosed morbidity.
When immunoglobulin levels are consistently low (i.e. in the 2.5 % lower percentile) this is clinically often considered at risk.

The limitations section has been extended (line679-708):

Secondly, although the TIMES cohort was designed to recruit healthy donors, there is no independent clinical evidence for the absence of disease in all participants. For example, donor 26 is an outlier in terms of high BMI and body fat proportion (Figure EV1), very high basal levels of C-reactive protein (Figure 6) over the whole year, and very low levels of nearly all immunoglobulin subclasses, namely IgG1, IgG2, IgG3, IgA1, IgM (see Figure 5 and Appendix Figure S7, S9, S12, S14, S15). Clinically this is suggestive of common variable immunodeficiency (CVID), the most prevalent human primary immunodeficiency. The disease is usually diagnosed at around 30 years of age because of frequent and severe infections (Bonilla *et al*, 2015). However, donor 26 did not report increased susceptibility for infections. We conclude that her abnormally low immunoglobulin plasma levels might reflect immunodeficiency requiring further diagnosis. This chance finding highlights the potential value of diagnostic plasma proteomics.

Another limitation of the cohort may be that it does not include children/young adults and old people but inter- and intra-donor plasma protein variability may be affected by age. It is well known that immunoglobulin concentrations are gradually built-up during childhood and adolescence (Rich *et al*, 2018). In the elderly, on the other hand, the prevalence of subclinical and clinical health disorders increases. Thus, our conclusions mostly relate to the age group studied, namely between 19 and 56 years.

Moreover, we here focus solely on the ~200 plasma proteins that are consistently detected and can be robustly quantified over all donors and all time points. It has been shown that with more efforts the detectable plasma proteome can be increased to over 1000 proteins, where the gain comes mostly from lower abundant proteins. However, we did not aim for that as it would substantially increase the number of missing values, and thus also CVs and robustness in quantification. However, our study focuses largely on immunoglobulins, which are highly abundant plasma proteins. In the published large-scale in-depth plasma proteomics studies the immunoglobulins are often depleted from the plasma samples in order to reach a deeper depth in the proteome, whereas when not depleted they are often ignored in the subsequent data analysis, because of their extreme sequence variability.

Reviewer #3:

This manuscript reports a longitudinal, dual-site plasma proteomics study (TIMES) following healthy donors monthly for one year, with a focus on cross-laboratory harmonization and on immunoglobulin subclasses as key contributors to inter-donor variability. The experimental work appears carefully conducted, and the cohort design and analytical pipelines are described in substantial technical detail. The dataset is potentially valuable as a reference resource. However, in its current form the study is largely descriptive and confirms several expected observations (e.g. low intra-individual vs high inter-individual variability, stable immunoglobulin levels, episodic CRP spikes) without providing substantial mechanistic or systems-level insight. The "dual-lab" design is only partially exploited, as most biological conclusions are derived from one site's data. In addition, important questions about cohort design, generalizability, cross-site harmonization, and pre-analytical bias remain insufficiently addressed. Together, the work does

not yet reach the level of conceptual advance and integrative systems biology expected for Molecular Systems Biology; I therefore recommend rejection, while noting that the study could be suitable for a more technically oriented proteomics journal after revision.

We appreciate the reviewers view that the generated datasets are valuable as a reference resource, and have also taken the critique serious, by exploiting more the dual-lab design, adding more Utrecht and Greifswald data site-by-site, and discuss better the differences in design in the experimental workflows and how this affects the outcome. Moreover, by further highlighting the findings on the inter and intra-donor variability of immunoglobulins we also feel our study brings new system-level insight into key proteins in the plasma proteome often overlooked in other studies.

Major concerns

1. Limited conceptual novelty and largely descriptive nature of the study. The main biological findings: the intra-individual plasma proteomes are highly stable over one year; the inter-individual differences dominate; and the immunoglobulin subclasses and CRP explain much of this variation, are interesting but largely confirm prior work and align with intuitive expectations. To reach the bar of Molecular Systems Biology, the study would benefit from deeper systems-level analyses.

We respectfully disagree. For instance, considering the UK Biobank study "Large-scale plasma proteomics comparisons through genetics and disease associations. ***Nature*** 622, 348–358 (2023)" (Eldjarn *et al*, 2023) as a landmark plasma proteomics study it fully ignores the immunoglobulin levels. In detail, Olink does not provide comprehensive subclass- resolved immunoglobulin profiling (e.g. IgG1–4, IgA1/2) and acknowledges that for detailed immunoglobulin biology you would need dedicated LC-MS or ELISA assays. Also, another landmark paper in the field "A human pan-disease blood atlas of the circulating proteome" published in Science (Álvez *et al*, 2025) represent some of the most in-depth analysis across large cohorts of healthy and diseases donors, but also ignores immunoglobulin levels.
We find this surprising, as they do represent about 30% of all proteins present in blood, and by focusing on that category of proteins this work is quite original and provides new insights. Moreover, longitudinal monitoring of plasma proteomes is also not that well established yet, and here we show that this leads to several key findings, even when focusing on supposedly healthy donors.

2. Cohort design and generalizability (age range, sex balance, covariates). The TIMES cohort is restricted to relatively young and middle-aged adults (reported inclusion 18-60 years, actual range 19-56 years, mean age ~34 years), with no representation of older individuals in whom immunosenescence, chronic inflammation, and comorbidities are more prevalent. Yet the manuscript makes broad statements about inter-individual variability in "human plasma proteomes" that may not generalize to older or less healthy populations. A clear justification for excluding older donors is needed, and claims about generalizability should be moderated or validated in an independent cohort. In addition, the sex distribution is imbalanced (32 females vs

19 males), but sex effects on immunoglobulin levels and inflammatory markers are not explored or adjusted for. Please consider (i) providing a more detailed description of recruitment, health status, medications, and lifestyle factors, and (ii) performing at least exploratory analyses testing whether age, sex, and BMI influence key readouts (IgG subclasses, CRP trajectories) or explicitly discussing this as a limitation.

The reviewer is right in that our cohort is still rather small, and contains (as always) biases. However, the strength is not in the number of donors, but in having consistently a dozen of sampling points of each of them over a full year. We acknowledge the bias in the cohort further in the revised "limitations of the study section" addressing the points raised by the reviewer.

<u>This section reads now (line 672-708):</u>

Limitations of the study

Despite the robust design and comprehensive data generated in this study, several limitations need to be acknowledged. First, the cohort size, although sufficient for detecting intra- and inter-individual variations, may limit the generalizability of the findings to broader populations with different demographic or health profiles. Further longitudinal studies with larger, more diverse cohorts are needed to validate and extend these findings.

Secondly, although the TIMES cohort was designed to recruit healthy donors, there is no independent clinical evidence for the absence of disease in all participants. For example, donor 26 is an outlier in terms of high BMI and body fat proportion (Figure EV1), very high basal levels of C-reactive protein (Figure 6) over the whole year, and very low levels of nearly all immunoglobulin subclasses, namely IgG1, IgG2, IgG3, IgA1, IgM (see Figure 5 and Appendix Figure S7, S9, S12, S14, S15). Clinically this is suggestive of common variable immunodeficiency (CVID), the most prevalent human primary immunodeficiency. The disease is usually diagnosed at around 30 years of age because of frequent and severe infections (Bonilla *et al*, 2015). However, donor 26 did not report increased susceptibility for infections. We conclude that her abnormally low immunoglobulin plasma levels might reflect immunodeficiency requiring further diagnosis. This chance finding highlights the potential value of diagnostic plasma proteomics.

Another limitation of the cohort may be that it does not include children/young adults and old people but inter- and intra-donor plasma protein variability may be affected by age. It is well known that immunoglobulin concentrations are gradually built-up during childhood and adolescence (Rich *et al*, 2018). In the elderly, on the other hand, the prevalence of subclinical and clinical health disorders increases. Thus, our conclusions mostly relate to the age group studied, namely between 19 and 56 years.

Moreover, we here focus solely on the ~200 plasma proteins that are consistently detected and can be robustly quantified over all donors and all time points. It has been shown that with more efforts the detectable plasma proteome can be increased to over 1000 proteins, where the gain comes mostly from lower abundant proteins. However, we did not aim for that as it would substantially increase the number of missing values, and thus also CVs and robustness in quantification. However, our study focuses largely on immunoglobulins, which are highly abundant plasma proteins. In the published large-scale in-depth plasma proteomics studies the immunoglobulins are often depleted from the plasma samples in order to reach a deeper depth

in the proteome, whereas when not depleted they are often ignored in the subsequent data analysis, because of their extreme sequence variability.

We now also include an expanded view figure (Figure EV1) in the revised version of the manuscript to provide an expanded phenotypic description of the donors.

To assess the individual and combined contributions of key biological confounders like age, sex and BMI on the inter-donor variability four separate linear regression models were fitted per protein group and per study site (Plasma protein concentrations (nM) were $\log_{10}$-transformed prior to linear regression modeling. Sex was treated as a categorical variable):

1. age alone
2. sex alone
3. BMI alone
4. age, sex, and BMI combined

Confounder-adjusted concentrations were derived by extracting model residuals and adding back the per-group mean log10 concentration, thereby preserving the original intensity scale. Values were subsequently back-transformed to nM.
To quantify biological variability across donors, the inter-donor coefficient of variation (CV) was calculated for each protein group and lab site as the ratio of the standard deviation to the mean of per-donor average concentrations over sampling points. CVs were computed separately for unadjusted data and for each of the four confounder-adjusted datasets.
Differences in inter-donor CV distributions across adjustment models were assessed using non-parametric tests. Pairwise comparisons between all models were performed with the Dunn test and Benjamini–Hochberg correction for multiple testing.
Confounder adjustment for age, sex, BMI, or their combination did not result in significant changes in inter-donor coefficient of variation compared to unadjusted data (Figure EV2-3), suggesting that these biological variables do not or only marginally contribute to inter-donor protein concentration variability in this cohort. We updated the manuscript and supplemental material accordingly (line 308-325, 481-489).

3. Underuse of the dual-laboratory design and potential cross-site biases. A major strength is that ~600 samples were processed independently at two sites using distinct workflows and databases. Yet, after demonstrating strong correlations for a relatively small set of overlapping proteins (~163 after stringent filtering), the authors proceed to perform almost all subsequent biological interpretation on the Utrecht dataset only. This substantially weakens the impact of the "dual-lab" aspect and raises the question of whether the main conclusions are truly robust across workflows. It would be important either to (i) replicate key analyses with the Greifswald data (or a meta-analyzed dataset) and show concordance, or (ii) reposition the paper more explicitly as a primarily single-site biological analysis with a technical cross-site validation. Relatedly, the description of cross-site harmonization could be strengthened: Were sample

aliquots randomized and processed in a blinded fashion at each lab? How was ongoing communication between teams managed to avoid unintentional tuning of methods to match each other? A more systematic description of cross-site QC (including any shared reference or spike-in materials) would help to demonstrate that human and protocol-induced biases are minimized.

We thank the reviewer for the comment. Samples were processed randomized according to each site's established standard operating procedures without prior harmonization of workflows. No shared reference materials or heavy labeled spike-in standards were used at either site, and we acknowledge the absence of a shared external reference material as a limitation. The strong correlation observed for the overlapping proteins after independent processing at both sites therefore represents a conservative and unbiased estimate of cross-site reproducibility.

Regarding the independence of the two sites, we explicitly clarify that each laboratory used its own independent batch standard, samples were measured entirely independently, and no exchange of analytical results, intermediate data, or methodological adjustments took place between the two teams during the sample measurement period. The methods section has been updated accordingly. This ensures that the observed cross-site concordance reflects genuine technical reproducibility rather than methodological tuning toward agreement.
We acknowledge the point regarding the underutilization of the dual-laboratory design in the biological interpretation sections. Therefore, we now include the single protein plot figures with data of both sites in the appendix for a better comparison and use of data of both the Utrecht and Greifswald site. The decision to focus more detailed analyses on the Utrecht dataset was primarily driven by the more advanced mass spectrometry device (timsTOF HT) used in Utrecht. The timsTOF HT incorporates a fourth-generation TIMS-XR analyzer with substantially enhanced ion storage capacity (5x increase in ion charge capacity) and an upgraded digitizer that provides improved vertical dynamic range (4x improvement) compared to the Pro2 TIMS-cartridge. These hardware differences result in different quantitative behaviors, particularly at higher peptide loading masses where the Pro2 exhibits TIMS cartridge saturation while the HT maintains linear quantitative response. The study of Vitko *et al.* (Vitko *et al*, 2024) demonstrated that the two instruments produce different MS2 peak area distributions for the same samples, with the HT showing superior quantitative linearity across peptide loading ranges (median R^2 values of 0.86-0.93 for HT versus 0.53-0.79 for Pro2 in the same loading mass ranges). Furthermore, in the study of Vitko *et al.* (Vitko *et al*, 2024) , spike-in quality control peptides showed significantly different detection and quantification patterns between instruments, with the Pro2 exhibiting decreased peptide identification and median MS2 quantities at higher loading masses while the HT maintained consistent performance and much lower coefficient of variation overall resulting in a more superior dataset. This effect is evident in the inter-donor variability when comparing highly abundant plasma proteins such as albumin or transferrin (Appendix Figure S4-S5).

4. Pre-analytical variation, storage effects, and the circannual objective. The study is framed as an investigation of "circannual fluctuations" in the plasma proteome, yet pre-analytical factors (sample storage time and conditions, freeze-thaw cycles, shipment, acquisition order,

instrument drift) are not thoroughly modeled or quantified. Although the authors describe standardized handling from phlebotomy to freezing, samples from different months will necessarily have different storage durations before MS analysis, and the two sites likely measured samples in different temporal sequences. It is not clear how the authors ensured that the observed stability of immunoglobulins and the variation in CRP and other markers are not confounded by storage or batch effects that are only partially addressed. More quantitative QC (e.g. performance of housekeeping proteins and/or spike-in standards over time, explicit modeling of storage duration and acquisition batch as covariates, and sensitivity analyses) would substantially strengthen the longitudinal claims. Clarifying whether each time point used a unique aliquot (single freeze-thaw) and whether a universal QC sample was run throughout the project would also be important.

Thank you for highlighting the significance of pre-analytical factors. After storage from 2017/2018, the samples were thawed once and then aliquoted for both lab sites and frozen at -70°C again. One aliquot per sample was shipped to the Utrecht lab site. The samples were processed and measured in Utrecht from September 2023 to November 2023, and in Greifswald from November 2023 to December 2023.
We added to the methods section (line143-146): "Samples were stored at -70°C. After thawing and aliquoting, they were frozen and shipped on dry ice to the Utrecht laboratory site. The samples were prepared and measured in Utrecht and Greifswald during the same time period."

Samples from the Greifswald site were processed using nested randomization. We first randomized the order of time points for each individual donor and then randomized the sequence of the resulting donor blocks. Consequently, donors were measured in a random order, with their respective time points appearing as a randomized cluster (response to reviewer comments Figure 1). The methods section was updated accordingly (line 152-157, 203-207).

Measurement quality was controlled using batch standards (plasma pool samples).
As stated in the methods section, a batch effect related to measurement time was identified and corrected using the ComBat approach, resulting in batch-adjusted peptide intensities that were subsequently used for the data analysis at the respective site.

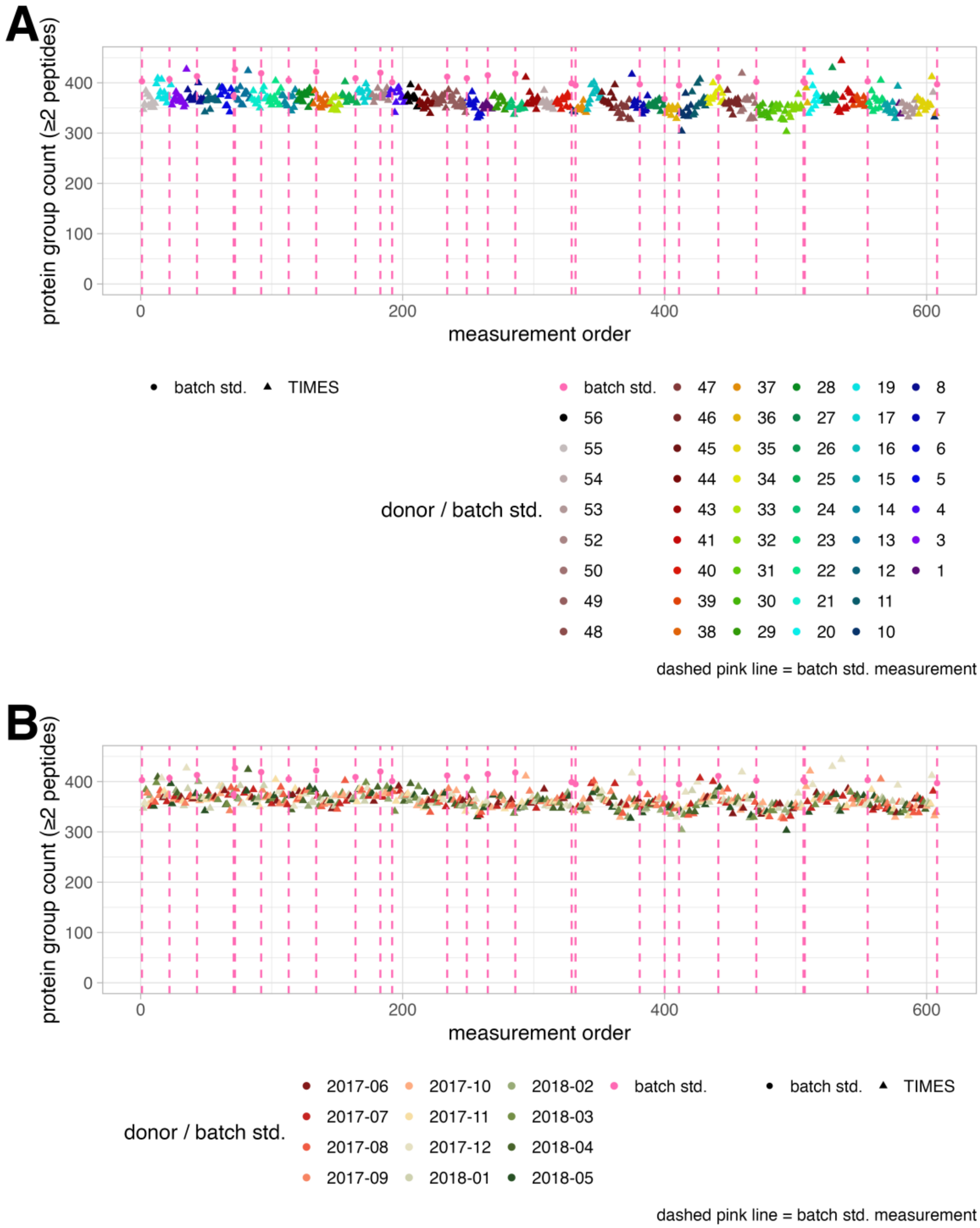

Figure 1 Sample measurement order plot for the Greifswald site. The x-axis represents the measurement order, and the y-axis represents the ID rate (≥2 peptides per protein group). The plot is colored by donor (A) and sampling time (B).

Minor concerns

1. Alignment between Introduction and actual analyses (PTMs and glycosylation). The Introduction gives significant space to discussing post-translational modifications, especially

glycosylation of immunoglobulins, as a contributor to plasma proteome complexity. However, the current analyses are limited to protein-level (or subclass-level) concentrations and do not address PTMs directly. Either include at least a limited analysis of relevant PTMs (e.g. IgG Fc glycosylation features that are accessible from the DIA data) or substantially tone down the PTM-focused narrative and clarify that PTMs are out of scope for this study.

We agree with the reviewer and removed the “single” statement made about PTMs and glycosylation in the introduction as it is not relevant to the current study.

2. Contextualization with UK Biobank and other large proteomics resources. To better position the TIMES cohort, it would be helpful to compare key distributions (e.g. total IgG and subclass concentrations, CRP baseline levels, frequency of high-CRP outliers) with those observed in large-scale resources such as the UK Biobank proteomics datasets or other population-based plasma atlases. Even a qualitative comparison or a brief meta-analysis of published summary statistics would help to assess whether the strong inter-individual differences seen here are typical and to what extent this relatively small cohort reflects broader populations.

We appreciate this comment by the reviewer as it allows us to make an interesting point also addressing his/her major point 1. The very large-scale studies the reviewer refers to in Nature and Science do not include quantitative data on immunoglobulins. Olink states that their platform does not provide comprehensive subclass- resolved immunoglobulin profiling (e.g. IgG1–4, IgA1/2) and acknowledges that for detailed immunoglobulin biology one would need dedicated LC-MS or ELISA assays.

3. Cohort description and covariate analyses. The manuscript would benefit from a more detailed description of the cohort and sampling framework: e.g. recruitment source (blood donors vs general population), frequency of blood donation, smoking status, metabolic status, and any exclusion of chronic diseases or medications that may influence immunoglobulin or CRP levels. Since the BMI range is wide (18.4-39.8 kg/m²), and several donors appear to have chronically elevated CRP, it would be informative to report whether BMI or other available covariates correlate with immunoglobulin profiles or inflammatory markers, even at a descriptive level.

We thank the reviewer for this constructive comment. In response, we have added a detailed phenotypic data table to the supplemental materials part of the manuscript summarizing key donor characteristics including age, sex, BMI, and further available metadata for all donors in the cohort. To systematically assess the influence of biological covariates on plasma protein concentrations, we performed linear regression modeling to adjust protein concentrations for age, sex, and BMI, both individually and in combination (line 308-325, 481-489). Confounder-adjusted concentrations were derived by regressing log10-transformed protein concentrations against the respective covariates and adding back the per-protein mean to the residuals, thereby preserving the original concentration scale. Comparison of inter-donor coefficients of variation (CV) between unadjusted and confounder-adjusted data revealed no significant changes in inter-donor variability for any of the adjustment models, suggesting that age, sex,

and BMI do not substantially drive the observed inter-donor differences in protein concentration in this cohort (Figure EV2-3). We acknowledge that additional covariates such as smoking status, donation frequency, medication use, and chronic disease history were not systematically collected and therefore could not be included in the regression framework. Regarding chronically elevated CRP levels, we note that donors with persistently elevated CRP were not excluded, and the potential influence of subclinical inflammation on immunoglobulin and acute-phase protein profiles is acknowledged as a limitation of the current dataset.

4. Clarification of immunoglobulin peptide handling across workflows. For the cross-site comparison, proteins "containing variable immunoglobulin regions" were removed, while the main biological analyses explicitly rely on immunoglobulin subclass quantification. It would be useful to explain more clearly which immunoglobulin peptides (constant vs variable regions) were used in each pipeline, how subclass-specific peptides were defined, and whether immunoglobulin measurements are directly comparable between sites. This is particularly important given the different FASTA/databases used (compact blood-specific vs large UniProt with isoforms and manually added Ig sequences).

We apologize for the confusion. The Ig variable domains (e.g. IGHV, IGHJ, IGKV gene identifiers) were removed from both sites, because they are highly heterogeneous and unique for each individual, making their quantification ambiguous. For subclass-specific quantification we used solely unique peptides of each heavy-chain constant region. Both FASTA files contained the same immunoglobulin sequences; the Utrecht (blood-specific) database additionally included the 5% most prevalent single–amino acid variants as presented in Uniprot. Despite this difference, Ig subclass quantification was highly comparable between the two sites, as shown in response to Comment 3 and the revised Appendix Figure S7-S12, S14-S15.

5. Quality control and sample identity checks. The detection of an apparently mislabelled/duplicated sample (December sample of donor 27 clustering with donor 4) illustrates the value of longitudinal design for QC. However, the criteria used to flag this sample as mislabelled (UMAP and clustering thresholds) should be described more systematically, and the authors should clarify whether other potential swaps or mix-ups were formally ruled out. In addition, please expand the description of QC procedures: were any stable isotope-labeled or protein spike-in controls used, and were pooled reference samples monitored across the entire acquisition to control for long-term instrument drift and plate-to-plate variation? Including summary QC plots (e.g. CVs of reference proteins/spike-ins over time) would be helpful.

We thank the reviewer for this comment. To systematically identify potential sample mislabelling or mix-ups, we used a quantitative Euclidean distance approach on the UMAP projection. For each donor, the median UMAP coordinates across all longitudinal samples were calculated as the donor-specific reference centroid. The Euclidean distance of each individual sample from its corresponding donor centroid was then computed, and samples exceeding a defined distance threshold (mean + 5 × SD across all samples) were flagged as potential outliers (now present in methods section, line 274-280). This approach revealed one sample — the December 2017

timepoint of donor 27 — clustering aberrantly with donor 4, consistent with a mislabelling or sample swap event.

Regarding additional QC measures, we would like to clarify that pooled reference samples (pool batch QC) were included and measured repeatedly throughout the entire data acquisition to monitor long-term instrument drift and plate-to-plate variation (response to reviewer comments Figure 2). No stable isotope-labeled spike-in standards were used in this study.

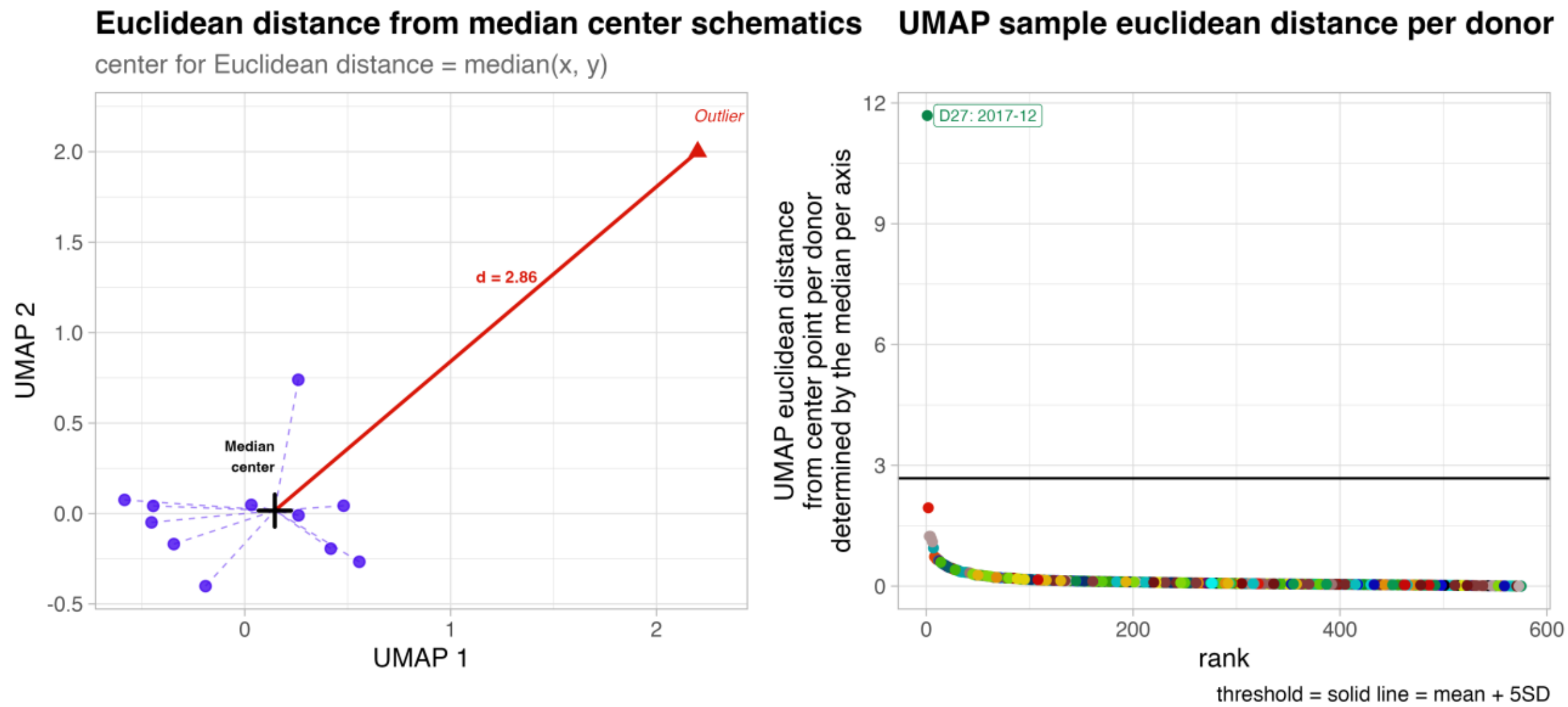

Figure 2 UMAP sample euclidean distance per donor.

References

Álvez MB, Bergström S, Kenrick J, Johansson E, Åberg M, Akyildiz M, Altay O, Sköld H, Antonopoulos K, Apostolakis E, *et al* (2025) A human pan-disease blood atlas of the circulating proteome. *Science*: eadx2678

Bonilla FA, Khan DA, Ballas ZK, Chinen J, Frank MM, Hsu JT, Keller M, Kobrynski LJ, Komarow HD, Mazer B, *et al* (2015) Practice parameter for the diagnosis and management of primary immunodeficiency. *J Allergy Clin Immunol* 136: 1186-1205.e78

Distler U, Yoo HB, Kardell O, Hein D, Sielaff M, Scherer M, Jozefowicz AM, Leps C, Gomez-Zepeda D, Toerne C von, *et al* (2025) Multicenter evaluation of label-free quantification in human plasma on a high dynamic range benchmark set. *Nat Commun* 16: 8774

Eldjarn GH, Ferkingstad E, Lund SH, Helgason H, Magnusson OTh, Gunnarsdottir K, Olafsdottir TA, Halldorsson BV, Olason PI, Zink F, *et al* (2023) Large-scale plasma proteomics comparisons through genetics and disease associations. *Nature*: 1–11

Pearson TA, Mensah GA, Alexander RW, Anderson JL, Cannon RO, Criqui M, Fadl YY, Fortmann SP, Hong Y, Myers GL, *et al* (2003) Markers of inflammation and cardiovascular disease: application to clinical and public health practice: A statement for healthcare professionals from the Centers for Disease Control and Prevention and the American Heart Association. *Circulation* 107: 499–511

Rich RR, Fleisher TA, Shearer WT & Schroeder H (2018) Clinical Immunology: Principles and Practice 5th Edition. Edition 5th (ed) Elsevier

Richard VR, Mitsa G, Eshghi A, Chaplygina D, Mohammed Y, Goodlett DR, Zahedi RP, Thevis M & Borchers CH (2024) Establishing Personalized Blood Protein Reference Ranges Using Noninvasive Microsampling and Targeted Proteomics: Implications for Antidoping Strategies. *J Proteome Res* 23: 1779–1787

Ridker PM, Hennekens CH, Buring JE & Rifai N (2000) C-Reactive Protein and Other Markers of Inflammation in the Prediction of Cardiovascular Disease in Women. *N Engl J Med* 342: 836–843

Rijswijck DMH van, Bondt A, Raafat D, Holtfreter S, Wietschel KA, Lans SPA van der, Völker U, Bröker BM & Heck AJR (2025) Persistent IgG1 clones dominate and personalize the plasma antibody repertoire. *Sci Adv* 11: eadt7746

Varnavides G, Madern M, Anrather D, Hartl N, Reiter W & Hartl M (2022) In Search of a Universal Method: A Comparative Survey of Bottom-Up Proteomics Sample Preparation Methods. *J Proteome Res*

Vitko D, Chou W-F, Golmaei SN, Lee J-Y, Belthangady C, Blume J, Chan JK, Flores-Campuzano G, Hu Y, Liu M, *et al* (2024) timsTOF HT Improves Protein Identification and Quantitative Reproducibility for Deep Unbiased Plasma Protein Biomarker Discovery. *J Proteome Res* 23: 929–938

31st Mar 2026

Manuscript Number: MSB-2025-13450R
Title: Immunoglobulin sub-class levels define inter-donor plasma variability: a longitudinal dual-lab study
Author: Stephan Michalik
Sofia Kalaidopoulou Nteak
Nicolas Drouin
Manuela Gesell-Salazar
Elke Hammer
Vishnu Dhople
Silva Holtfreter
Stefan Weiss
Henk W P van den Toorn
Barbara Bröker
Grazyna Domanska
Uwe Völker
Albert Heck

Dear Albert,

Thank you for submitting the revised version of your manuscript. We have now received feedback from two reviewers who evaluated the revision.

As you will see below, Reviewer #2 is satisfied with the changes made and supports publication. Reviewer #3, who was invited to evaluate the technical aspects, acknowledges that the revised version has improved but maintains their previous position regarding the perceived level of conceptual advance. As this concern was already carefully considered at the previous decision stage, and taking into account the overall balance of feedback from all three reviewers, we are inviting a minor revision.

Given that Reviewer #3 explicitly acknowledges the resource value of the study (which we also recognize), we ask that you further highlight the provided datasets and its potential utility for future studies.

On a more editorial level, please do the following:

1. Data availability:

- The computer code should be deposited in a public repository (e.g., GitHub).
- For human proteomics data, in accordance with the journal's data policy, we ask authors-where feasible and consistent with participant consent-to deposit fully anonymized clinical datasets in public databases at the time of publication (see examples here: https://link.springer.com/article/10.15252/emmm.202317459#data-availability). Alternatively, several databases, such as EGA and dbGaP, also provide controlled-access repositories that can be used for human proteomics data and may be considered for data deposition.
- Please provide resolvable links to the pages where the data and computer code can be accessed.

2. Please address the following discrepancies in author names between the manuscript file and the online submission system: Vishnu Dhople (submission system) vs. Vishnu Mukund Dhople (manuscript),Henk W. P. van den Toorn (submission system) vs. Henk van den Toorn (manuscript). Additionally, please ensure that the email addresses for both corresponding authors are included on the manuscript cover page.

3. Remove "synopsis image" and "synopsis text" from the manuscript file and provide them as separate files.

4. "Ethics declarations" should be renamed to "Disclosure and Competing Interests Statement".

5. Remove "Authors' contribution" section from the manuscript file.

6. Both corresponding authors' names should be listed in the author checklist.

7. The manuscript sections should be in the following order: Title page - Abstract - Introduction - Results - Discussion - Methods -Data Availability - Acknowledgments - Disclosure and Competing Interests Statement - References - Figure Legends - Expanded View Figure Legends.

8. 'Materials & Methods' should be renamed to 'Methods'.

9. Please download and fill our Reagents and Tools Table template (.docx), which you can find in our author guidelines: https://link.springer.com/journal/44320/submission-guidelines#structuredmethods.

When submitting your revised manuscript, please do not include the Reagents and Tools Table in the Methods section of the manuscript but upload it as a separate file choosing the file type "Reagent Table".

10. Appendix
- Remove the "Appendix information" section from the main manuscript file.
- In the Appendix file, the first title page should include only: "Appendix for [manuscript title]" and a Table of Contents.
- The methods and cohort description should be combined into a single "Supplementary Methods" section and placed after all Appendix figures and tables.
- "used R packages table" should be removed from Appendix and the related information should be included in the "Reagents and Tools" table.

11. BioRender should be acknowledged at the end of the Methods section in the following way:
Graphics:
(some of the... OR Figure #... OR synopsis) Graphics were created with BioRender.com

12. Please address the following issues in figure legends :
- Please note that the figure 5 is mislabeled as figure 3 in the manuscript. This needs to be rectified。
- Please note that the figure title for EV figures 1-4 are missing in the manuscript. This needs to be rectified.
- Please indicate the statistical test used for data analysis in the legends of figures EV-2.
- Please note that the box plots need to be defined in terms of minima, maxima, centre, bounds of box and whiskers, and percentile in the legends of figures EV-2.
- Please note that information related to n is missing in the legends of figures 4; 6; EV-2.
- Please note that the error bars are not defined in the legends of figures 6.

Use the following link to submit your revised paper:

https://msb.msubmit.net/cgi-bin/main.plex

As a matter of course, please make sure that you have correctly followed the instructions for authors as given on the submission website.

Thank you for submitting this paper to Molecular Systems Biology.

Kind regards,
Jingyi

Jingyi Hou, PhD
Senior Editor
Molecular Systems Biology

*** PLEASE NOTE *** As part of the EMBO Press transparent editorial process initiative (see our Editorial at https://dx.doi.org/10.1038/msb.2010.72 , Molecular Systems Biology will publish online a Review Process File to accompany accepted manuscripts. When preparing your letter of response, please be aware that in the event of acceptance, your cover letter/point-by-point document will be included as part of this File, which will be available to the scientific community. More information about this initiative is available in our Instructions to Authors. If you have any questions about this initiative, please contact the editorial office (msb@embo.org).

Reviewer #2:

The revised manuscript demonstrates substantial improvement across all sections. The authors have carefully addressed all the reviewer's comments, and the present version is considered ready for publication.

Reviewer #3:

The authors have clearly made a serious effort to address the previous comments, and the revised version is improved in

several respects, including clarification of immunoglobulin peptide handling, cohort limitations, and aspects of quality control and sample handling. However, my overall assessment remains unchanged. Although the revision improves the framing and makes the manuscript more balanced, the study still reads as largely descriptive, and the main conclusions remain primarily observational rather than providing the level of conceptual or systems level advance I would expect for Molecular Systems Biology. I also remain concerned that the dual site design, while valuable, is not fully leveraged in support of the main biological conclusions, since much of the downstream interpretation still relies primarily on one site. In addition, several important limitations are now acknowledged but not fully resolved, including cohort composition, incomplete phenotypic characterization, the absence of shared standards for rigorous cross site harmonization, and the possible influence of subclinical inflammatory or immune abnormalities in some donors. Overall, I think the dataset may be useful and the work may be of interest to a proteomics focused readership, but I do not think the revised manuscript reaches the threshold of conceptual advance expected for this journal. For that reason, I am unable to support publication in Molecular Systems Biology, although I believe the study may be better suited to a more technically oriented proteomics or resource focused journal.

Addressing Editor's comments:

Data Availability (Point 1): The computer code has been deposited in a public Zeneodo repository (https://doi.org/10.5281/zenodo.19695248), and a resolvable link is now provided in the Data Availability section. The human proteomics data have been deposited at the transfer unit for data and biomaterials at the University Medicine Greifswald (https://transfer.ship-med.uni-greifswald.de/FAIRequest/?lang=en). Resolvable links to both the code and data repositories are included in the revised manuscript.

Author Names and Contact Details (Point 2): Author names have been harmonized between the manuscript file and the submission system. The email addresses of both corresponding authors are now included on the manuscript cover page.

Synopsis Image and Text (Point 3): The synopsis image and synopsis text have been removed from the manuscript file and are now provided as separate files.

Ethics Declarations (Point 4): The section has been renamed to "Disclosure and Competing Interests Statement".

Authors' Contribution Section (Point 5): The "Authors' Contribution" section has been removed from the manuscript file.

Author Checklist (Point 6): Both corresponding authors are now listed in the author checklist.

Manuscript Section Order (Point 7): The manuscript sections have been reordered in accordance with the journal's requirements.

Methods Section Title (Point 8): "Materials & Methods" has been renamed to "Methods".

Reagents and Tools Table (Point 9): The Reagents and Tools Table has been completed using the journal's template and is uploaded as a separate file of type "Reagent Table".

Appendix (Point 10): The "Appendix information" section has been removed from the main manuscript. The Appendix file now begins with a title page ("Appendix for Immunoglobulin sub-class levels define inter-donor plasma variability: a longitudinal dual-lab study") and a Table of Contents. Methods and cohort description have been merged into a single "Supplementary Methods" section, placed after all Appendix figures and tables. The "used R packages table" has been removed from the Appendix and incorporated into the Reagents and Tools Table.

BioRender Acknowledgment (Point 11): BioRender has been acknowledged at the end of the Methods section as requested.

Figure Legends (Point 12): Figure 5, previously mislabeled as Figure 3 in the figure captions, has been corrected. Figure titles for EV Figures 1–4 have been added in font face bold. The statistical test used in EV Figure 2 is now specified in the figure caption. Box plots in EV Figure 2 are now fully defined in the caption in terms of minima, maxima, centre, bounds of box and whiskers, and percentiles. Sample size information (n) has been added to the legends of Figures 4 (new revised figure), 6 (new revised figure), and EV-2 (in the figure caption). Error bars in Figure 6 are now defined in the figure caption.

8th May 2026
Manuscript number: MSB-2025-13450RR
Title: Immunoglobulin sub-class levels define inter-donor plasma variability: a longitudinal dual-lab study

Dear Albert,

Thank you again for sending us your revised manuscript. We are now satisfied with the modifications made and I am pleased to inform you that your paper has been accepted for publication.

Your manuscript will be processed for publication by EMBO Press. It will be copy edited and you will receive page proofs prior to publication. Please note that you will be contacted by Springer Nature Author Services to complete licensing and payment information.

You may qualify for financial assistance for your publication charges - either via a Springer Nature fully open access agreement or an EMBO initiative. Check your eligibility: https://link.springer.com/journal/44320/how-to-publish-with-us

Should you be planning a Press Release on your article, please get in contact with embo_production@springernature.com as early as possible in order to coordinate publication and release dates.

If you have any questions, please do not hesitate to contact the Editorial Office. Thank you for your contribution to Molecular Systems Biology.

Kind regards,
Jingyi

Jingyi Hou, PhD
Senior Editor
Molecular Systems Biology

>>> Please note that it is Molecular Systems Biology policy for the transcript of the editorial process (containing referee reports and your response letter) to be published as an online supplement to each paper. If you do NOT want this, you will need to inform the Editorial Office via email immediately. More information is available here: https://link.springer.com/partners/embo-press/editorial-policies#Peer%20review

EMBO Press Author Checklist

Corresponding Author Name: Albert J.R. Heck and Uwe Völker	
Journal Submitted to: Molecular Systems Biology	
Manuscript Number: MSB-2025-13450	

USEFUL LINKS FOR COMPLETING THIS FORM
The EMBO Journal - Author Guidelines
EMBO Reports - Author Guidelines
Molecular Systems Biology - Author Guidelines
EMBO Molecular Medicine - Author Guidelines

Reporting Checklist for Life Science Articles (updated January
This checklist is adapted from Materials Design Analysis Reporting (MDAR) Checklist for Authors. MDAR establishes a minimum set of requirements in transparent reporting in the life sciences (see Statement of Task: 10.31222/osf.io/9sm4x). Please follow the journal's guidelines in preparing your
Please note that a copy of this checklist will be published alongside your article.

Abridged guidelines for figures
1. Data
The data shown in figures should satisfy the following conditions:
- ➞ the data were obtained and processed according to the field's best practice and are presented to reflect the results of the experiments in an accurate and unbiased manner.
- ➞ ideally, figure panels should include only measurements that are directly comparable to each other and obtained with the same assay.
- ➞ plots include clearly labeled error bars for independent experiments and sample sizes. Unless justified, error bars should not be shown for technical
- ➞ if n<5, the individual data points from each experiment should be plotted. Any statistical test employed should be justified.
- ➞ Source Data should be included to report the data underlying figures according to the guidelines set out in the authorship guidelines on Data

2. Captions
Each figure caption should contain the following information, for each panel where they are relevant:
- ➞ a specification of the experimental system investigated (eg cell line, species name).
- ➞ the assay(s) and method(s) used to carry out the reported observations and measurements.
- ➞ an explicit mention of the biological and chemical entity(ies) that are being measured.
- ➞ an explicit mention of the biological and chemical entity(ies) that are altered/varied/perturbed in a controlled manner.
- ➞ the exact sample size (n) for each experimental group/condition, given as a number, not a range;
- ➞ a description of the sample collection allowing the reader to understand whether the samples represent technical or biological replicates (including how many animals, litters, cultures, etc.).
- ➞ a statement of how many times the experiment shown was independently replicated in the laboratory.
- ➞ definitions of statistical methods and measures:
  - common tests, such as t-test (please specify whether paired vs. unpaired), simple χ2 tests, Wilcoxon and Mann-Whitney tests, can be unambiguously identified by name only, but more complex techniques should be described in the methods section;
  - are tests one-sided or two-sided?
  - are there adjustments for multiple comparisons?
  - exact statistical test results, e.g., P values = x but not P values < x;
  - definition of 'center values' as median or average;
  - definition of error bars as s.d. or s.e.m.

Please complete ALL of the questions below.
Select "Not Applicable" only when the requested information is not relevant for your study.

Materials

Newly Created Materials	Information included in the manuscript?	In which section is the information available? (Reagents and Tools Table, Materials and Methods, Figures, Data Availability Section)
New materials and reagents need to be available; do any restrictions apply?	Yes	Reagents and Tool & Materials and Methods

Antibodies	Information included in the manuscript?	In which section is the information available? (Reagents and Tools Table, Materials and Methods, Figures, Data Availability Section)
For **antibodies** provide the following information:<br>- Commercial antibodies: RRID (if possible) or supplier name, catalogue number and or/clone number<br>- Non-commercial: RRID or citation	Not Applicable	

DNA and RNA sequences	Information included in the manuscript?	In which section is the information available? (Reagents and Tools Table, Materials and Methods, Figures, Data Availability Section)
**Short novel DNA or RNA including primers, probes**: provide the sequences.	Not Applicable	

Cell materials	Information included in the manuscript?	In which section is the information available? (Reagents and Tools Table, Materials and Methods, Figures, Data Availability Section)
**Cell lines:** Provide species information, strain. Provide accession number in repository **OR** supplier name, catalog number, clone number, and/**OR** RRID.	Not Applicable	
**Primary cultures:** Provide species, strain, sex of origin, genetic modification status.	Not Applicable	
Report if the cell lines were recently **authenticated** (e.g., by STR profiling) and tested for mycoplasma contamination.	Not Applicable	

Experimental animals	Information included in the manuscript?	In which section is the information available? (Reagents and Tools Table, Materials and Methods, Figures, Data Availability Section)
**Laboratory animals or Model organisms:** Provide species, strain, sex, age, genetic modification status. Provide accession number in repository **OR** supplier name, catalog number, clone number, **OR** RRID.	Not Applicable	
**Animal observed in or captured from the field:** Provide species, sex, and age where possible.	Not Applicable	
Please detail **housing and husbandry conditions**.	Not Applicable	

Plants and microbes	Information included in the manuscript?	In which section is the information available? (Reagents and Tools Table, Materials and Methods, Figures, Data Availability Section)
**Plants:** provide species and strain, ecotype and cultivar where relevant, unique accession number if available, and source (including location for collected wild specimens).	Not Applicable	
**Microbes:** provide species and strain, unique accession number if available, and source.	Not Applicable	

Human research participants	Information included in the manuscript?	In which section is the information available? (Reagents and Tools Table, Materials and Methods, Figures, Data Availability Section)
If collected and within the bounds of privacy constraints report on age, sex and gender or ethnicity for all study participants.	Yes	Data Availability Section

Core facilities	Information included in the manuscript?	In which section is the information available? (Reagents and Tools Table, Materials and Methods, Figures, Data Availability Section)
If your work benefited from core facilities, was their service mentioned in the acknowledgments section?	Not Applicable	

Design

Study protocol	Information included in the manuscript?	In which section is the information available? (Reagents and Tools Table, Materials and Methods, Figures, Data Availability Section)
If study protocol has been **pre-registered, provide DOI in the manuscript**. For clinical trials, provide the trial registration number **OR** cite DOI.	Not Applicable	
Report the **clinical trial registration number** (at ClinicalTrials.gov or equivalent), where applicable.	Not Applicable	

Laboratory protocol	Information included in the manuscript?	In which section is the information available? (Reagents and Tools Table, Materials and Methods, Figures, Data Availability Section)
Provide DOI OR other citation details if **external detailed step-by-step protocols** are available.	Yes	Materials and Methods

Experimental study design and statistics	Information included in the manuscript?	In which section is the information available? (Reagents and Tools Table, Materials and Methods, Figures, Data Availability Section)
Include a statement about **sample size** estimate even if no statistical methods were used.	Yes	Materials and Methods
Were any steps taken to minimize the effects of subjective bias when allocating animals/samples to treatment (e.g. **randomization procedure**)? If yes, have they been described?	Yes	Materials and Methods
Include a statement about **blinding** even if no blinding was done.	Yes	Materials and Methods
Describe **inclusion/exclusion criteria** if samples or animals were excluded from the analysis. Were the criteria pre-established?<br><br>If sample or data points were omitted from analysis, report if this was due to attrition or intentional exclusion and provide justification.	Yes	Materials and Methods
For every figure, are **statistical tests** justified as appropriate? Do the data meet the assumptions of the tests (e.g., normal distribution)? Describe any methods used to assess it. Is there an estimate of variation within each group of data? **Is** the variance similar between the groups that are being statistically compared?	Yes	Materials and Methods

Sample definition and in-laboratory replication	Information included in the manuscript?	In which section is the information available? (Reagents and Tools Table, Materials and Methods, Figures, Data Availability Section)
In the figure legends: state number of times the experiment was **replicated** in laboratory.	Not Applicable	
In the figure legends: define whether data describe **technical or biological replicates**.	Not Applicable	

Ethics

Ethics	Information included in the manuscript?	In which section is the information available? (Reagents and Tools Table, Materials and Methods, Figures, Data Availability Section)
Studies involving **human participants**: State details of **authority granting ethics approval** (IRB or equivalent committee(s), provide reference number for approval.	Yes	Materials and Methods
Studies involving **human participants**: Include a statement confirming that **informed consent** was obtained from all subjects and that the experiments conformed to the principles set out in the WMA Declaration of Helsinki and the Department of Health and Human Services Belmont Report.	Yes	Materials and Methods
Studies involving **human participants:** For publication of **patient photos**, include a statement confirming that consent to publish was obtained.	Not Applicable	
Studies involving experimental **animals**: State details of **authority granting ethics approval** (IRB or equivalent committee(s), provide reference number for approval. Include a statement of compliance with ethical regulations.	Not Applicable	
Studies involving **specimen and field samples:** State if relevant **permits** obtained, provide details of authority approving study; if none were required, explain why.	Not Applicable	

Dual Use Research of Concern (DURC)	Information included in the manuscript?	In which section is the information available? (Reagents and Tools Table, Materials and Methods, Figures, Data Availability Section)
Could your study fall under dual use research restrictions? Please check biosecurity documents and list of **select agents and toxins** (CDC): https://www.selectagents.gov/sat/list.htm	Not Applicable	
If you used a select agent, is the security level of the lab appropriate and reported in the manuscript?	Not Applicable	
If a study is subject to dual use research of concern regulations, is the name of the **authority granting approval and reference number** for the regulatory approval provided in the manuscript?	Not Applicable	

Reporting

The MDAR framework recommends adoption of discipline-specific guidelines, established and endorsed through community initiatives. Journals have their own policy about requiring specific guidelines and recommendations to complement MDAR.

Adherence to community standards	Information included in the manuscript?	In which section is the information available? (Reagents and Tools Table, Materials and Methods, Figures, Data Availability Section)
State if relevant guidelines or checklists (e.g., **ICMJE, MIBBI, ARRIVE, PRISMA**) have been followed or provided.	Not Applicable	
For **tumor marker prognostic studies,** we recommend that you follow the **REMARK** reporting guidelines (see link list at top right). See author guidelines, under 'Reporting Guidelines'. Please confirm you have followed these guidelines.	Not Applicable	
For **phase II and III randomized controlled trials**, please refer to the **CONSORT** flow diagram (see link list at top right) and submit the CONSORT checklist (see link list at top right) with your submission. See author guidelines, under 'Reporting Guidelines'. Please confirm you have submitted this list.	Not Applicable	

Data Availability

Data availability	Information included in the manuscript?	In which section is the information available? (Reagents and Tools Table, Materials and Methods, Figures, Data Availability Section)
Have **primary datasets** been deposited according to the journal's guidelines (see 'Data Deposition' section) and the respective accession numbers provided in the Data Availability Section?	Not Applicable	
Were **human clinical and genomic datasets** deposited in a public access-controlled repository in accordance to ethical obligations to the patients and to the applicable consent agreement?	Yes	Data Availability Section
Are **computational models** that are central and integral to a study available without restrictions in a machine-readable form? Were the relevant accession numbers or links provided?	Not Applicable	
If publicly available data were reused, provide the respective **data citations in the reference list**.	Not Applicable	
