# Supplementary material for: Immunoglobulin sub-class levels define inter-donor plasma variability: a longitudinal dual-lab study
Source: Mol Syst Biol. 2026 May 26;22(8):1312–32. doi: 10.1038/s44320-026-00218-5 (PMC13434392; doi:10.1038/s44320-026-00218-5)
Supplement: Supplementary file 3 [file 44320_2026_218_MOESM3_ESM.pdf]

Expanded View Figures

Figure EV1. TIMES cohort - donor phenotypic data. ▶

TIMES cohort phenotypic data

demographics and body composition

demographics			body composition					
donor	sex	age [years]	height [cm]	weight [kg]	BMI	body fat [%]	body water [%]	body muscle [%]
1	F	46	174	67.68 ± 0.46	22.36 ± 0.15	28.57 ± 3.37	50.2 ± 0.57	34.58 ± 1.04
3	M	32	191	96.71 ± 1.36	26.51 ± 0.37	19.46 ± 1.54	54.67 ± 1.82	40.82 ± 2.12
4	M	37	180	98.46 ± 1.58	30.39 ± 0.49	26.54 ± 1.2	50.16 ± 1.1	37.76 ± 1.26
5	F	31	159	53.15 ± 1.03	21.02 ± 0.41	24.82 ± 1.45	52.84 ± 0.7	37.24 ± 0.38
6	M	26	189	78.81 ± 3.35	22.06 ± 0.94	13.63 ± 1.28	59.06 ± 1.22	44.09 ± 1.71
7	F	45	161	62.92 ± 0.72	24.28 ± 0.28	32.48 ± 2.37	47.23 ± 0.53	32.81 ± 0.53
8	M	44	180	100.5 ± 0.98	31.02 ± 0.3	27.7 ± 2.14	49.62 ± 1.54	37.21 ± 1.59
9	F	29	167	58.87 ± 0.38	21.11 ± 0.14	26.37 ± 0.51	54 ± 0.62	37.57 ± 0.31
10	F	32	157	56 ± 0.94	22.72 ± 0.38	28.14 ± 1.8	50.26 ± 0.44	35.59 ± 0.28
11	M	23	198	133.99 ± 3.61	34.18 ± 0.92	28.08 ± 2.08	48.7 ± 1.82	36.92 ± 1.73
12	F	44	175	69.36 ± 0.42	22.65 ± 0.14	30.22 ± 2.48	49.46 ± 0.63	34.04 ± 0.64
13	F	28	174	70.07 ± 0.57	23.14 ± 0.19	28.52 ± 0.81	50.4 ± 1.05	35.6 ± 0.38
14	M	35	188	96.9 ± 0.73	27.42 ± 0.21	21.26 ± 1.94	53.58 ± 2	40.1 ± 2.15
15	F	37	170	63.09 ± 0.6	21.83 ± 0.21	27.52 ± 2.19	51.42 ± 0.34	35.7 ± 0.34
16	M	31	188	125.23 ± 2.45	35.43 ± 0.69	29.96 ± 1.32	48.04 ± 1.05	36.54 ± 1.12
17	F	35	174	68.84 ± 2	22.74 ± 0.66	28.38 ± 2.6	50.53 ± 0.55	35.29 ± 0.58
19	F	52	164	60.1 ± 1.01	22.35 ± 0.37	30.56 ± 3.52	49.02 ± 1	33.29 ± 1.22
20	M	56	180	83.41 ± 0.82	25.74 ± 0.25	23.27 ± 3.43	52.81 ± 2.24	38.6 ± 2.33
21	M	23	198	89.24 ± 0.84	22.76 ± 0.22	13.06 ± 0.91	59.19 ± 1.35	44.21 ± 2.03
22	F	50	164	76.43 ± 1.97	28.42 ± 0.73	41.76 ± 1.21	41.93 ± 1.53	29.21 ± 0.87
23	F	26	178	70.51 ± 1.37	22.25 ± 0.43	26.97 ± 0.39	51.67 ± 1.84	36.39 ± 0.73
24	F	27	168	52.07 ± 0.53	18.45 ± 0.19	19.59 ± 2.81	57.49 ± 0.8	40.2 ± 0.2
25	M	24	179	80.23 ± 3.44	25.04 ± 1.07	18.4 ± 2.1	55.83 ± 1.17	42.33 ± 0.51
26	F	22	164	103.42 ± 1.36	38.45 ± 0.5	48.8 ± 0.98	41.23 ± 0.29	30.73 ± 0.19
27	M	29	172	84.91 ± 1.1	28.7 ± 0.37	26.1 ± 4.4	50.74 ± 1.82	38.39 ± 2.1
28	F	55	176	64.6 ± 0.69	20.85 ± 0.22	27.49 ± 5.45	51.59 ± 1.65	34.64 ± 2.04
29	F	39	170	70.02 ± 0.9	24.23 ± 0.31	32.06 ± 1.4	47.93 ± 0.73	33.5 ± 0.22
30	M	26	174	77.81 ± 1.5	25.7 ± 0.49	20.25 ± 1.26	54.42 ± 0.55	41.34 ± 0.33
31	F	45	178	84.01 ± 0.55	26.52 ± 0.17	37.28 ± 1.16	44.69 ± 1.2	31.08 ± 0.44
32	M	45	188	113.39 ± 1.17	32.08 ± 0.33	28.76 ± 3.12	48.93 ± 2.13	36.52 ± 2.14
33	F	32	160	54 ± 0.77	21.09 ± 0.3	25.45 ± 1.8	52.67 ± 0.44	37.02 ± 0.17
34	F	32	165	92.8 ± NA	34.09 ± NA	45.4 ± NA	42.2 ± NA	30.7 ± NA
35	F	42	160	66.54 ± 1.03	25.99 ± 0.4	35.88 ± 0.59	45.03 ± 0.99	31.6 ± 0.61
36	F	36	168	59.97 ± 0.25	21.25 ± 0.09	25.89 ± 2.6	52.38 ± 0.16	36.42 ± 0.5
37	F	32	158	58.77 ± 1.34	23.54 ± 0.54	30.07 ± 0.85	48.97 ± 1.18	34.72 ± 0.82
38	F	35	185	87.64 ± 1.46	25.61 ± 0.43	34.15 ± 0.94	46.85 ± 1.3	32.81 ± 0.49
39	F	24	173	65.44 ± 1.46	21.87 ± 0.49	25.93 ± 0.49	52.34 ± 2.01	37.07 ± 0.92
40	M	21	183	68.11 ± 0.36	20.34 ± 0.11	11.6 ± 1.53	61.32 ± 1.76	46.03 ± 0.48
41	F	23	176	64.19 ± 0.74	20.72 ± 0.24	23.48 ± 0.86	54.28 ± 1.55	38.3 ± 0.52
43	M	26	189	77.17 ± 1.5	21.6 ± 0.42	12.89 ± 0.44	60 ± 0.48	44.68 ± 0.69
44	F	24	165	71.78 ± 0.87	26.36 ± 0.32	34.49 ± 2.15	46 ± 2.66	33.09 ± 1.9
45	M	35	180	106.25 ± 1.05	32.79 ± 0.32	29.31 ± 1.53	48.36 ± 1.13	36.54 ± 1.2
46	M	52	187	86.69 ± 0.84	24.79 ± 0.24	20.02 ± 3.28	54.95 ± 2.23	40.3 ± 2.46
47	M	39	175	71.17 ± 0.8	23.24 ± 0.26	18.42 ± 0.52	56.17 ± 0.24	41.79 ± 0.67
48	F	33	165	63.42 ± 0.98	23.3 ± 0.36	29.34 ± 1.36	49.67 ± 0.87	35.01 ± 0.45
49	F	28	175	67.06 ± 2.2	21.9 ± 0.72	26.57 ± 0.35	52.06 ± 2.02	36.56 ± 0.82
50	F	28	151	56.26 ± 0.93	24.67 ± 0.41	31.4 ± 0.65	47.72 ± 0.6	34.4 ± 0.8
52	M	35	183	77.48 ± 0.54	23.14 ± 0.16	16.93 ± 0.66	57.02 ± 0.49	42.39 ± 1.11
53	F	55	160	58.36 ± 3.84	22.8 ± 1.5	30.83 ± 6.63	48.48 ± 3.51	32.93 ± 2.53
54	F	28	158	72.77 ± 1.46	29.15 ± 0.58	40.53 ± 3.66	41.88 ± 3.72	30.27 ± 2.89
55	F	19	165	60.16 ± 0.77	22.1 ± 0.28	25.55 ± 0.7	52.24 ± 1.66	37.52 ± 1.04
56	F	38	171	67.98 ± 0.81	23.25 ± 0.28	29.93 ± 1.58	49.14 ± 0.76	34.37 ± 0.32

sex coding: F female | M male
color scale ranges: deviation proportion = (value - mean(values)) / max(value - mean(values)) from -1 to 0 | 0 | from 0 to 1

© The Author(s)

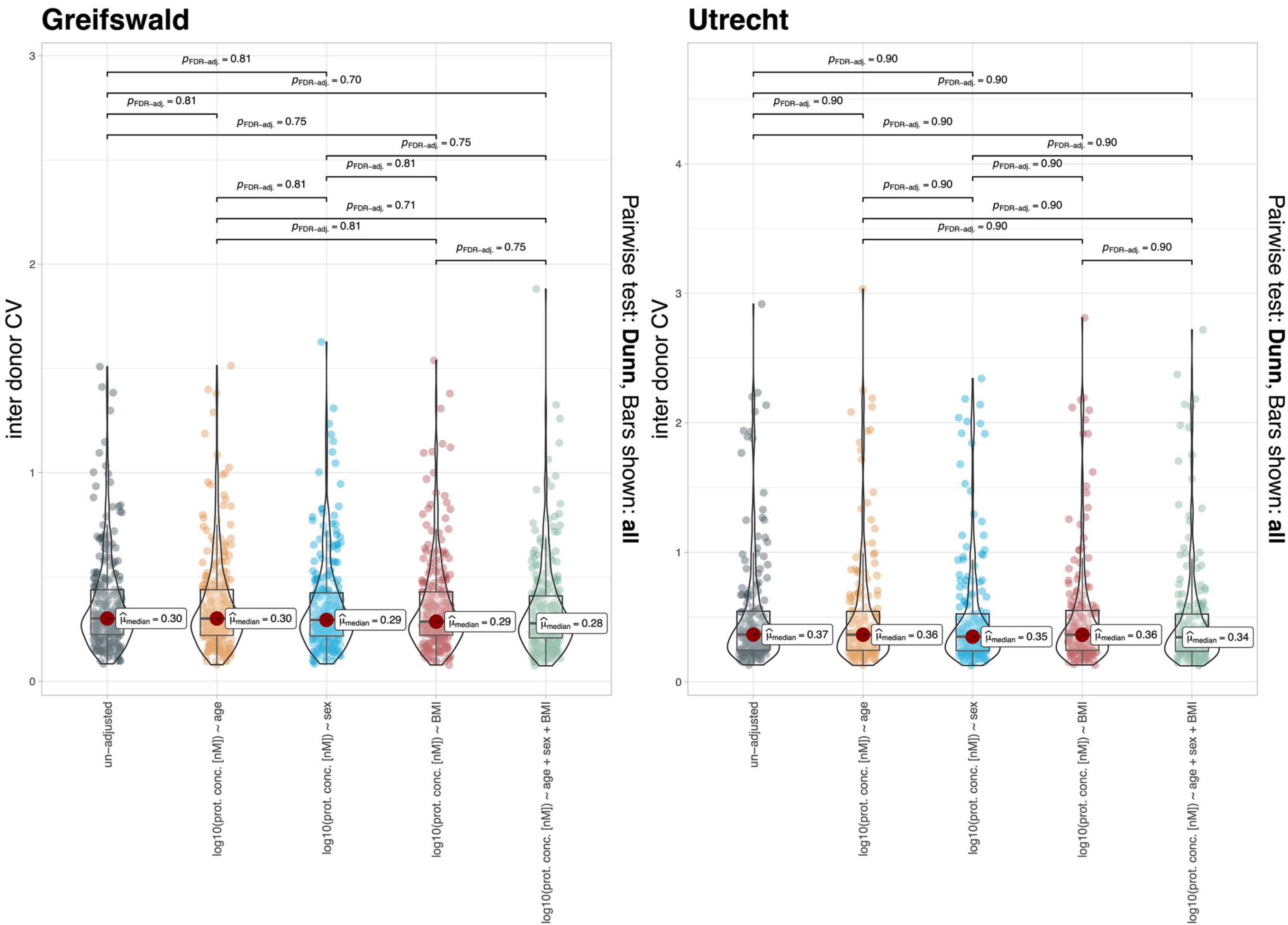

Figure EV2. Violin-Boxplot comparing the inter-donor coefficient of variation (CV) between confounder-adjusted concentration data of Greifswald site (left panel, $n = 274$ proteins per model) and Utrecht site (right panel, $n = 188$ proteins per model).

The x-axis shows the separate linear regression models for data adjustment, while the y-axis represents the inter-donor CV. The median CV for each model and lab site is highlighted, and its value is displayed. Additionally, the Dunn tests adjusted p-values for pairwise comparisons are presented. Box plots display the following statistical descriptors: center line, median; box bounds, 25th and 75th percentiles (interquartile range, IQR); whiskers, 1.5 × IQR below the 25th and above the 75th percentile; points beyond the whiskers represent outliers; minima and maxima indicate the smallest and largest observed values within the whisker range, respectively.

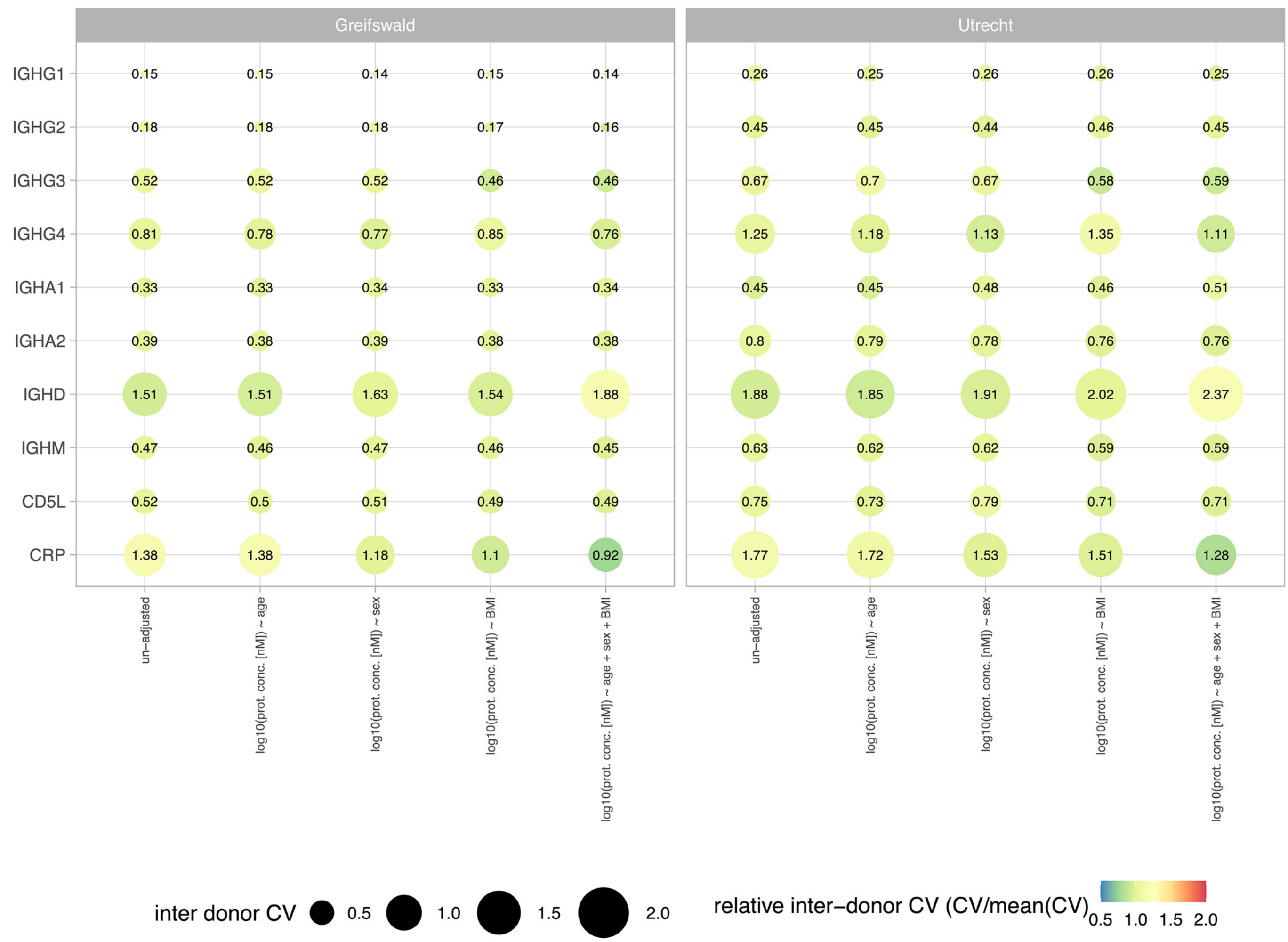

Figure EV3. Inter donor coefficient of variation (CV) plots for selected proteins, along with separate linear regression models.

The left panel represents the data from the Greifswald site, whereas the right panel represents the data from the Utrecht site. The size of the points represents the inter-donor CV, while the color of the points indicates the scaled relative per protein and site inter-donor CV. Additionally, the inter-donor CV is also displayed as a rounded number with two digits.

 © The Author(s)

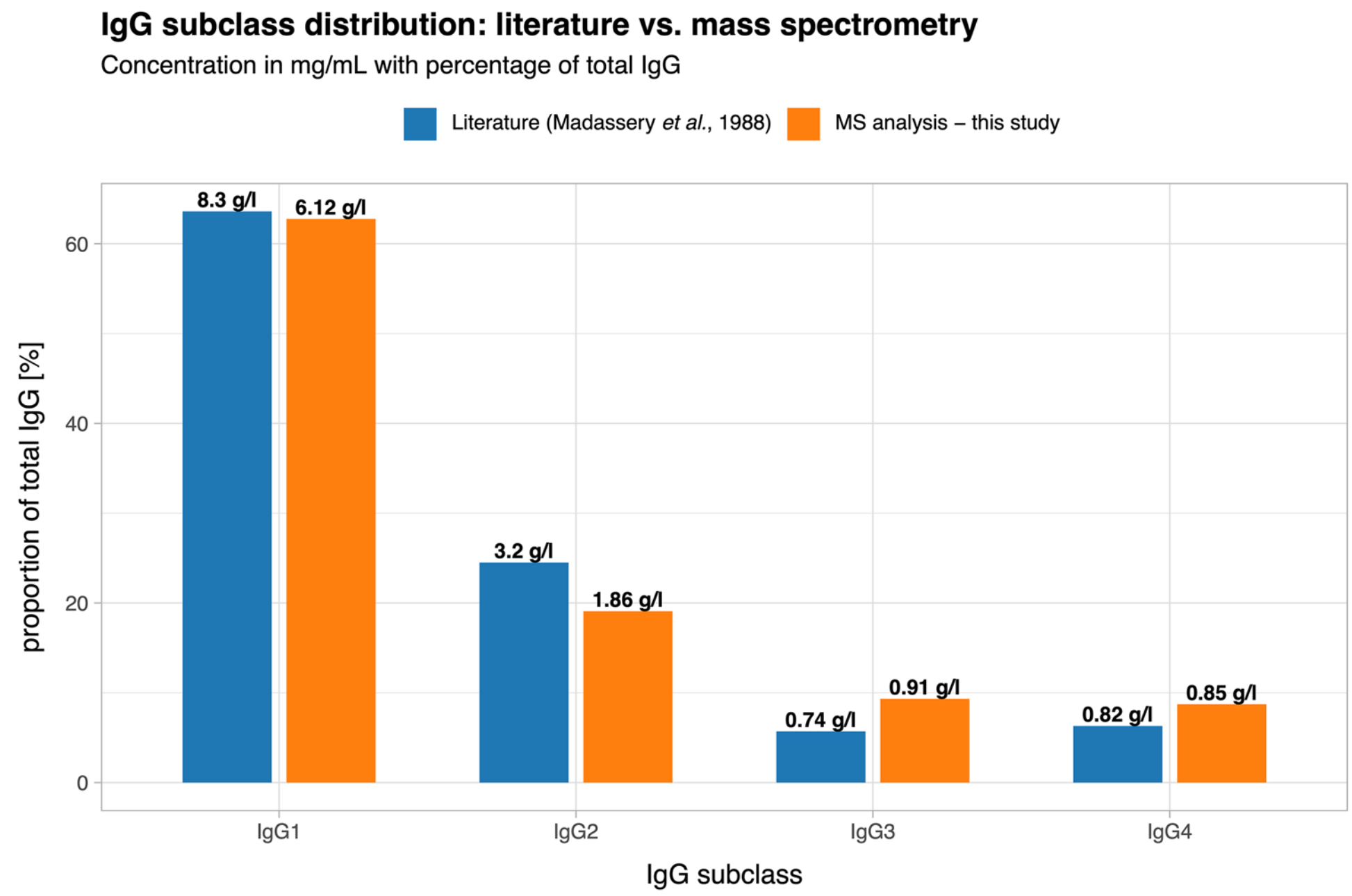

Figure EV4. Bar plot depicts the proportion of IgG subclasses known from literature (blue bars) and measured in the TIMES cohort via mass spectrometry analysis (orange bars).

© The Author(s)
